# Anti-Neuroinflammatory Eremophilane Sesquiterpenoids from Marine-Derived Fungus *Phoma* sp. DXH009

**DOI:** 10.3390/md23030094

**Published:** 2025-02-20

**Authors:** Guanyu Yang, Mengwei Qin, Mingbin Chen, Yujia Shi, Siyi Liu, Yong Rao, Ling Huang, Ying Fu

**Affiliations:** Key Laboratory of Tropical Biological Resources, Ministry of Education, School of Pharmaceutical Sciences, Hainan University, Haikou 570228, China; yangguanyu@hainanu.edu.cn (G.Y.); 22211007000035@hainanu.edu.cn (M.Q.); 22211007000020@hainanu.edu.cn (M.C.); 23221055000018@hainanu.edu.cn (Y.S.); lsy@hainanu.edu.cn (S.L.)

**Keywords:** eremophilane sesquiterpenoids, lipopolysaccharide, anti-neuroinflammatory, *Phoma* sp.

## Abstract

Three new eremophilane sesquiterpenoids (**1**–**3**), together with six known analogues, were isolated from the marine-derived fungus *Phoma* sp. DXH009. Their structures were elucidated through detailed NMR and MS spectroscopic analysis, and the absolute configurations of **1**–**4** were determined by conformational analysis and quantum chemical TDDFT-ECD calculation. Their anti-neuroinflammatory activities were evaluated using the lipopolysaccharide (LPS)-induced BV2 microglial cells. The results indicated that compound **5** (dihydrosporogen AO-1) exhibited significant inhibitory effects on the production of nitric oxide (NO) levels (EC_50_ = 3.11 μM) with less cytotoxicity, leading to a reversal effect in inducing microphage polarization in LPS-treated BV2 microglial cells. These were correlated with suppressions of the canonical NF-κB pathway as well as the expression levels of key neuroinflammatory markers, including COX2, TNF-α, IL-6, and IL-1β. Correspondingly, treating **5** in LPS-induced mice efficiently ameliorated neuroinflammation in the tissues of the cortex and hippocampus. These findings suggest that eremophilane sesquiterpenoid **5** could be a potential candidate for the development of anti-neuroinflammatory drugs.

## 1. Introduction

Neuroinflammation represents a dual-edged immunological process within the central nervous system (CNS), wherein microglia and astrocytes transition from homeostatic surveillance to a reactive state, secreting pro-inflammatory cytokines (e.g., IL-1β and TNF-α), chemokines, and neurotoxic reactive oxygen species (ROS) [1,2,3]. Although transient activation of this response is critical for neutralizing pathogens or repairing tissue damage, persistent glial reactivity drives a self-propagating cycle of neuronal injury, characterized by synaptic stripping, blood–brain barrier (BBB) compromise, and progressive circuit degeneration [4,5,6]. Growing evidence highlights neuroinflammation as a hub in the pathogenesis of neurodegenerative diseases such as Alzheimer’s disease, Parkinson’s disease, multiple sclerosis, stroke, and traumatic brain injury [7,8,9]. For instance, in Alzheimer‘s disease (AD), fibrillar amyloid-β (Aβ) deposits engage microglial pattern recognition receptors (e.g., TLR4), leading to NLRP3 inflammasome activation, which exacerbates tau hyperphosphorylation and cognitive deficits in murine models [10,11]. Parkinson‘s disease (PD) pathology similarly involves α-synuclein-mediated conversion of astrocytes to a neurotoxic A1 phenotype, accelerating dopaminergic neuron loss in the substantia nigra [12,13].

Therapeutic modulation of neuroinflammatory cascades is now recognized as a promising strategy to halt or reverse neurodegenerative pathology. The small-molecule NLRP3 inhibitor MCC950 (CRID3) attenuates hippocampal IL-1β production by 68.5% in APP/PS1 transgenic mice, concomitant with preserved novel object recognition performance [14,15]. Natural products with pleiotropic anti-inflammatory properties are equally promising: curcumin, a diarylheptanoid derived from *Curcuma longa*, demonstrates multimodal anti-neuroinflammatory effects by suppressing NF-κB signaling (IC_50_ = 4.7 μM in BV2 microglia) and enhances Aβ clearance via TREM2 upregulation, yielding a 37.2% improvement in radial arm maze errors relative to untreated AD mice [16,17]. Resveratrol, a naturally derived SIRT1 activator from grapes and berries, achieved a 31.6% reduction in CSF TNF-α levels among early-stage PD participants (Phase II trial, NCT04542915) without significant adverse events [18]. These developments emphasize the therapeutic promise of neuroinflammation-targeting agents in curbing the progression of neurodegenerative diseases.

Eremophilane sesquiterpenes are widely found in many plants (such as *Compositae* sp. and *Labiaceae* sp.) and fungi (such as *Penicillium* sp. [19], *Xylaria* sp. [20], *Phoma* sp. [21], etc.). About 200 different eremophilane sesquiterpenoids have been reported from various fungi [22]. A minority of them have been detected both in fungi and plants [23]. This fact highlights the uniqueness of fungus-derived eremophilane sesquiterpenes, characterized by a 6/6-bicyclic carbon skeleton with multiple chiral centers [24], leading to diverse biological activities such as antibacterial [25], herbicidal [26], anti-inflammatory [27,28], anti-oxidative, anti-tumor [29] and neuroprotective [30] activities. These properties have gradually attracted the attention of researchers for their potential application in neurological diseases. An eremophilane sesquiterpenoid named septoreremophilane F exhibited notable anti-neuroinflammatory activity in LPS-activated BV-2 microglia, suppressing nitric oxide production with an IC_50_ value of 12.0 ± 0.32 μM, while computational docking studies further suggested its potential interaction with iNOS protein [31], underscoring the therapeutic relevance of this structural class in CNS disorder drug discovery.

In our search for novel terpenoids with potent neuroinflammatory inhibition activity, the marine-derived fungus *Phoma* sp. DXH009 was investigated, leading to isolation of nine eremophilane sesquiterpenes (**1**–**9**). Their anti-neuroinflammatory effects were assessed by the inhibition of nitric oxide (NO) production in LPS-stimulated BV2 microglial cells. This study reported the separation, structure elucidation, anti-neuroinflammatory activity evaluation, structure–activity relationship analysis, and mechanistic investigation of these eremophilane sesquiterpenes.

## 2. Results and Discussion

**Figure 1:**
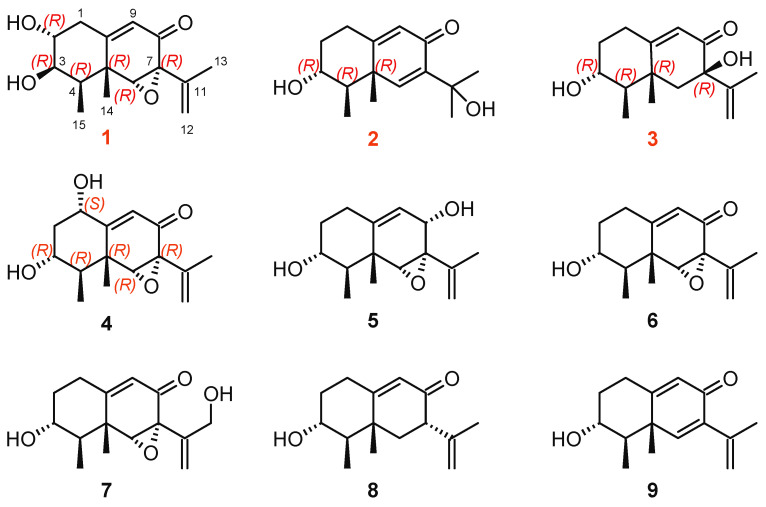
Structures of compounds **1**–**9**.

A series of eremophilane sesquiterpenes (**1**–**9**) (Figure 1) were isolated from the ethyl acetate extract of the marine-derived fungus *Phoma* sp. DXH009, with yields ranging from 2.5 mg (0.00868%) to 100 mg (0.347%), via sequential silica gel chromatography, ODS column fractionation, and semi-preparative HPLC purification (see Section 3.3 for full protocols and detailed yields).

### 2.1. Structure Elucidation

**Figure 2:**
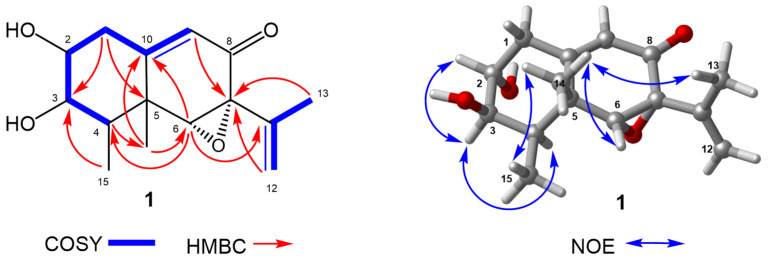
Key ^1^H–^1^H COSY, HMBC, and NOE correlations of **1**.

Phomenone C (**1**) was isolated as a colorless oil. Its molecular formula was assigned as C_15_H_20_O_4_ with six degrees of unsaturation by the ion peaks at *m*/*z* 287.1260 ([M + Na]^+^, calcd. for 287.1259) and 551.2621 ([2M + Na]^+^, calcd. for 551.2617) in the HRESIMS spectrum. The 1D NMR data (Table 1) combined with DEPT and HSQC spectra of **1** indicated the presence of a carbonyl group (*δ*_C_ 192.7), a trisubstituted olefinic bond (*δ*_C_/*δ*_H_ 123.0/5.81, *δ*_C_ 159.5), a terminal vinyl (*δ*_C_/*δ*_H_ 114.8/5.11, *δ*_C_ 139.0), four sp^3^ methines (one at *δ*_C_/*δ*_H_ 40.5/1.86 and three oxygenated methines at *δ*_C_/*δ*_H_ 74.1/3.49, 74.1/3.42, and 68.0/3.32), one sp^3^ methylene (*δ*_C_/*δ*_H_ 38.9/2.60), three methyls (*δ*_C_/*δ*_H_ 19.9/1.86, 19.0/1.25, and 11.3/1.27) and an oxygenated quaternary carbon (*δ*_C_ 63.6). The six degrees of unsaturation, except for a carbonyl group and two double bonds, meant that **1** contained three rings. Four oxygen atoms should satisfy four oxygenated sp^3^ carbons and a carbonyl group; thus, **1** has an epoxy ring and two hydroxyls. These spectroscopic features suggested that **1** was a new *8*-*one*-eremophilane sesquiterpenoid, which was confirmed by the ^1^H–^1^H COSY and HMBC spectra. The ^1^H–^1^H COSY spectrum (Figure 2) revealed two spin systems consisting of H-9/H_2_-1/H-2/H-3/H-4 and H_2_-12/H_3_-13, as well as the key HMBC correlations of H_3_-14 to C-6 and C-10; H-6 to C-4, C-10, and C-11; H-4 to C-5; H-9 to C-7; H_2_-12 to C-7; and H_3_-13 to C-7, as shown in Figure 2, to establish the planar structure of **1**. The relative configuration of **1** was determined based on extensive analysis of NOE correlations (Figure 2). NOEs between H_3_-14 and H_3_-15, H_3_-13, and H-6 revealed their cofacial relationship and were assigned as β-orientation, whereas H-4 was α-oriented. The big coupling constants (both 9.0 Hz) of *J*_H-2,H-3_ and *J*_H-3,H-4_ (Table 1) revealed that the dihedral torsion angles of H-2–C-2–C-3–H-3 and H-3–C-3–C-4–H-4 were both either small (less than 45°) or big (more than 120°) according to the Karplus equation [32]. The NOE interaction of H-3/H-4 and the absence correlation of H-3/H_3_-15 indicated a small dihedral angle of H-3–C-3–C-4–H-4, which concluded the α-oriented H-3. Similarly, a small dihedral angle of H-2–C-2–C-3–H-3 was deduced by the NOE interaction of H-3/H-2 and an α-oriented H-3. Then, the undetected NOE correlation of H-2/H-4 ascertained the β-oriented H-2.

**Figure 3:**
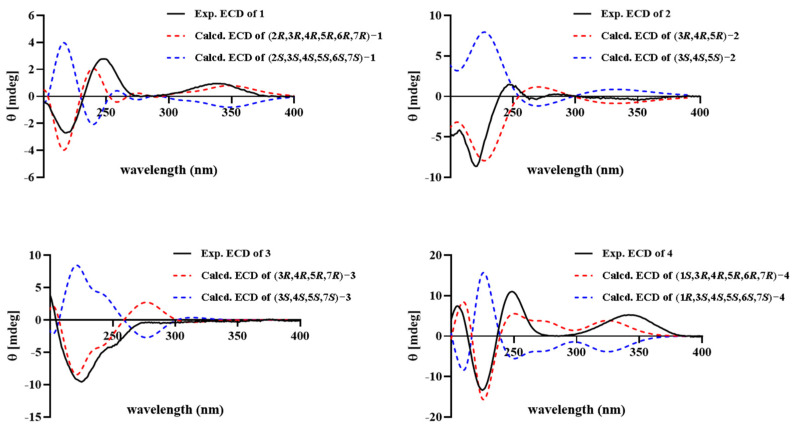
Experimental and calculated ECD spectra of **1**–**4**.

The absolute configuration of **1** was explicitly assigned by quantum-chemical ECD calculation. The ECD spectra for the predominant conformers **1**-1–**1**-5 were calculated by TDDFT methodology at the B3LYP/TZVP level. The nice fit of the calculated ECD curve of 2*R*,3*R*,4*R*,5*R*,6*R*,7*R*-configured **1** with the experimental ECD spectrum (Figure 3) ascertained the absolute configuration of **1** as depicted, and we named it phomenone C.

**Figure 4:**
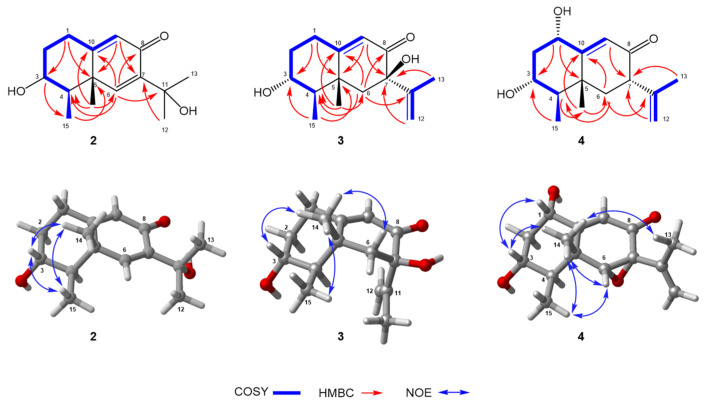
Key ^1^H–^1^H COSY, HMBC, and NOESY correlations of **2**–**4**.

Phomenone D (**2**), a colorless oil, had a molecular formula of C_15_H_22_O_3_ with five degrees of unsaturation, which was established by the HR-ESI-MS spectrum with a positive ion at *m*/*z* 273.1464 ([M + Na]^+^, calcd. for 273.1467) and 523.3042 ([2M + Na]^+^, calcd. for C_30_H_44_NaO_6_, 523.3036). The ^1^H and ^13^C NMR spectroscopic data (Table 1) resembled those of petasol (**8**) [33]; the difference was the addition of a hydroxyl group at C-11 (*δ*_C_ 72.4) and the double bond shift from C-11–C-12 to C-6–C-7 (*δ*_C-6_/*δ*_H-6_ 151.6/7.31, *δ*_C-7_ 143.1) in **2**, which were corroborated by key HMBC correlations from H-6 to C-4, C-8, C-10, and C-11; from H-9 to C-5 and C-7; and from H_3_-14 to C-4, C-6, and C-10 (Figure 4). Thus, the planar structure of **2** was identified as shown in Figure 4 and found to be the same as a chemical reaction product named petasitol [34] only speculated by partial ^1^H NMR data. Then, the relative configuration of **2** was established on the basis of diagnostic NOE interactions. NOEs between H-3/H_3_-14, H-3/H_3_-15, H-3/H-1β, and H_3_-14/H-1β revealed their cofacial relationship and assigned the β-oriented H-3, H_3_C-4, and H_3_C-5, whereas it concluded the α-oriented 3-OH. The absolute configuration of **2**, named phomenone D, was determined by ECD calculation. The computed ECD curve of 3*R*,4*R*,5*R*-**2** was in good agreement with the experimental ECD spectrum (Figure 3), thus establishing the 3*R*,4*R*,5*R* configuration for **2**.

Hydroxypetasol (**3**) gave the same molecular formula of C_15_H_22_O_3_ as **2** with five degrees of unsaturation according to the HR-ESI-MS positive ion peaks at *m*/*z* 273.1467 ([M + Na]^+^, calcd. for 273.1467) and *m*/*z* 523.3031 ([2M + Na]^+^, calcd. for 523.3036). The ^1^H and ^13^C NMR spectroscopic data (Table 1) of **3** revealed that its structure was similar to that of petasol (**8**) [33] with the difference of an additional hydroxyl group at C-7 in **3**, which was confirmed by HMBC correlations from H-9, H_2_-12, and H_3_-13 to the quaternary carbon C-7 (*δ*_C_ 78.2). Key correlations in the ^1^H-^1^H COSY and HMBC spectra (Figure 4) identified **3** to be 7-hydroxyl-substituted petasol, the planar structure of which was previously reported and only deduced by ^1^H NMR data. Herein, its relative configuration was assigned by the NOE interactions (Figure 4) of H_3_-14 with H-3, H_3_-15, H-6β, and H_3_-13 with H-6α, illustrating that H-3, H_3_-14, and H_3_-15 were all on the same side, while H_3_-13 was on their opposite side. Then, the ECD spectra for the predominant conformers **3**-1–**3**-5 were calculated to obtain the computed ECD curve. The absolute configuration of 3, named hydroxypetasol, was established to be 3*R*,4*R*,5*R*,7*R* (Figure S2), since its calculated spectrum matched well with the experimental ECD spectrum for **3** (Figure 3).

The molecular formula of graphilane (**4**) was identified to be C_15_H_22_O_3_ (six degrees of unsaturation) deduced by the HR-ESI-MS positive ion peak at *m*/*z* 255.1360 ([M + Na]^+^, calcd. for C_15_H_20_NaO_4_, 255.1361) and *m*/*z* 487.2818 ([2M + Na]^+^, calcd. for C_30_H_40_NaO_8_, 487.2824). According to the ^1^H and ^13^C NMR spectroscopic data of **4** (Table 1), its planar structure and relative configuration were both the same as a known eremophilane sesquiterpenoid named graphilane [35], which was found by comparing the 1D NMR spectroscopic data of **4** with that of graphilane. The planar structure of **4** was further confirmed by the key correlations in the ^1^H-^1^H COSY and HMBC spectra (Figure 4). Its relative configuration (Figure 4) was assigned by key NOE correlations of H_3_-14 with H_3_-15, H_3_-13, H-3, H-6, and H-3 with H-1, which revealed their β-orientation. Finally, the calculated ECD curve for the predominant conformers **4**-1–**4**-5 of 1*S*,3*R*,4*R*,5*R*,6*R*,7*R*-**4** fit well with the experimental ECD spectrum (Figure 3), identifying its absolute configuration as 1*S*,3*R*,4*R*,5*R*,6*R*,7*R*.

The five known compounds, dihydrosporogen AO-1 (**5**) [36], sporogen AO-1 (**6**) [37], phomenone (**7**) [38], petasol (**8**) [33], and 6-dehydropetasol (**9**) [39], were determined by comparison with the spectroscopic data from the literature. Compounds **1**–**9** are *3*-*hydroxyl*-*9*-*ene*-eremophilane sesquiterpenoids with an additional hydroxyl group substituted at various positions and a double bond or an epoxy group substituted at C-6–C-7. Among them, **1**–**3** are new compounds. Compound **1** is a new *3*-*hydroxyl*-*8*-*one*-*9*-*ene*-eremophilane sesquiterpene, since the hydroxyl group at C-13 in **7** is transferred to C-2 in **1**. Moreover, 3-β-OH in 1 is quite different from the common eremophilane sesquiterpenes, despite the *R*-configuration at C-3 being maintained. For compounds **2**–**4**, we have first confirmed their exact structures and absolute configurations through detailed NMR data analysis and ECD calculation. There have been no reports on the anti-neuroinflammatory activities of compounds **1**–**9**.

### 2.2. Biological Activity

#### 2.2.1. Evaluation of the Anti-Inflammatory Effects of Compounds in LPS-Treated Microglia Cells

**Figure 5:**
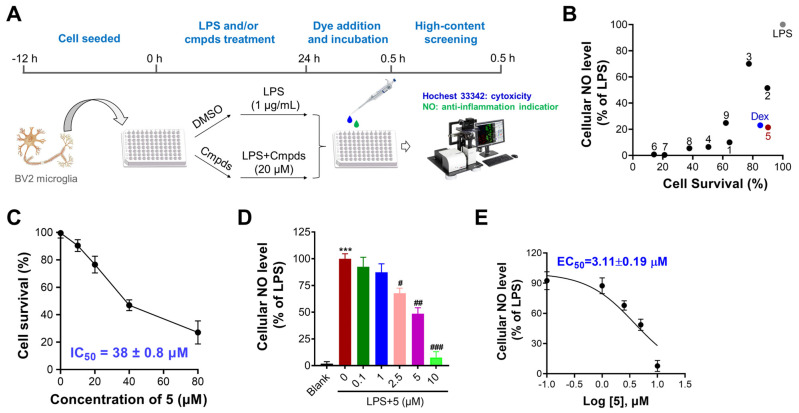
Identification of **5** as a potent neuroinflammatory suppressor in LPS-treated BV2 microglia cells through high throughput screening. (**A**). An illustration of high throughput screening of neuroinflammatory suppressor by examination of cellular NO production. (**B**). Examination of cellular NO levels and cell survival rate upon compounds (20 μM) treatment for 24 h. (**C**). Does-dependent effect of **5** against BV2 cell proliferation. (**D**). Does-dependent effect of **5** in decreasing cellular NO levels. (**E**). Calculation of EC_50_ value of **5** in decreasing NO production. *n* = 3 independent biological experiments. Data are expressed as “mean ± sd”. *** *p* < 0.001, vs. control cells. ^#^ *p* < 0.05, ^##^ *p* < 0.01, ^###^ *p* < 0.001 vs. LPS-treated control cells.

The anti-neuroinflammatory activity of nine isolated compounds was evaluated in LPS-stimulated microglia BV2 cells by determining their NO-lowering and cytotoxicity efficacies with dexamethasone (Dex) serving as a positive control; the screening diagram is depicted in Figure 5A. Compared with LPS control cells, Dex treatment efficiently decreased NO content with a 77% reduction in the NO level at 20 µM. Most of the compounds efficiently decreased cellular NO content with reductions ranging from ~10% to ~90% (Figure 5B). Among these, compound **5** displayed strong efficacy with a 78% reduction in NO content at 20 μM and less cytotoxicity against microglia BV2 cells.

Compound **5** (Dihydrosporogen AO-1) is a natural compound first isolated in 1988 from the fungus *Alternaria cityi*. Its chemical structure, including absolute configuration, was elucidated using circular dichroism (CD) spectroscopy [40]. Dihydrosporogen AO-1 has been reported to exhibit potent herbicidal effects against weeds such as *Amaranthus hypochondriacus* (IC_50_ = 0.17 mM), *Echinochloa crus-galli* (IC_50_ = 0.30 mM) [26], and *Lactuca sativa* (70% inhibition at 250 ppm) [40], specifically targeting radicle growth. Moreover, compound **5** has shown cytotoxicity against Ehrlich carcinoma cells (ED_50_ = 0.4 mM) [41], while it displays low toxicity toward noncancerous Vero cells (IC_50_ = 24.05 mM) [42], suggesting a favorable therapeutic window. Despite its established bioactivity in herbicidal and anticancer contexts, no studies to date have investigated the anti-neuroinflammatory properties of dihydrosporogen AO-1. Subsequently, the dose-dependent effects of **5** in decreasing cellular NO content and inhibiting BV2 cell proliferation were determined. As shown in Figure 5C–E, compound **5** showed potent inhibitory activity in decreasing cellular NO content with an EC_50_ value of 3.11 μM and acceptable cytotoxicity with an IC_50_ value of 38 μM. Additionally, compound **5** has a higher yield (100 mg) for biological evaluation; thus, **5** was selected for further study.

#### 2.2.2. Compound **5** Inhibits Microphage Depolarization Alongside NF-κB Signaling Suppression

**Figure 6:**
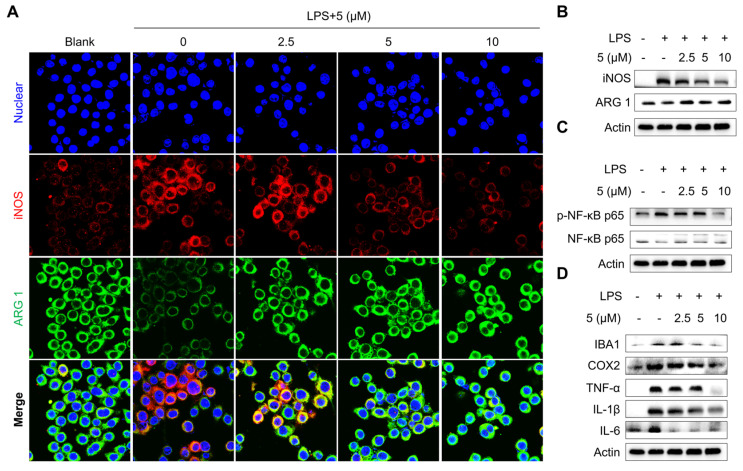
Compound **5** inhibits microphage polarization in LPS-treated BV2 microglia cells. BV2 microglia cells were exposed to LPS (0.5 μg/mL) induction for 24 h in the presence or absence of **5** (2.5, 5, 10 μM) treatment for 24 h. (**A**). Immunofluorescence analysis of ARG 1 and iNOS, and quantification. Scale bar, 30 μm. (**B**). Immunoblot analysis of ARG 1 and iNOS, and quantification. (**C**)**.** Examination of NF-κB p65 signaling pathway. (**D**). Examination of inflammatory markers. *n* = 3 independent biological experiments. Data are expressed as “mean ± sd”.

Microglia, the resident immune cells of the central nervous system, exist in a resting state (M0 phenotype) under normal physiological conditions and swiftly transition to activated states, such as classically activated states (M1-polarized microglia) and alternatively activated states (M2-polarized microglia). The former releases pro-inflammatory cytokines and neurotoxic substances to neutralize pathogens, playing a critical role in initiating immune responses, while the latter produces anti-inflammatory factors, which is crucial for restoring brain homeostasis after injury or disease. We then determined the microglia depolarization by determining the representative markers including arginase 1 (ARG 1) (M2 microglia) and iNOS (M1 microglia). As shown in Figure 6A, LPS treatment markedly increased iNOS protein levels but decreased the expression level of ARG 1. Analysis of the ratio of ARG 1 to iNOS revealed that LPS treatment decreased the ratio, which implies that LPS treatment induced BV2 microglia transforming into the M1 depolarized state. The addition of compound **5** dose-dependently reversed iNOS elevations and ARG 1, which decreased to levels similar to those observed in LPS-untreated BV2 cells. In addition, **5** corrected the decreased ratio of ARG 1 to iNOS induced by LPS treatment. Meanwhile, immunoblot analysis further confirmed the correction effect of **5** in BV2 microglia cells (Figure 6B), suggesting that **5** treatment induced BV2 microglia transition to an M2-polarized state.

NF-κB is a classic transcriptional regulator that is involved in inflammation regulation by directly binding to the promoter region of cytokine genes. Previous studies have demonstrated that inhibition of NF-κB signaling is beneficial for neuroinflammation inhibition in treating neurodegenerative diseases [43]. We were eager to examine the inhibition effect of **5** in NF-κB signaling, and expectedly found that **5** treatment dose-dependently reversed the activation of the NF-κB axis as evident by decreases in the phosphorylated level of NF-κB p65, while the total level of NF-κB p65 showed no major change (Figure 6C). In conclusion, treating **5** in LPS-treated microglia cells efficiently reversed the increases in the level of inflammatory markers, including IBA1, COX-2, TNF-α, IL-1β, and IL-6 (Figure 6D).

#### 2.2.3. Compound **5** Attenuates Neuroinflammation in the Cortex and Hippocampus of LPS-Treated Mice

**Figure 7:**
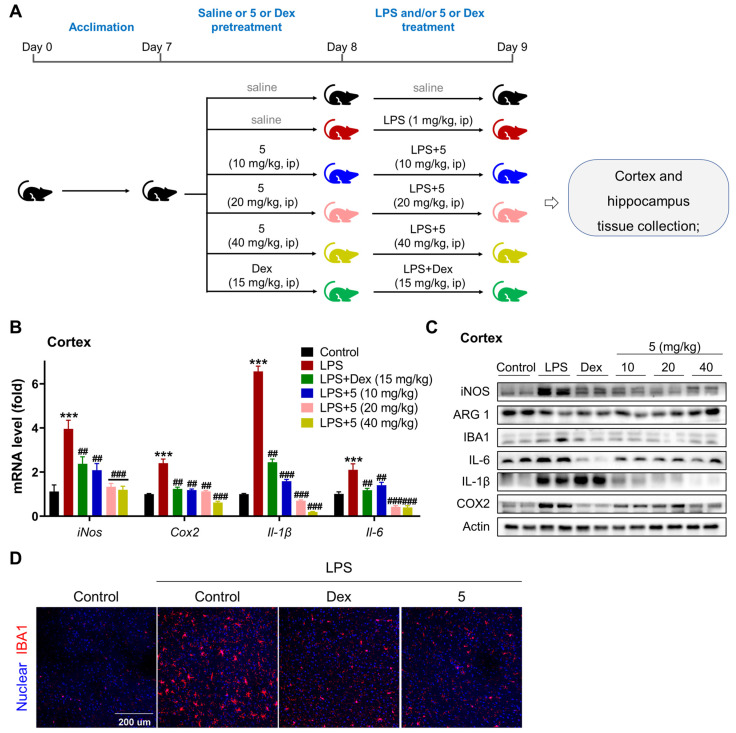
Compound **5** inhibits neuroinflammation in the cortex tissue of LPS-treated mice. (**A**). An illustration of the animal experiment. (**B**). mRNA levels of key inflammatory markers in the cortex by qPCR. (**C**). Protein levels of key inflammatory markers in the tissue of the cortex by Western blot. (**D**). Immunofluorescence analysis of IBA1 in the cortex. Scale bar, 200 μm. *n* = 10–12 mice/group. Data are expressed as “mean ± sd”. *** *p* < 0.001 vs. saline group mice. ^##^ *p* < 0.01, ^###^ *p* < 0.001 vs. LPS-treated control mice.

**Figure 8:**
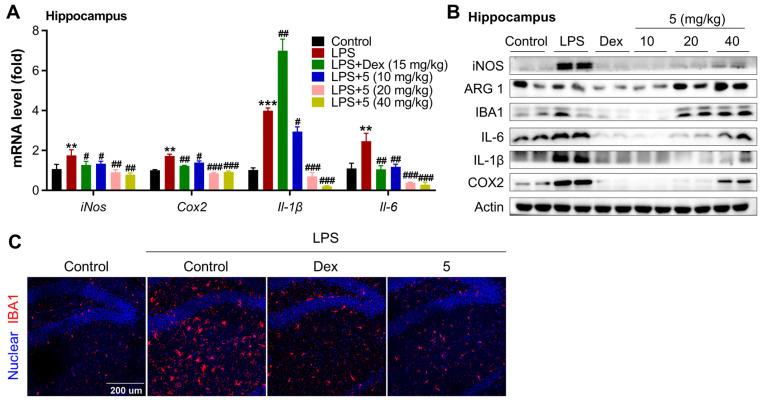
Compound **5** inhibits neuroinflammation in the hippocampus tissue of LPS-treated mice. (**A**). mRNA levels of key inflammatory markers in the hippocampus by qPCR. (**B**). Protein levels of key inflammatory markers in the hippocampus by Western blot. (**C**). Immunofluorescence analysis of IBA1 in the tissue of the hippocampus. Scale bar, 200 μm. *n* = 10–12 mice/group. Data are expressed as “mean ± sd”. ** *p* < 0.01, *** *p* < 0.001 vs. saline group mice. ^#^ *p* < 0.05, ^##^ *p* < 0.01, ^###^ *p* < 0.001 vs. LPS-treated control mice.

We then determined the anti-neuroinflammatory effect of **5** in LPS-treated mice. After a week of acclimation, mice were treated with saline or **5** (10, 20, 40 mg/kg) or Dex (15 mg/kg) once through intraperitoneal injection, then co-treated with LPS or LPS + **5** (10, 20, 40 mg/kg) or LPS + Dex (15 mg/kg) once. The animal experimental procedure is depicted in Figure 7A. LPS treatment markedly increased the expression levels of inflammatory markers in the tissue of the cortex, including iNOS, COX2, IL-1β, and IL-6 at both transcriptional and translational levels (Figure 7B,C). Treatment with either **5** or Dex in mice efficiently suppressed the activation of these inflammatory markers in the tissue of the cortex upon LPS induction. Also, examination of ARG 1 in the cortex, a marker of M2 microglia, demonstrated that treatment with **5** dose-dependently increased the protein level of ARG 1, while no induction effect was observed in Dex-treated mice. Correspondingly, analyzing the bio-marker of activated microglia in the cortex revealed that either **5** or Dex treatment markedly decreased the expression levels of IBA1 (Figure 7C,D). These inflammatory suppression effects also occurred in the tissue of the hippocampus (Figure 8). These data together indicate that **5** is a promising candidate for developing therapeutic agents against neuroinflammation-association neurodegeneration disease. For the dose-dependency bias of the actions of compound **5** in the cortex and hippocampus, we reasonably believe it may correlate with the LPS-induced acute neuroinflammation mouse model, as the action and biological function of cortex and hippocampus tissues are different when responding to inflammatory stimulation. Also, this bias may link with its direct target of compound **5** in the tissues. Furthermore, for a drug to exert its therapeutic effects in the central nervous system (CNS), it must be capable of crossing the blood–brain barrier (BBB). Therefore, determining the BBB permeability of compounds becomes critically important during the initial stages of CNS-targeted drug design and development. The BBB penetrability assay for compound **5** will be conducted as a priority in our subsequent research.

### 2.3. Structure–Activity Relationship (SAR) Analysis of Compounds ***1***–***9***

Systematic analysis of substituent effects for the 3-*hydroxy*-9,10,-*ene*-eremophilane scaffold, shared by all isolated compounds (**1**–**9**), revealed the following structure–activity relationships: (1) Essential pharmacophores: The C-6–C-7 unsaturated moiety (double bond or epoxy group) and C-11–C-12 terminal unsaturation were critical for anti-inflammatory potency. Disruption of these motifs (e.g., C-6–C-7 hydroxylation in **2** or saturation in **3**) resulted in a high reduction in NO inhibition. (2) Cytotoxicity modulation: Epoxidation at C-6–C-7 combined with an 8-*keto* group (**1**, **4**, **6**–**9**) induced significant cytotoxicity (approximately 35–85% proliferation inhibition at 20 μM), leading to non-specific NO suppression. Reduction of the 8-*keto* to a hydroxyl group (**5**) retained anti-inflammatory efficacy (78% NO reduction at 20 μM; EC_50_ = 3.11 μM) while reducing cytotoxicity (IC_50_ = 38 μM), achieving a favorable selectivity index (SI 12.2).

These findings demonstrate the SAR of anti-neuroinflammatory activity and cytotoxicity for 3-*hydroxy*-9,10,-*ene*-eremophilane core, notably highlighting the critical roles of 6,7-*epoxy*, 11,12-*ene*, and 8-*keto* reduction. While direct comparisons are limited by the absence of prior SAR data for similar eremophilanes, we believe this work establishes a foundational framework for further optimization of 3-*hydroxy*-9,10,-*ene*-eremophilanes. To validate our proposed structural determinants, future studies such as systematic analogue synthesis and biological evaluation should be investigated.

## 3. Materials and Methods

### 3.1. General Experimental Procedures

HRESIMS data were detected by a UPLC-MS spectrometer (Agilent 6546LC/Q-TOF, Ontario, CA, USA). The 1D and 2D NMR spectra were measured on a Bruker AV-400 MHz NMR spectrometer (Bruker Biospin AG, Fällanden, Zürich, Switzerland). Optical rotations were determined on a MCP 5100 modular circular polarimeter (Anton Paar GmbH, Graz, Austria) with a 1.0 dm cell at 25 °C. A circular dichroism spectrometer (Chirascan V100, Applied Photophysics Ltd., Leatherhead, UK) was used to obtain UV and ECD data. Preparative HPLC was performed on a CXTH P3000 pump equipped with a YMC-Pack ODS-AQ (250 × 20 mm, 5 μm, 12 nm) and a UV3000 detector (CXTH Ltd., Beijing, China). The column chromatography was carried out with silica gel (100–200 mesh, Qingdao Marine Chemical Ltd., Qingdao, China) and chromatorex (C_18_ SMB100, 20–45 μm, Fuji, Japan).

### 3.2. Fungal Material

The fungal strain, *Phoma* sp. DXH009, was isolated from *Conus litteratus* collected from the Xisha Islands in the South China Sea. This fungus was identified as *Phoma* species based on the ITS region sequence (GenBank accession no. PQ056952), with 99.81% similarity to *Phoma* sp. strain DTO-421-F6. The strain was preserved at −80 °C in the Key Laboratory of Tropical Biological Resources of Ministry of Education, School of Pharmaceutical Sciences, Hainan University, Haikou, P.R. China.

### 3.3. Fermentation, Extraction, and Isolation

The fungus strain was cultured on potato dextrose agar (PDA) at 28 °C for 3 days after transferring it from −80 °C. Several pieces of agar blocks with mycelia were inoculated into 1000 mL Erlenmeyer flasks containing 300 mL of potato dextrose broth (PDB; 20% potato, 2% glucose, 0.3% sea salt). Subsequently, these flasks were incubated on a rotary shaker (180 rpm) at 28 °C for 3 days to gain seed culture. Large-scale fermentation was conducted on two hundred cylindrical wide-mouth bottles (500 mL) of solid rice medium. Each bottle contained 80 g rice, 90 mL distilled water, and 0.36 g sea salt, and was sterilized at 121 °C for 21 min before inoculation. Each bottle with rice medium was inoculated in 10 mL seed culture and then incubated at 28 °C for 30 days under static conditions.

The fermentation material was extracted three times with ethanol at room temperature to yield an extract, which was then suspended in water and partitioned successively with petroleum ether and ethyl acetate, respectively. The ethyl acetate portion (28.8 g) was eluted using silica gel (100–200 mesh) column chromatography with a gradient mixture of CHCl_3_/MeOH (200:1 to 0:1, *v*:*v*) and divided into 17 fractions (Fr.1–Fr.17).

Fr.7 (435.0 mg) was further separated by C_18_ reversed-phase silica gel column chromatography, eluted with a gradient mixture of MeOH/H_2_O (10:90 to 100:0, *v*:*v*), to give 6 subfractions. Fr.7-2 was purified by semi-preparative HPLC (MeCN/H_2_O, 15:85, 3.0 mL/min) to yield **3** (*t*_R_ = 33.5 min, 10.1 mg), whereas Fr.7-4 was subjected to semi-preparative HPLC with isogradient 30% MeCN aqueous solution (3.0 mL/min) to give **6** (*t*_R_ = 26.0 min, 8.0 mg). Compounds **8** (*t*_R_ = 15.0 min, 4.7 mg) and **9** (*t*_R_ = 16.0 min, 2.5 mg) were isolated from Fr.7-5 by semi-preparative HPLC (MeCN/H_2_O, 25:75, 3.0 mL/min), respectively. Fr.9 (184.0 mg) was applied to isogradient preparative HPLC eluted with 15% MeOH aqueous solution (10.0 mL/min) to gain **2** (*t*_R_ = 55.0 min, 3.0 mg). Fr.11 (463.0 mg) was separated by preparative HPLC (MeOH/H_2_O, 45:55, 10.0 mL/min) to afford **5** (*t*_R_ = 26.5 min, 100.0 mg). Compounds **1** (*t*_R_ = 42.5 min, 2.7 mg) and **7** (*t*_R_ = 75.5 min, 8.0 mg) were obtained from Fr.12 (1.18 g) by preparative HPLC (MeOH/H_2_O, 35:65, 10.0 mL/min), while **4** (*t*_R_ = 34.5 min, 24.0 mg) was gained from Fr.13 (482.0 mg) by preparative HPLC (MeOH/H_2_O, 25:75, 10.0 mL/min).

#### 3.3.1. Phomenone C (**1**)

Colorless oil; [α] + 87.0 (*c* 0.1, MeOH); UV (MeOH) *λ*_max_ (log*ε*) 195 (3.89) nm; CD (0.3 mM, MeCN) *λ*_max_ (Δ*ε*) 218 (−2.7), 247 (+2.8), 339 (+0.96) nm; ^1^H and ^13^C NMR spectroscopic data (see Table 1); HRESIMS *m*/*z* 287.1260 [M + Na]^+^ (calcd. for C_15_H_20_NaO_4_, 287.1259), 551.2621 [2M + Na]^+^ (calcd. for C_30_H_40_NaO_8_, 551.2617).

#### 3.3.2. Phomenone D (**2**)

Colorless oil; [α]—6.0 (*c* 0.1, MeOH); UV (MeOH) *λ*_max_ (log*ε*) 244 (4.10) nm; CD (0.3 mM, MeCN) *λ*_max_ (Δ*ε*) 220 (−8.6), 247 (+1.5) nm; ^1^H and ^13^C NMR spectroscopic data (see Table 1); HRESIMS *m*/*z* 273.1464 [M + Na]^+^ (calcd. for C_15_H_22_NaO_3_, 273.1467), 523.3042 [2M + Na]^+^ (calcd. for C_30_H_44_NaO_6_, 523.3036).

#### 3.3.3. Hydroxypetasol (**3**)

Colorless oil; [α]—60.0 (*c* 0.1, MeOH); UV (MeOH) *λ*_max_ (log*ε*) 238 (4.00) nm; CD (0.3 mM, MeCN) *λ*_max_ (Δ*ε*) 225 (−9.6) nm; ^1^H and ^13^C NMR spectroscopic data (see Table 1); HRESIMS *m*/*z* 273.1467 [M + Na]^+^ (calcd. for C_15_H_22_NaO_3_, 273.1467), 523.3031 [2M + Na]^+^ (calcd. for C_30_H_44_NaO_6_, 523.3036).

#### 3.3.4. Graphilane (**4**)

Colorless oil; [α] + 175.0 (*c* 0.1, MeOH); UV (MeOH) *λ*_max_ (log*ε*) 240 (4.00) nm; CD (0.3 mM, MeCN) *λ*_max_ (Δ*ε*) 205 (+7.4), 225 (−13.3), 249 (+11.0), 342 (+5.3) nm; ^1^H and ^13^C NMR spectroscopic data (see Table 1); HRESIMS *m*/*z* 255.1360 [M + Na]^+^ (calcd. for C_15_H_20_NaO_4_, 255.1361), 487.2818 [2M + Na]^+^ (calcd. for C_30_H_40_NaO_8_, 487.2824).

#### 3.3.5. Dihydrosporogen AO-1 (**5**)

Colorless oil; [α] + 108.0 (*c* 0.1, MeOH); UV (MeOH) *λ*_max_ (logε) 202 (3.89) nm; CD (0.3 mM, MeCN) *λ*_max_ (Δ*ε*) 198 (+7.6) nm; ^1^H NMR (400 MHz, Chloroform-*d*) *δ* 5.25 (1H, t, *J* = 2.3, 4.5 Hz, H-9), 5.17 (1H, br, H-12), 5.06 (1H, m, H-12), 4.52 (1H, t, *J* = 3.0, 5.7 Hz, H-8), 3.52 (1H, td, *J* = 4.5, 11.0 Hz, H-3), 3.08 (1H, s, H-6), 2.63 (1H, tdd, *J* = 2.0, 5.0, 14.4 Hz, H-1β), 2.17 (1H, ddd, *J* = 2.7, 4.7, 14.2 Hz, H-1α), 2.04 (1H, m, H-2β), 1.87 (3H, s, H_3_-13), 1.62 (1H, m, H-4), 1.33 (1H, m, H-2α), 1.01 (3H, s, H_3_-14), 1.16 (3H, d, *J* = 6.7 Hz, H_3_-15); ^13^C NMR (100 MHz, Chloroform-*d*) *δ* 65.5 (C-8), 140.5 (C-10), 142.2 (C-11), 119.6 (C-9), 113.5 (C-12), 72.0 (C-3), 68.0 (C-6), 65.7 (C-7), 43.8 (C-4), 38.9 (C-5), 35.9 (C-2), 30.3 (C-1), 19.8 (C-13), 17.8 (C-14), 11.4 (C-15); HRESIMS *m*/*z* 273.1467 [M + Na]^+^ (calcd. for C_15_H_22_NaO_3_, 273.1467), 523.3037 [2M + Na]^+^ (calcd. for C_30_H_44_NaO_6_, 523.3036).

#### 3.3.6. Sporogen AO-1 (**6**)

Colorless oil; [α] + 320.0 (*c* 0.1, MeOH); UV (MeOH) *λ*_max_ (log*ε*) 240 (4.02) nm; CD (0.3 mM, MeCN) *λ*_max_ (Δ*ε*) 219 (−6.1), 244 (+6.3), 335 (+1.7) nm; ^1^H NMR (400 MHz, Methanol-*d*_4_) *δ* 5.74 (1H, d, *J* = 2.0 Hz, H-9), 5.09 (1H, br, H-12), 5.07 (1H, t, *J* = 1.7, 3.5 Hz, H-12), 3.60 (1H, td, *J* = 4.3, 11.0 Hz, H-3), 3.42 (1H, s, H-6), 2.63 (1H, tdd, *J* = 2.0, 5.0, 14.4 Hz, H-1β), 2.36 (1H, tdd, *J* = 3.0, 4.4, 14.3 Hz, H-1α), 2.14 (1H, m, H-2β), 1.85 (3H, s, H_3_-13), 1.74 (1H, m, H-4), 1.35 (1H, m, H-2α), 1.28 (3H, s, H_3_-14), 1.26 (3H, d, *J* = 6.8 Hz, H3-15); ^13^C NMR (100 MHz, Methanol-*d*4) *δ* 194.8 (C-8), 166.8 (C-10), 141.0 (C-11), 121.4 (C-9), 114.3 (C-12), 71.3 (C-3), 70.0 (C-6), 64.5 (C-7), 45.7 (C-4), 42.4 (C-5), 36.4 (C-2), 32.0 (C-1), 19.9 (C-13), 19.1 (C-14), 11.6 (C-15); HRESIMS *m*/*z* 271.1305 [M + Na]^+^ (calcd. for C_15_H_20_NaO_3_, 271.1310).

#### 3.3.7. Phomenone (**7**)

Colorless oil; [α] + 137.0 (*c* 0.1, MeOH); UV (MeOH) *λ*_max_ (log*ε*) 242 (4.23) nm; ECD (0.3 mM, MeCN) *λ*_max_ (Δ*ε*) 219 (−9.2), 246 (+10.0), 333 (+3.0) nm; ^1^H NMR (400 MHz, Methanol-*d*_4_) *δ* 5.74 (1H, d, *J* = 2.0 Hz, H-9), 5.23 (1H, m, H-12), 5.28 (1H, m, H-12), 3.61 (1H, td, *J* = 4.4, 11.0 Hz, H-3), 3.42 (1H, s, H-6), 2.63 (1H, tdd, *J* = 2.0, 5.0, 14.5 Hz, H-1β), 2.36 (1H, ddd, *J* = 3.0, 4.3, 14.3 Hz, H-1α), 2.13 (1H, m, H-2β), 4.31 (1H, br d, *J* = 13.7, H-13), 4.19 (1H, br d, *J* = 13.7, H-13), 1.74 (1H, m, H-4), 1.38 (1H, m, H-2α), 1.28 (3H, s, H_3_-14), 1.26 (3H, d, *J* = 6.7 Hz, H_3_-15); ^13^C NMR (100 MHz, Methanol-*d*4) *δ* 194.7 (C-8), 166.8 (C-10), 145.9 (C-11), 121.3 (C-9), 113.3 (C-12), 71.2 (C-3), 70.4 (C-6), 62.8 (C-7), 45.7 (C-4), 42.6 (C-5), 36.4 (C-2), 32.0 (C-1), 64.0 (C-13), 18.7 (C-14), 11.6 (C-15); HRESIMS *m*/*z* 287.1260 [M + Na]^+^ (calcd. for C1_5_H_20_NaO_4_, 287.1259), 551.2617 [2M + Na]^+^ (calcd. for C_30_H_40_NaO_8_, 551.2621).

#### 3.3.8. Petasol (**8**)

Colorless oil; [α] + 181.0 (*c* 0.1, MeOH); UV (MeOH) *λ*_max_ (log*ε*) 236 (4.12) nm; CD (0.3 mM, MeCN) *λ*_max_ (Δ*ε*) 195 (+7.2), 232 (+7.9) nm; ^1^H NMR (400 MHz, Chloroform-*d*) *δ* 5.78 (1H, d, *J* = 2.0 Hz, H-9), 4.82 (1H, m, H-12), 4.99 (1H, m, H-12), 3.62 (1H, td, *J* = 4.4, 11.0 Hz, H-3), 2.02 (1H, dd, *J* = 4.6 Hz, H-6), 1.87 (1H, m, H-6), 2.45 (1H, tdd, *J* = 2.0, 5.0, 14.8 Hz, H-1β), 2.34 (1H, ddd, *J* = 3.0, 4.8, 14.9 Hz, H-1α), 2.16 (1H, m, H-2β), 1.74 (3H, s, H_3_-13), 1.34 (1H, m, H-4), 1.45 (1H, m, H-2α), 1.18 (3H, s, H_3_-14), 1.08 (3H, d, *J* = 6.7 Hz, H_3_-15); ^13^C NMR (100 MHz, Chloroform-*d*) *δ* 198.9 (C-8), 167.8 (C-10), 143.6 (C-11), 124.6 (C-9), 114.5 (C-12), 71.3 (C-3), 41.9 (C-6), 50.4 (C-7), 50.5 (C-4), 39.9 (C-5), 35.3 (C-2), 31.1 (C-1), 20.2 (C-13), 17.4 (C-14), 10.6 (C-15); HRESIMS *m*/*z* 273.1461 [M + Na]^+^ (calcd. for C_15_H_22_NaO_2_, 273.1467).

#### 3.3.9. 6-Dehydropetasol (**9**)

Colorless oil; [α]—9.0 (*c* 0.1, MeOH); UV (MeOH) *λ*_max_ (log*ε*) 240 (4.10) nm; CD (0.3 mM, MeCN) *λ*_max_ (Δ*ε*) 205 (−6.6), 227 (+3.0) nm; ^1^H NMR (400 MHz, Methanol-*d*_4_) *δ* 7.06 (1H, s, H-6), 6.05 (1H, d, *J* = 1.5 Hz, H-9), 5.14 (1H, d, *J* = 2.0 Hz, H-12), 5.06 (1H, t, *J* = 1.7, 3.5 Hz, H-12), 3.66 (1H, ddd, *J* = 4.6, 9.7, 14.5 Hz, H-3), 2.62 (1H, tdd, *J* = 2.0, 5.0, 14.1 Hz, H-1β), 2.41 (1H, ddd, *J* = 3.0, 4.6, 13.7 Hz, H-1α), 2.25 (1H, m, H-2β), 1.96 (3H, s, H_3_-13), 1.31 (1H, m, H-4), 1.39 (1H, m, H-2α), 1.22 (3H, s, H_3_-14), 1.26 (3H, d, *J* = 6.2 Hz, H_3_-15); ^13^C NMR (100 MHz, Methanol-*d*_4_) *δ* 187.3 (C-8), 169.8 (C-10), 143.3 (C-11), 125.0 (C-9), 116.4 (C-12), 71.5 (C-3), 153.5 (C-6), 140.2 (C-7), 49.9 (C-4), 44.7 (C-5), 37.5 (C-2), 31.5 (C-1), 22.6 (C-13), 19.0 (C-14), 12.2 (C-15); HRESIMS *m*/*z* 255.1360 [M + Na]^+^ (calcd. for C_15_H_20_NaO_2_, 255.1361), 487.2818 [2M + Na]^+^ (calcd. for C_30_H_40_NaO_4_, 487.2824).

### 3.4. Quantum Chemical Calculation

Initial conformational search of possible configurations with relative lower energy (within 10 kJ/mol) was conducted with the Crest program [44]. The Gaussian 16 package [45] was used for geometry optimizations, frequency analysis, and ECD calculation. The obtained conformers were optimized with density functional theory (DFT) at the B3LYP/6-31G(d) level and the conformers with populations over 1% were kept. Frequency analysis was performed on the same level to exclude the imaginary conformers. ECD spectra were calculated by an IEFPCM solvent model in acetonitrile with the time-dependent DFT (TDDFT) methodology at the B3LYP/TZVP level. SpecDis software, Version 1.71 [46] was applied to simulate the ECD spectra according to Boltzmann distributions of the lowest energy conformers.

### 3.5. Cell Culture

BV2 microglia cells were maintained in DMEM (Gibco, Waltham, MA, USA) with the addition of 10% fetal bovine serum and 1% streptomycin and penicillin. Cells were passaged every two days.

### 3.6. LPS Induction and Cellular NO Detection

The cellular NO levels were determined using a Nitric Oxide Assay Kit with DAF-FM DA (Beyotime, Nantong, China). A total of 5000 cells were planted in 96-well plates and maintained for 24 h. Then, they were induced with 1 μg/mL lipopolysaccharide (LPS) in the presence or absence of compounds at a final concentration of 20 μM for 24 h. Untreated wells were used as control. Then, supernatant of the culture medium was investigated with a Nitric Oxide Assay Kit with DAF-FM DA (Beyotime, S0020S) per the manufacturer’s instruction. Cells were stained with 2 µM hochest 33,342 at room temperature for 10 min, then cell numbers were auto-counted using the high-content screening system. The cell number in the LPS control group was set as 100%, and the relative cell survival ratios were calculated.

### 3.7. Cell Viability Assay

BV2 microglial cells were seeded into 96-well plates at a density of 1 × 10^4^ cells/well and allowed to adhere for 24 h. Cells were then exposed to different concentrations (20 μM for **1**–**9**; 0, 1, 5, 10, 20, 50, 100 μM for **5**) of the test compounds (**1**–**9**) in triplicate, with DMSO concentrations normalized across all treatment groups. After 24 h incubation, 10 μL CCK-8 reagent (Beyotime Biotechnology, Nantong, China) was added to each well, followed by 30 min incubation at 37 °C. Absorbance at 450 nm was quantified using a BioTek microplate reader (BioTek, Winooski, VT, USA), with cell viability calculated relative to vehicle-treated controls.

### 3.8. Western Blot Assay

Cells or tissues were lysed with RIPA lysis buffer containing protease and phosphatase inhibitor cocktail and total protein was extracted and quantified by using a protein determination kit (Thermo Fisher Scientific, Cat#23227, Beijing, China). Protein samples were prepared by centrifugation at the speed of 12,000× *g* for 30 min at 4 °C; the supernatant was boiled at 95 °C for 10 min and then subjected to SDS-PAGE. An amount of 15 µg of total protein was separated by electrophoresis in a 10% SDS-polyacrylamide gel and transferred to a PVDF membrane (Thermo Fisher Scientific, Cat#88520, Beijing, China). After blocking, the full membranes were cropped according to the molecular weight of examined proteins and incubated with antibodies at 4 °C overnight. The used antibodies were listed as follows: anti-iNOS (Abcam, ab178945, Cambridge, UK), anti-COX2 (Affinity, AF7003, Changzhou, China), anti-actin (Affinity Biosciences, AF7018, Changzhou, China), anti-ARG1 (Proteintech, 66129, Wuhan, China), anti-IL-1β (Proteintech, 26048, Wuhan, China), anti-GAPDH (Bioss, bs-41373R, Beijing, China), anti-IL-6 (Affinity, DF6087, Changzhou, China), anti-TNFα (Affinity, AF7014, Changzhou, China), anti-IBA1 (Affinity, DF6442, Changzhou, China). The membranes were washed to remove unbound antibodies and then incubated with HRP-conjugated secondary antibodies at room temperature for 45 min. Protein bands were visualized one by one with an ECL kit (Millipore, Cat#6-201BP, Beijing, China). The uncropped immunoblot data were uploaded as a Supplementary File. Densitometry analysis was performed using Image J Software, Version 1.54p (Rawak Software Inc., Stuttgart, Germany) relative to the loading control, β-actin; GAPDH; or tubulin. The average protein levels of control group cells or control mice from each independent experiment or mice were set as 1, and relative changes were calculated.

### 3.9. Immunofluorescence

For the immunofluorescence assay, cells were fixed with pre-cold ethanol at –20 °C, then blocked and incubated with anti-iNOS (Abcam, ab178945) and mouse anti-ARG 1 (Proteintech, 66129) at 4 °C overnight. After washing, cells were co-stained with fluorescent labeled secondary antibody (Cell Signaling Technology, Cat#7076, Danvers, MA, USA) and 2 μg/mL DAPI (for nucleus, Thermo Fisher Scientific, Cat#R37606, China) at 37 °C for 1 h. Fluorescence images were acquired using a confocal microscope (Olympus, Tokyo, Japan).

### 3.10. LPS-Induced Neuroinflammation Models

Male, 4-week-old C57BL/6J mice were obtained from Peking SiPeiFu Biotechnology Co., Ltd. (Beijing, China), with quality certificate number No.110324241107423512. After acclimation, mice were weighted and divided randomly into 6 groups, including control, LPS control mice, LPS + dexamethasone (Dex, 15 mg/kg), and LPS + HN-005 (10, 20, 40 mg/kg). Mice were intraperitoneally injected with dexamethasone or compound **5** for 2 days, while the control and LPS control group was intraperitoneally injected with an identical volume of 200 μL 0.9% saline. A total of 6 mg/kg LPS was intraperitoneally injected on the second day beyond the control group. After treatment, the mice were weighed and euthanized for brain anatomy.

### 3.11. Perfusion and Immunochemistry

Mice were anesthetized and their chest dissected to expose the heart sufficiently. The infusion needle penetrated the ventriculus sinister by about 0.5 cm and perfused it with 40 mL sterile PBS buffer until the liver turned white. Then, the prefrontal cortex and hippocampus were separated and preserved. For the immunochemistry assay, the cortex and hippocampus were fixed at 4% PFA at room temperature for 2–4 days. The fixed brain was embedded into paraffin and sectioned at 5 μm in the coronal plane using a sliding microtone (Leica, CM1950, Weztlar, Germany), then blocked with 5% goat serum TBS buffer at room temperature for one hour. After blocking, tissue slides were incubated with the first antibody against IBA1 (Affinity, DF6442, Changzhou, China) at 4 °C for 16 h, then rinsed with TBST three times and incubated with Alex flour 555 conjugated goat anti-rabbit secondary antibody (1:500) and DAPI (1:1000) at 4 °C overnight. A section incubated only with the secondary antibody was used as a negative control. For HE staining, the section was stained with an H&E staining kit (Servicebio, G1076, Wuhan, China) per the manufacturer’s instruction. Fluorescence images were acquired using a confocal microscope (Olympus, Tokyo, Japan).

### 3.12. mRNA Level of Inflammation Factor Detection with qPCR

Total RNA from cells or tissues of the cortex and hippocampus was isolated using the TRIzol method (Invitrogen, Cat# 15596018, Guangzhou, China), and the results were analyzed on an ABI StepOne Plus real-time PCR system (Applied Biosystems, Foster, CA, USA, RRID:SCR_015805) using the 2^−ΔΔCt^ method as in our previous reports [47,48]. β-actin was used as a loading control, and relative mRNA levels were normalized to loading control β-actin. The primers included the following: GAPDH-F-AGGTCGGTGTGAACGGATTTG, GAPDH-R-TGTAGACCATGTAGTTGAGGTCA; iNOS-F-GTTCTCAGCCCAACAATACAAGA, iNOS-R-GTGGACGGGTCGATGTCAC; COX2-F-TGAGTACCGCAAACGCTTCT, COX2-R-CAGCCATTTCCTTCTCTCCTGT; IL-1β-F-ATGCCACCTTTTGACAGTGAT, IL-1β-R-AAGGTCCACGGGAAAGACAC; IL-6-F-TAGTCCTTCCTACCCCAATTTCC, IL-6-R-TTGGTCCTTAGCCACTCCTTC. The average gene levels in control cells or control mice were set as 1, and relative folds were calculated by comparing them with the control group.

## 4. Conclusions

Three new eremophilane sesquiterpenoids (**1**–**3**) together with six known analogues (**4**–**9**) were obtained from marine-derived fungus *Phoma* sp. DXH009. Their anti-neuroinflammatory activities were evaluated in the lipopolysaccharide (LPS)-induced BV2 microglial cells. Compound **5** exhibited significant inhibitory effects on the production of NO levels (EC_50_ = 3.11 μM) with less cytotoxicity. The structure–activity relationship (SAR) of compounds **1**–**9** was fully discussed, and it offers preliminary guidance for further optimization of 3-*hydroxy*-9,10,-*ene*-eremophilanes, though further mechanistic validation is required to confirm these hypotheses. Further mechanistic studies suggested that **5** inhibited microphage depolarization alongside NF-κB signaling suppression in LPS-treated BV2 microglial cells. Correspondingly, **5** efficiently attenuated neuroinflammation in the cortex and hippocampus of LPS-treated mice. This work expands the membership of the eremophilane sesquiterpenoid family, and highlights the potential of **5** for the development of anti-neuroinflammatory drugs.

## Figures and Tables

**Table 1:** The 1D NMR data for compounds **1**–**4** (*δ* in ppm, *J* in Hz).

No.	1 ^a^		2 ^b^		3 ^b^		4 ^a^	
	*δ*_C_, Type	*δ*_H_	*δ*_C_, Type	*δ*_H_	*δ*_C_, Type	*δ*_H_	*δ*_C_, Type	*δ*_H_
1	38.9, CH_2_	2.53, m 2.60, m	31.4, CH_2_	α 2.42, ddd (13.7, 4.6, 2.7) β 2.60, tdd (13.9, 5.0, 1.5)	32.3, CH_2_	α 2.36, ddd (14.0, 4.5, 2.7) β 2.61, tdd (14.4, 5.2, 1.9)	74.0, CH	4.48, t (2.8)
2	74.1, CH	3.48, m	37.5, CH_2_	1.38, m 2.24, m	37.0, CH_2_	1.39, dt (11.7, 4.0) 2.17, m	41.7, CH_2_	1.65, m 2.32, m
3	76.2, CH	3.42, t (9.0)	71.5, CH	3.67, m	71.8, CH	3.58, td (10.9, 4.7)	67.1, CH	4.03, td (11.0, 4.0)
4	40.5, CH	1.87, br s	48.6, CH	1.32, m	49.9, CH	1.50, m	44.3, CH	1.80, m
5	41.1, C		44.5, C		41.3, C		40.9, C	
6	68.0, CH	3.32, s	151.6, CH	7.31, s	45.9, CH_2_	α 2.20, d (14.8) β 1.87, d (14.7)	68.6, CH	3.34, s
7	63.6, C		143.1, C		78.2, C		64.1, C	
8	192.7, C		188.2, C		200.6, C		194.2, C	
9	123.0, CH	5.81, d (1.6)	125.6, CH	6.04, d (1.5)	123.1, CH	5.86, d (1.7)	123.8, CH	5.86, s
10	159.6, C		169.9, C		172.3, C		160.6, C	
11	139.0, C		72.4, C		148.8, C		138.7, C	
12	114.8, CH_2_	5.11, br s	29.1, CH_3_	1.46, s	113.5, CH_2_	5.00, d (1.9) 5.04, s	115.0, CH_2_	5.12, m
13	19.9, CH_3_	1.87, br s	29.1, CH_3_	1.47, s	19.4, CH_3_	1.74, s	19.9, CH_3_	1.85, s
14	19.0, CH_3_	1.24, s	19.0, CH_3_	1.20, s	22.3, CH_3_	1.27, s	20.8, CH_3_	1.40, s
15	11.2, CH_3_	1.27, d (6.8)	12.2, CH_3_	1.28, d (2.3)	11.7, CH_3_	1.10, d (6.7)	11.3, CH_3_	1.27, d (6.7)

^a^ Data were recorded in CDCl_3_. ^b^ Data were recorded in CD_3_OD.

## Data Availability

Data are contained within the article or Supplementary Materials.

## Supplementary Materials

The following supporting information can be downloaded at https://www.mdpi.com/article/10.3390/md23030094/s1. Figure S1: ^1^H NMR spectrum (CDCl_3_) of **1**; Figure S2: ^13^C NMR spectrum (CDCl_3_) of **1**; Figure S3: HSQC spectrum (CDCl_3_) of **1**; Figure S4: ^1^H–^1^H COSY spectrum (CDCl_3_) of **1**; Figure S5: HMBC spectrum (CDCl_3_) of **1**; Figure S6: NOESY spectrum (CDCl_3_) of **1**; Figure S7: NOESY spectrum (CD_3_OD) of **1**; Figure S8: HRESIMS spectrum of **1**; Figure S9: UV spectrum of **1**; Table S1: Conformational analysis of the optimized isomers of **1** in methanol; Table S2: The coordinates of the optimized conformers of **1**; Figure S10: ^1^H NMR spectrum (CD_3_OD) of **2**; Figure S11: ^13^C NMR spectrum (CD_3_OD) of **2**; Figure S12: HSQC spectrum (CD_3_OD) of **2**; Figure S13: ^1^H–^1^H COSY spectrum (CD_3_OD) of **2**; Figure S14: HMBC spectrum (CD_3_OD) of **2**; Figure S15: NOESY spectrum (CD_3_OD) of **2**; Figure S16: HRESIMS spectrum of **2**; Figure S17: UV spectrum of **2**; Table S3: Conformational analysis of the optimized isomers of **2** in methanol; Table S4: The coordinates of the optimized conformers of **2**; Figure S18: ^1^H NMR spectrum (CD_3_OD) of **3**; Figure S19: ^13^C NMR spectrum (CD_3_OD) of **3**; Figure S20: ^1^H–^1^H COSY spectrum (CD_3_OD) of **3**; Figure S21: HMBC spectrum (CD_3_OD) of **3**; Figure S22: NOESY spectrum (CD_3_OD) of **3**; Figure S23: HRESIMS spectrum of **3**; Figure S24: UV spectrum of **3**; Table S5: Conformational analysis of the optimized isomers of **3** in methanol; Table S6: The coordinates of the optimized conformers of **3**; Figure S25: ^1^H NMR spectrum (CDCl_3_) of **4**; Figure S26: ^13^C NMR spectrum (CDCl_3_) of **4**; Figure S27: NOESY spectrum (CDCl_3_) of **4**; Figure S28: HRESIMS spectrum of **4**; Figure S29: UV spectrum of **4**; Table S7: Conformational analysis of the optimized isomers of **4** in methanol; Table S8: The coordinates of the optimized conformers of **4**; Figure S30: ^1^H NMR spectrum (CDCl_3_) of **5**; Figure S31: ^13^C NMR spectrum (CDCl_3_) of **5**; Figure S32: HRESIMS spectrum of **5**; Figure S33: ^1^H NMR spectrum (CD_3_OD) of **6**; Figure S34: ^13^C NMR spectrum (CD_3_OD) of **6**; Figure S35: HRESIMS spectrum of **6**; Figure S36: ^1^H NMR spectrum (CD_3_OD) of **7**; Figure S37: ^13^C NMR spectrum (CD_3_OD) of **7**; Figure S38: HRESIMS spectrum of **7**; Figure S39: ^1^H NMR spectrum (CDCl_3_) of **8**; Figure S40: ^13^C NMR spectrum (CDCl_3_) of **8**; Figure S41: HRESIMS spectrum of **8**; Figure S42: ^1^H NMR spectrum (CDCl_3_) of **9**; Figure S43: ^13^C NMR spectrum (CDCl_3_) of **9**; Figure S44: HRESIMS spectrum of **9**.
