# Supplementary material for: Anti-Neuroinflammatory Eremophilane Sesquiterpenoids from Marine-Derived Fungus Phoma sp. DXH009
Source: Mar Drugs. 2025 Feb 20;23(3):94. doi: 10.3390/md23030094 (PMC11943946; doi:10.3390/md23030094)
Supplement: Supplementary file 1 [file marinedrugs-23-00094-s001.zip › marinedrugs-3464170-supplementary.pdf]

Anti-neuroinflammatory eremophilane sesquiterpenoids from marine-derived fungus *Phoma* sp. DXH009

Guanyu Yang †, Mengwei Qin †, Mingbin Chen †, Yujia Shi, Siyi Liu, Yong Rao, Ling Huang * and Ying Fu *

Key Laboratory of Tropical Biological Resources, Ministry of Education; School of Pharmaceutical Sciences; Hainan University, Haikou 570228, China; yangguanyu@hainanu.edu.cn (G. Yang); 22211007000035@hainanu.edu.cn (M. Qin); 22211007000020@hainanu.edu.cn (M. Chen); 23221055000018@hainanu.edu.cn (Y. Shi); lsy@hainanu.edu.cn (S. Liu); raoyong@hainanu.edu.cn (Y. Rao).

* Correspondence: linghuang@hainanu.edu.cn (L. Huang); fuying@hainanu.edu.cn (Y. Fu).

† These authors contributed equally to this work.

Contentes

Figure S1. ^{1}H NMR spectrum ($CDCl_3$) of 1. 3

Figure S2. ^{13}C NMR spectrum ($CDCl_3$) of 1. 3

Figure S3. HSQC spectrum ($CDCl_3$) of 1. 4

Figure S4. ^{1}H–^{1}H COSY spectrum ($CDCl_3$) of 1. 4

Figure S5. HMBC spectrum ($CDCl_3$) of 1. 5

Figure S6. NOESY spectrum ($CDCl_3$) of 1. 5

Figure S7. NOESY spectrum (CD_3OD) of 1. 6

Figure S8. HRESIMS spectrum of 1. 6

Figure S9. UV spectrum of 1. 7

Table S1. Conformational analysis of the optimized isomers of 1 in methanol. 7

Table S2. The coordinates of the optimized conformers of 1. 8

Figure S10. ^{1}H NMR spectrum (CD_3OD) of 2. 9

Figure S11. ^{13}C NMR spectrum (CD_3OD) of 2. 9

Figure S12. HSQC spectrum (CD_3OD) of 2. 10

Figure S13. ^{1}H–^{1}H COSY spectrum (CD_3OD) of 2. 10

Figure S14. HMBC spectrum (CD_3OD) of 2. 11

Figure S15. NOESY spectrum (CD_3OD) of 2. 11

Figure S16. HRESIMS spectrum of 2. 12

Figure S17. UV spectrum of 2. 12

Table S3. Conformational analysis of the optimized isomers of 2 in methanol. 13

Table S4. The coordinates of the optimized conformers of 2. 14

Figure S18. ^{1}H NMR spectrum (CD_3OD) of 3. 15

Figure S19. ^{13}C NMR spectrum (CD_3OD) of 3. 16

Figure S20. ^{1}H–^{1}H COSY spectrum (CD_3OD) of 3. 16

Figure S21. HMBC spectrum (CD_3OD) of 3. 17

Figure S22. NOESY spectrum (CD_3OD) of 3. 17

Figure S23. HRESIMS spectrum of 3. 18

Figure S24. UV spectrum of 3. **Error! Bookmark not defined.**

Table S5. Conformational analysis of the optimized isomers of 3 in methanol. 19

Table S6. The coordinates of the optimized conformers of 3. 19

Figure S25. ^{1}H NMR spectrum ($CDCl_3$) of 4. 20

Figure S26. ^{13}C NMR spectrum ($CDCl_3$) of 4. 21

Figure S27. NOESY spectrum ($CDCl_3$) of 4. 21

Figure S28. HRESIMS spectrum of 4. 22

Figure S29. UV spectrum of 4. **Error! Bookmark not defined.**

Table S7. Conformational analysis of the optimized isomers of 4 in methanol. 27

Table S8. The coordinates of the optimized conformers of 4. 28

Figure S30. ^{1}H NMR spectrum ($CDCl_3$) of 5. 24

Figure S31. ^{13}C NMR spectrum ($CDCl_3$) of 5. 25

Figure S32. HRESIMS spectrum of 5. 25

Figure S33. ^{1}H NMR spectrum (CD_3OD) of 6. 26

Figure S34. ^{13}C NMR spectrum (CD_3OD) of 6. 26

Figure S35. HRESIMS spectrum of 6. 27

Figure S36. ^{1}H NMR spectrum (CD_3OD) of 7. 27

Figure S37. ^{13}C NMR spectrum (CD_3OD) of 7. 28

Figure S38. HRESIMS spectrum of 7. 28

Figure S39. ^{1}H NMR spectrum ($CDCl_3$) of 8. 29

Figure S40. ^{13}C NMR spectrum ($CDCl_3$) of 8. 29

Figure S41. HRESIMS spectrum of 8. 30

Figure S42. ^{1}H NMR spectrum ($CDCl_3$) of 9. 30

Figure S43. ^{13}C NMR spectrum ($CDCl_3$) of 9. 31

Figure S44. HRESIMS spectrum of 9. 31

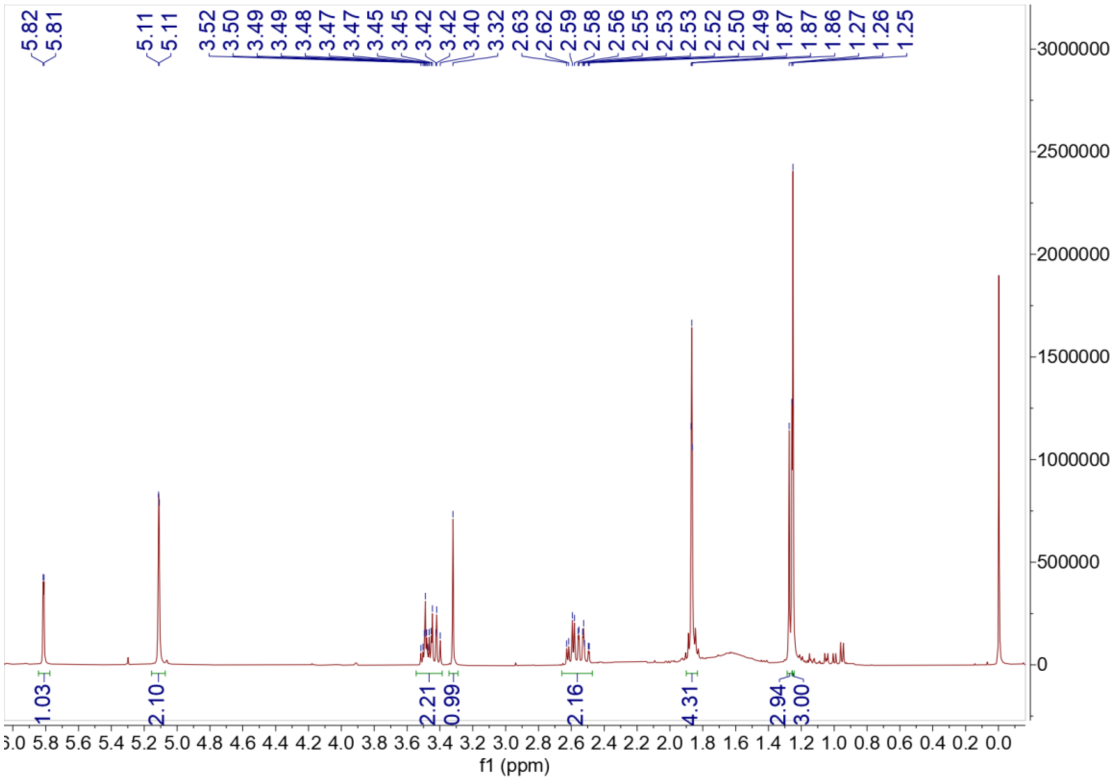

Figure S1. ^{1}H NMR spectrum ($CDCl_3$) of 1

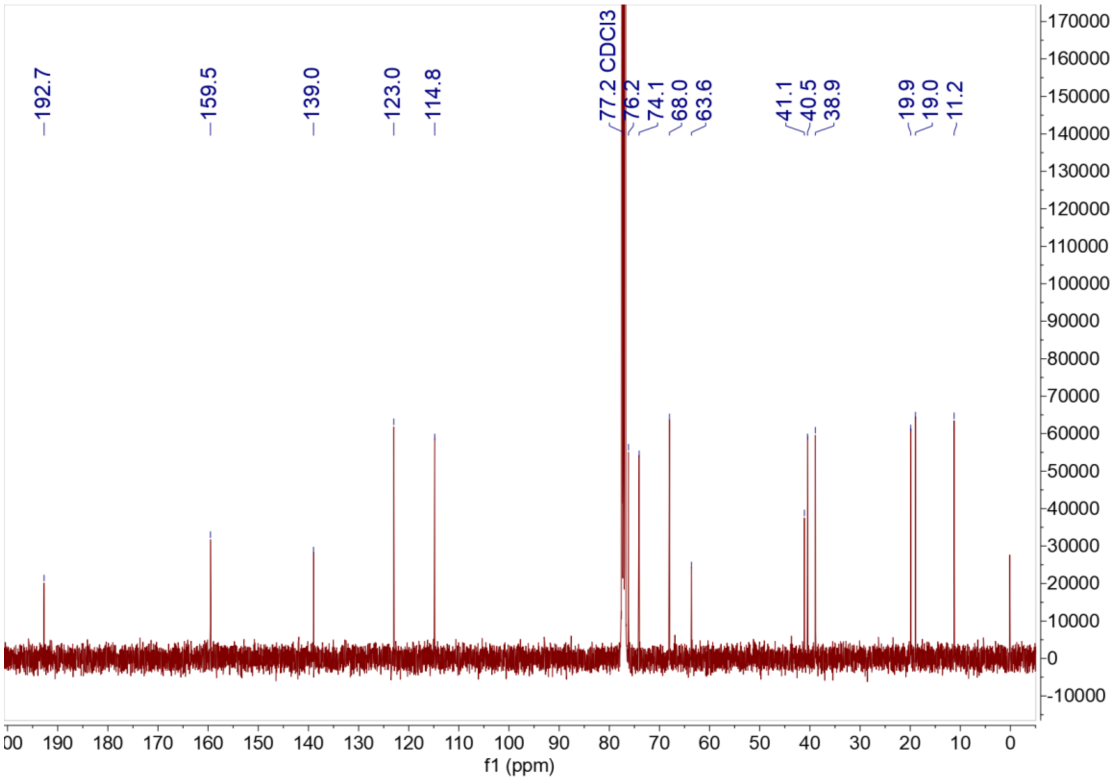

Figure S2. ^{13}C NMR spectrum ($CDCl_3$) of 1

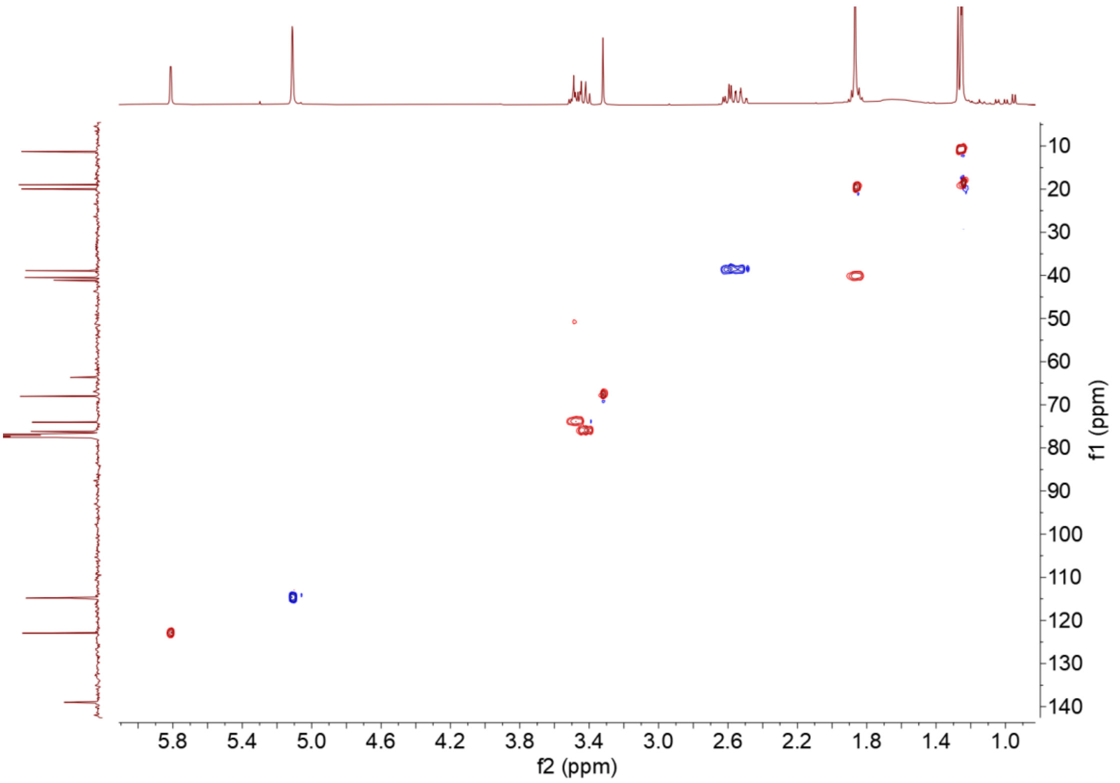

Figure S3. HSQC spectrum ($CDCl_3$) of 1

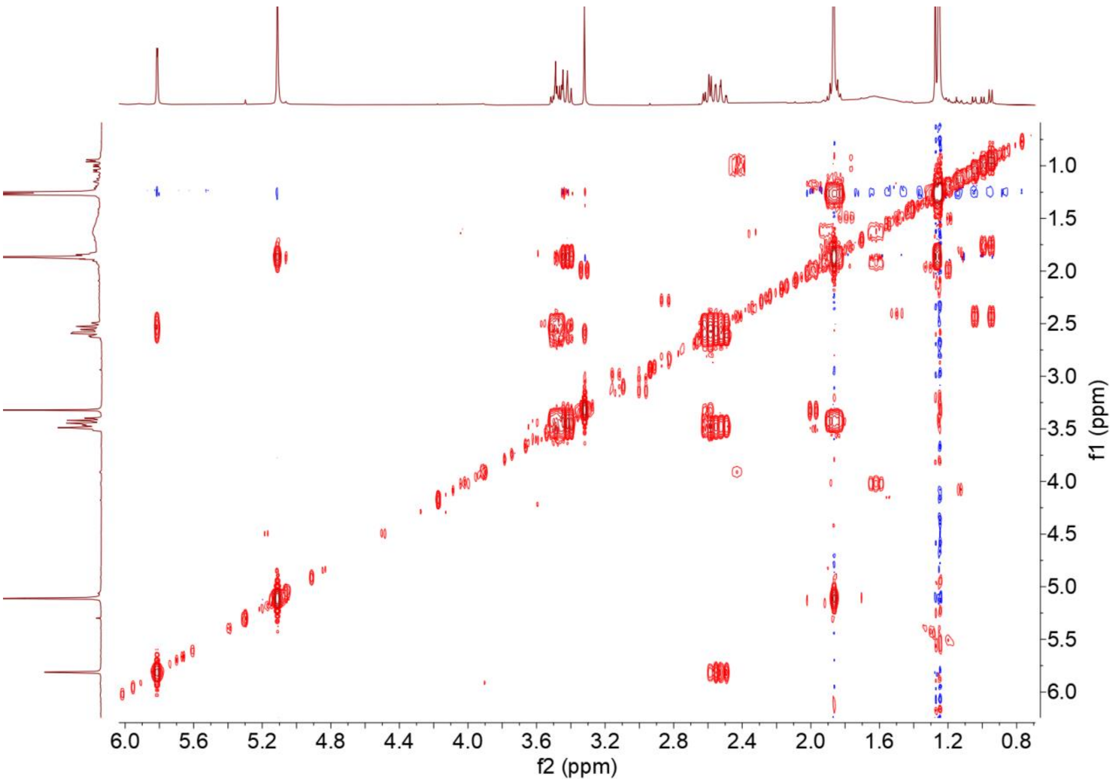

Figure S4. ^1H–^1H COSY spectrum ($CDCl_3$) of 1

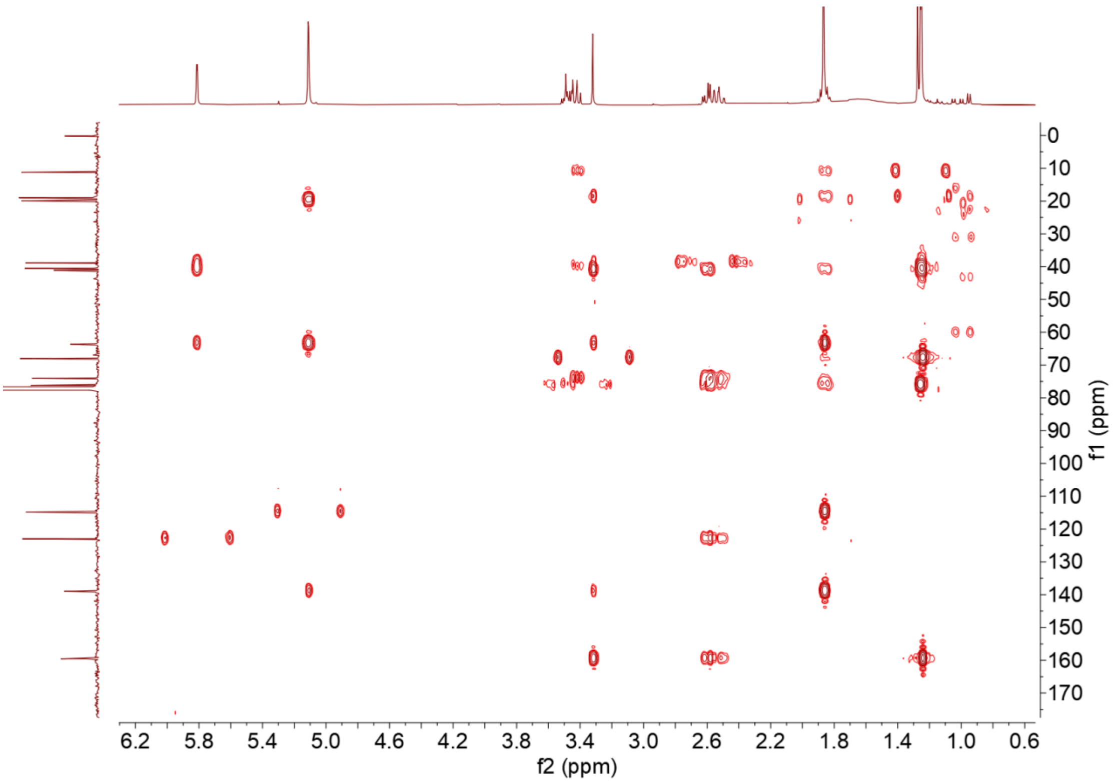

Figure S5. HMBC spectrum ($CDCl_3$) of 1

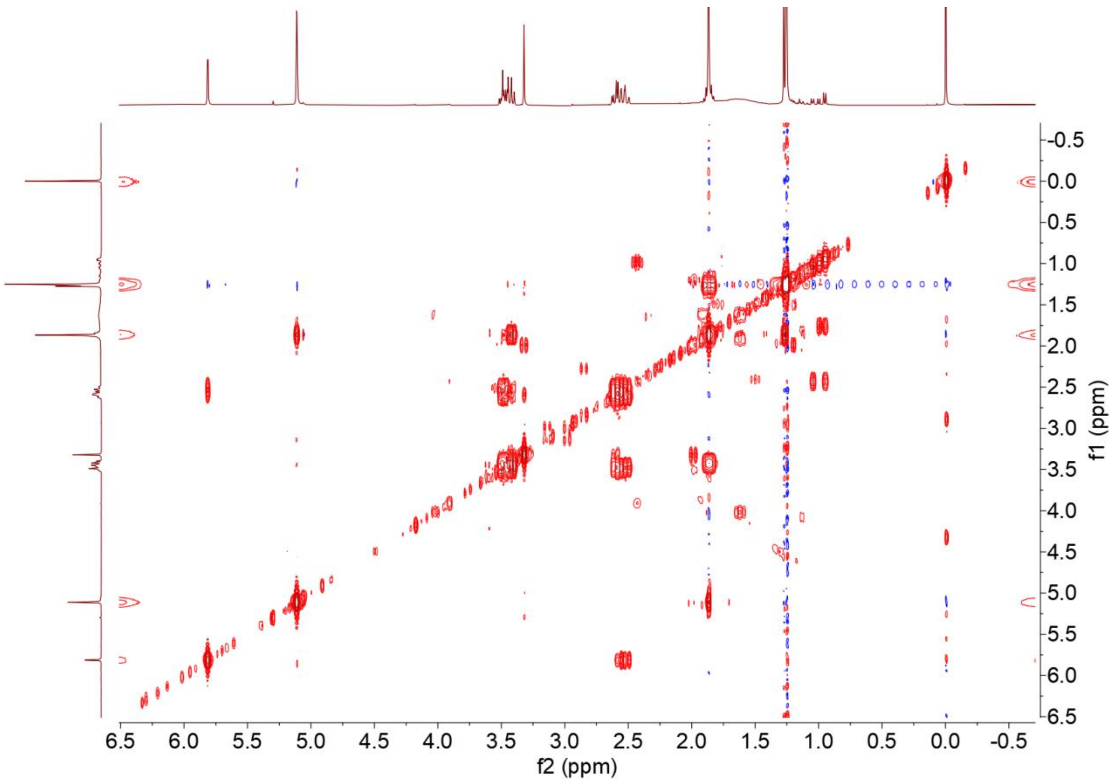

Figure S6. NOESY spectrum ($CDCl_3$) of 1

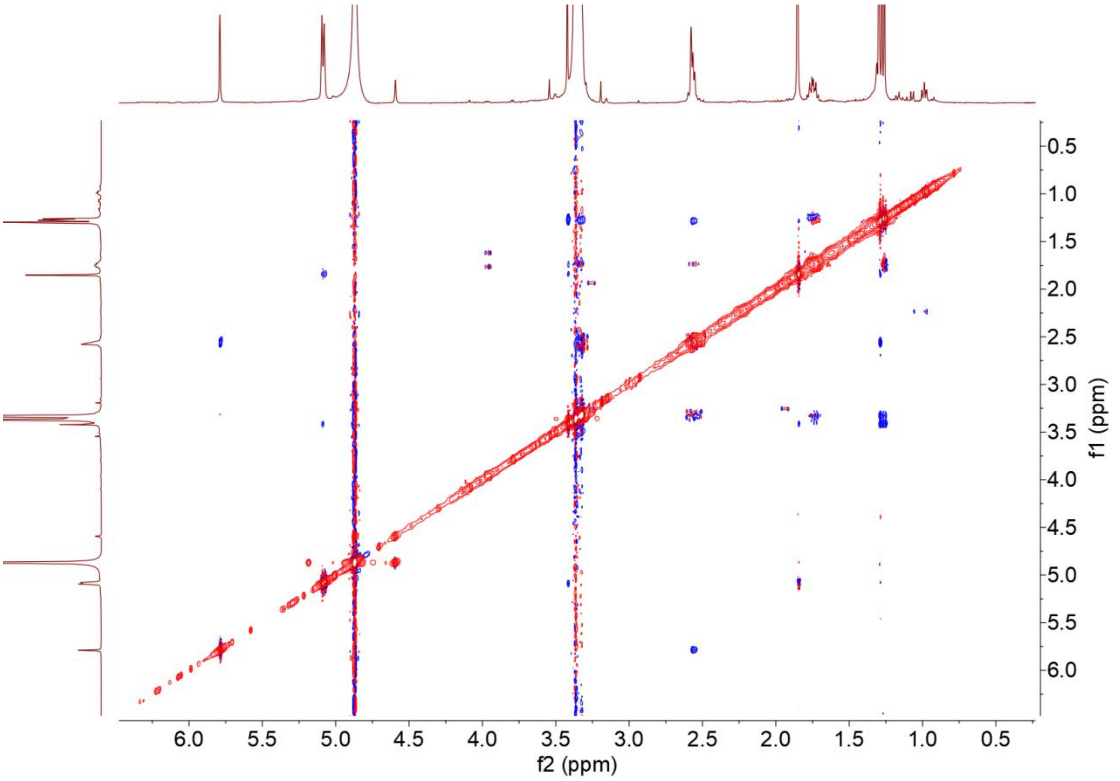

Figure S7. NOESY spectrum (CD_3OD) of 1

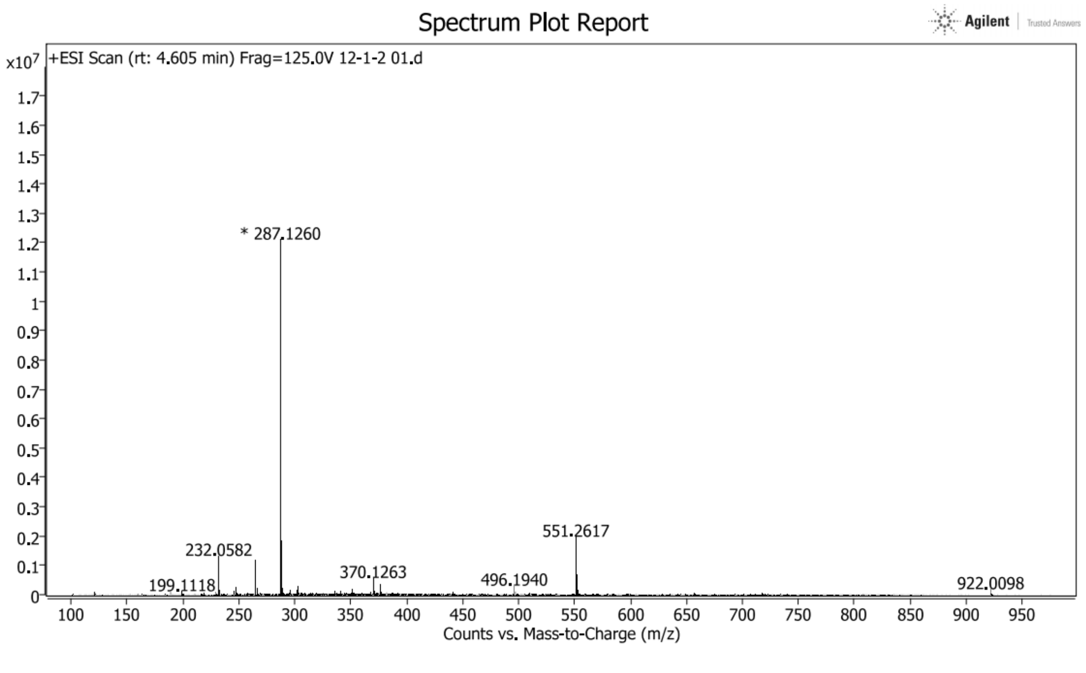

Figure S8. HRESIMS spectrum of 1

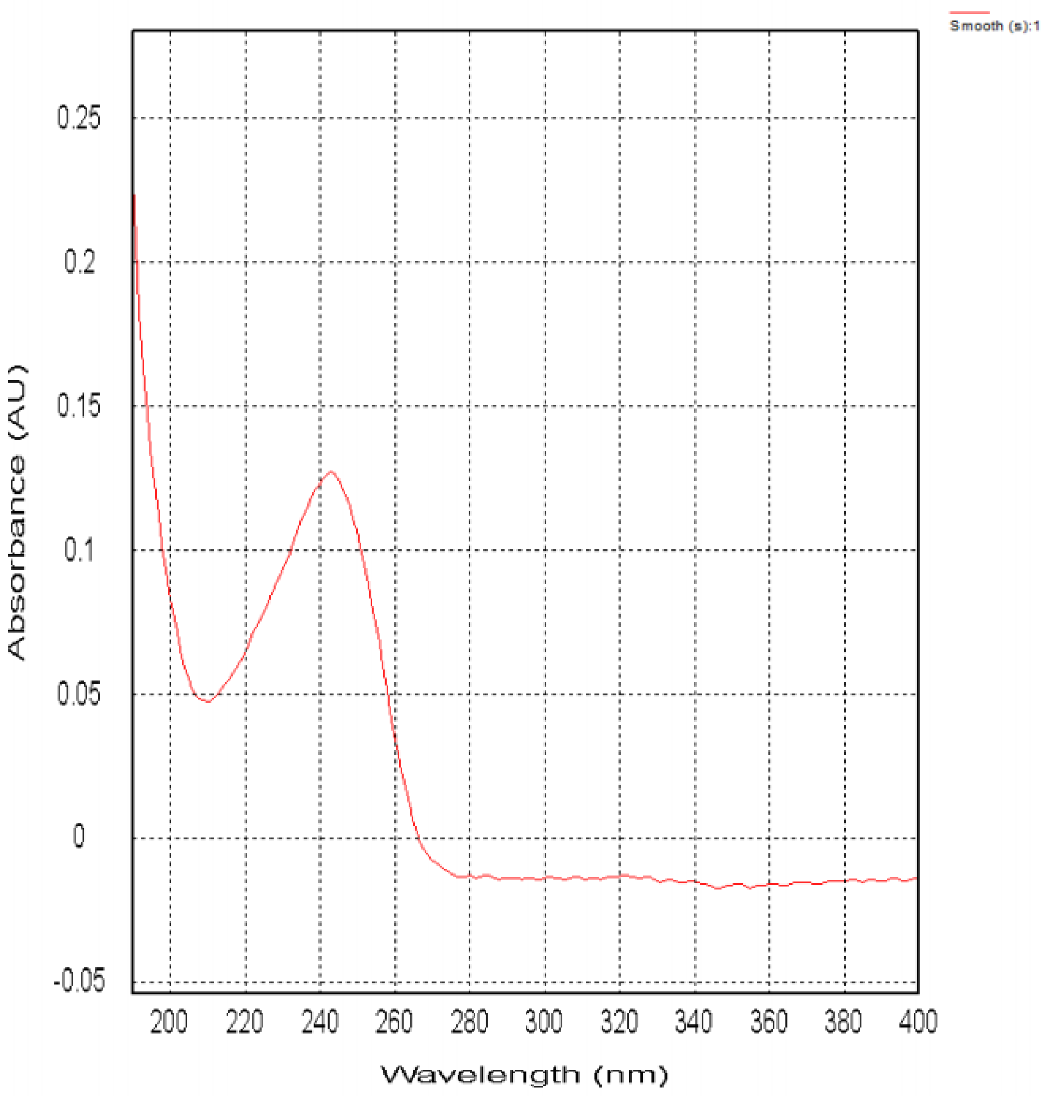

Figure S9. UV spectrum of compound 1

Table S1. Conformational analysis of the optimized isomers of 1 in methanol.

Conformations	G (hartree)	ΔG (kcal/mol)	Boltzmann distributions (%)
1-1	-884.47586	0	33.3
1-2	-884.475752	0.067771015	29.6
1-3	-884.47551	0.21962829	22.9
1-4	-884.474494	0.85717784	7.8
1-5	-884.474308	0.973894589	6.4

Table S2. The coordinates of the optimized conformers of 1.

	1-1			1-2			1-3			1-4			1-5		
C	-1.98462	1.60398	0.7113	-1.98265	1.60685	0.71673	-1.9909	1.59684	0.74723	-2.01267	-1.54063	-0.8426	-2.00174	1.55888	0.81279
C	-2.99275	1.05878	-0.30502	-2.99383	1.06639	-0.29999	-2.96231	1.09456	-0.32565	-2.93629	-1.15361	0.31582	-2.9733	1.12816	-0.29034
C	-2.99388	-0.47025	-0.27947	-2.99939	-0.45691	-0.28328	-2.9931	-0.43466	-0.32329	-2.98615	0.37501	0.44962	-3.00087	-0.39008	-0.40929
C	-1.59578	-1.04904	-0.51594	-1.59706	-1.04285	-0.5196	-1.59547	-1.04444	-0.52366	-1.59404	0.98329	0.64138	-1.59815	-0.9775	-0.63839
C	-0.48818	-0.45582	0.42763	-0.48979	-0.45382	0.42524	-0.49794	-0.46663	0.44341	-0.5326	0.51336	-0.42076	-0.52473	-0.49554	0.40084
C	-0.61154	1.05769	0.45126	-0.61069	1.05968	0.45145	-0.6174	1.04736	0.48551	-0.63534	-0.99181	-0.60256	-0.62653	1.01077	0.56589
C	0.85648	-0.90019	-0.11806	0.85496	-0.90112	-0.11748	0.84916	-0.90932	-0.09849	0.83116	0.92114	0.10857	0.83633	-0.91127	-0.12759
C	2.02285	-0.00314	-0.26395	2.02272	-0.0058	-0.26365	2.01471	-0.01211	-0.25483	2.01129	0.03699	0.11028	2.01971	-0.03218	-0.11921
C	1.80631	1.46049	0.04418	1.8076	1.45863	0.04238	1.79827	1.45142	0.04897	1.8078	-1.38228	-0.35986	1.81865	1.38859	0.35104
C	0.43043	1.88847	0.27129	0.43243	1.88871	0.27095	0.4206	1.87932	0.28514	0.42479	-1.81786	-0.5488	0.43894	1.82988	0.53109
C	3.40853	-0.54876	-0.14526	3.40771	-0.55291	-0.14379	3.40179	-0.55594	-0.14576	3.40139	0.58678	0.0668	3.40701	-0.58845	-0.06395
C	3.99064	-1.14846	-1.1814	3.98868	-1.15589	-1.17866	3.97484	-1.15803	-1.1855	3.75082	1.44075	-0.89339	3.7448	-1.44534	0.89789
C	4.04351	-0.41259	1.21159	4.04333	-0.41417	1.21249	4.04818	-0.41487	1.20515	4.32943	0.10477	1.1457	4.34666	-0.11002	-1.13437
O	2.74981	2.23911	0.08396	2.75189	2.23624	0.08019	2.7374	2.23335	0.08304	2.75785	-2.12051	-0.5778	2.77392	2.12079	0.5732
O	1.24435	-0.42668	-1.4085	1.24434	-0.42955	-1.40807	1.22725	-0.43682	-1.39265	1.26561	0.32001	1.33072	1.28429	-0.31417	-1.34602
C	-1.64732	-2.57889	-0.52749	-1.64989	-2.57337	-0.52731	-1.67046	-2.57372	-0.52171	-1.69158	2.50404	0.78789	-1.67382	-2.50009	-0.78709
C	-0.5701	-0.98	1.87988	-0.57921	-0.97728	1.87789	-0.60722	-1.00865	1.88675	-0.70515	1.18939	-1.79935	-0.68067	-1.14994	1.79363
O	-2.66897	1.43921	-1.6407	-2.663	1.44865	-1.63345	-2.62742	1.59269	-1.61976	-2.54086	-1.76341	1.54459	-2.57807	1.62184	-1.56859
O	-3.50257	-0.94031	0.97047	-3.58134	-0.83411	0.96736	-3.61053	-0.81413	0.91062	-3.59693	0.95112	-0.70853	-3.64083	-0.86479	0.77839
H	-1.97419	2.69659	0.6927	-1.9708	2.69954	0.69816	-1.97524	2.68784	0.75784	-1.98282	-2.62541	-0.95594	-1.97235	2.64879	0.88873
H	-2.32752	1.30205	1.70581	-2.32239	1.30696	1.71303	-2.35652	1.25624	1.7196	-2.43215	-1.11711	-1.75899	-2.39154	1.18163	1.76355
H	-3.99629	1.41985	-0.04351	-3.99699	1.42003	-0.03644	-3.96882	1.45899	-0.11352	-3.94556	-1.52492	0.11927	-3.98229	1.47497	-0.04011
H	-3.63752	-0.8187	-1.0967	-3.64213	-0.79604	-1.10522	-3.61974	-0.7567	-1.16389	-3.57825	0.61589	1.34114	-3.61285	-0.64456	-1.28367
H	-1.32111	-0.70902	-1.51828	-1.32251	-0.71118	-1.52512	-1.28861	-0.73499	-1.52867	-1.23665	0.5824	1.5957	-1.27577	-0.56171	-1.59741
H	1.09826	-1.9475	0.04143	1.09441	-1.94897	0.0431	1.0918	-1.95662	0.06017	1.04486	1.9842	0.05485	1.04442	-1.97547	-0.07044

H	0.28992	2.9633	0.33729	0.29402	2.96368	0.33879	0.28079	2.95381	0.35522	0.29853	-2.8817	-0.72579	0.31645	2.89447	0.70705
H	4.98369	-1.5777	-1.09923	4.98118	-1.58625	-1.09572	4.96902	-1.58593	-1.11094	4.75784	1.84059	-0.95667	4.74937	-1.85017	0.96886
H	3.48732	-1.23101	-2.13733	3.48501	-1.24003	-2.13426	3.4632	-1.24378	-2.1367	3.04663	1.76322	-1.65339	3.03292	-1.76532	1.6518
H	4.20547	0.63819	1.46184	4.20704	0.63701	1.45991	4.21091	0.63671	1.45167	4.46729	-0.97705	1.07423	5.31975	-0.5979	-1.05973
H	3.39502	-0.83137	1.98871	3.39457	-0.82991	1.99101	3.40761	-0.83303	1.98912	5.30563	0.58727	1.07793	3.92989	-0.31283	-2.1253
H	5.00319	-0.92986	1.2502	5.00222	-0.93281	1.25204	5.00902	-0.93038	1.23628	3.90595	0.31169	2.13292	4.48927	0.97091	-1.05961
H	-2.37107	-2.9141	-1.2751	-2.41065	-2.91358	-1.2351	-2.41243	-2.90787	-1.2518	-2.3654	2.74802	1.61322	-0.72157	-2.93213	-1.09752
H	-1.96141	-2.98011	0.43572	-1.8981	-2.98851	0.45196	-1.95843	-2.97327	0.45275	-2.0926	2.9686	-0.11214	-2.40888	-2.76071	-1.55342
H	-0.68505	-3.02163	-0.78972	-0.70279	-3.01648	-0.83801	-0.72018	-3.03378	-0.79593	-0.72621	2.95997	1.0132	-1.9697	-2.99709	0.13946
H	-0.41606	-2.06038	1.90422	-1.55842	-0.78516	2.30854	-1.59682	-0.83307	2.30079	-0.55285	2.26703	-1.71726	-0.52432	-2.22848	1.73007
H	0.209	-0.51559	2.48895	-0.40628	-2.05472	1.90819	-0.42209	-2.08414	1.90695	0.03327	0.79804	-2.50291	0.06397	-0.74462	2.48245
H	-1.54091	-0.77405	2.32251	0.1846	-0.49914	2.49535	0.13905	-0.52924	2.52421	-1.7025	1.02476	-2.19896	-1.67377	-0.9809	2.20174
H	-2.62573	2.40127	-1.68624	-2.59948	2.4099	-1.6705	-1.66606	1.61139	-1.71234	-1.577	-1.74888	1.60708	-2.49487	2.58076	-1.51347
H	-4.44348	-0.73459	1.0118	-3.75719	-1.7811	0.95171	-3.83651	-1.74971	0.86951	-4.53035	0.70973	-0.71189	-3.82635	-1.80418	0.67358

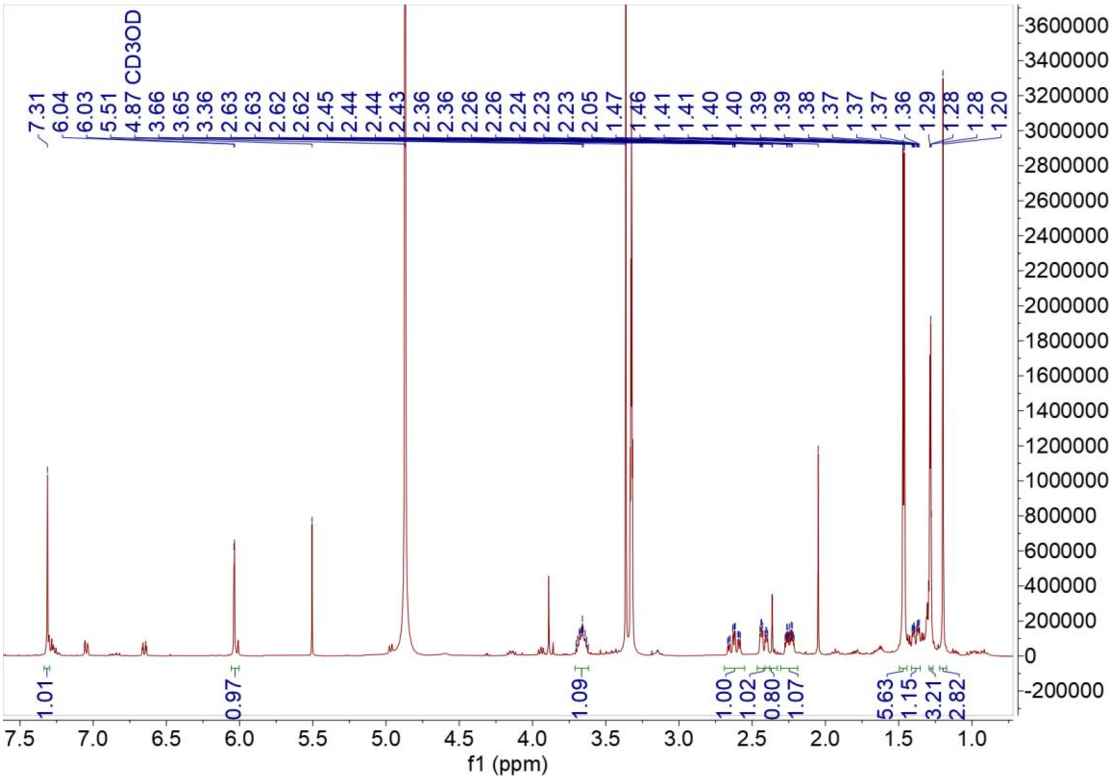

Figure S10. ^1H NMR spectrum (CD_3OD) of 2

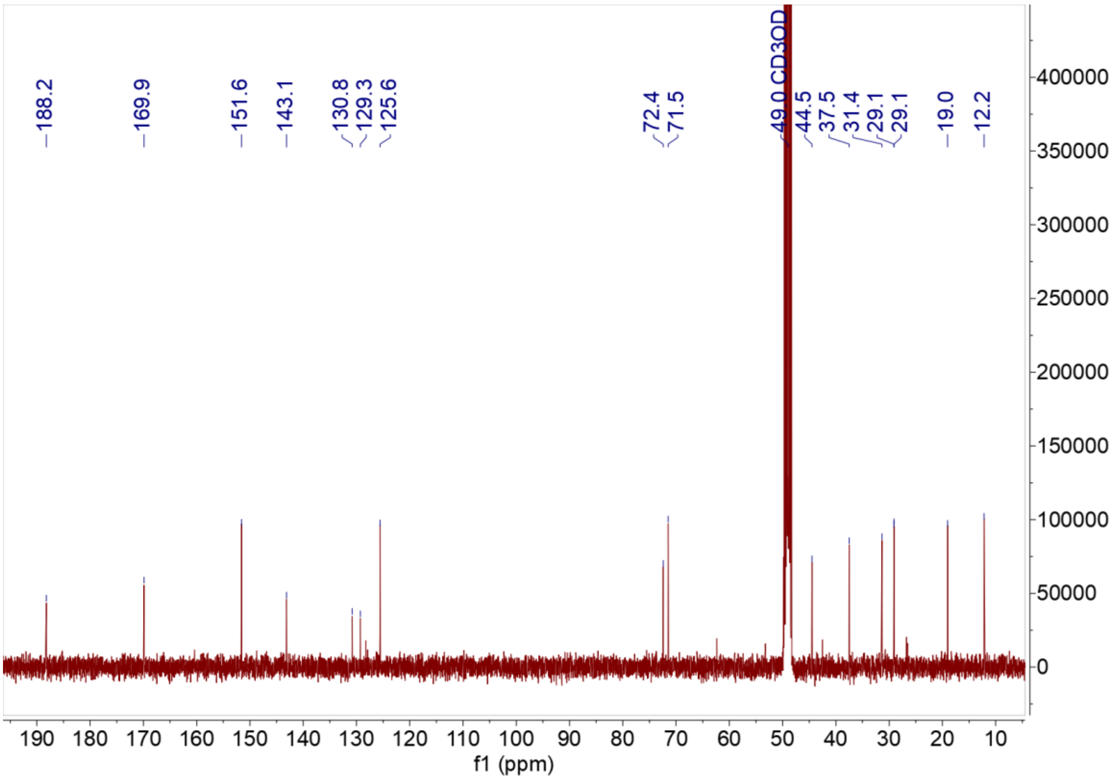

Figure S11. ^{13}C NMR spectrum (CD_3OD) of 2

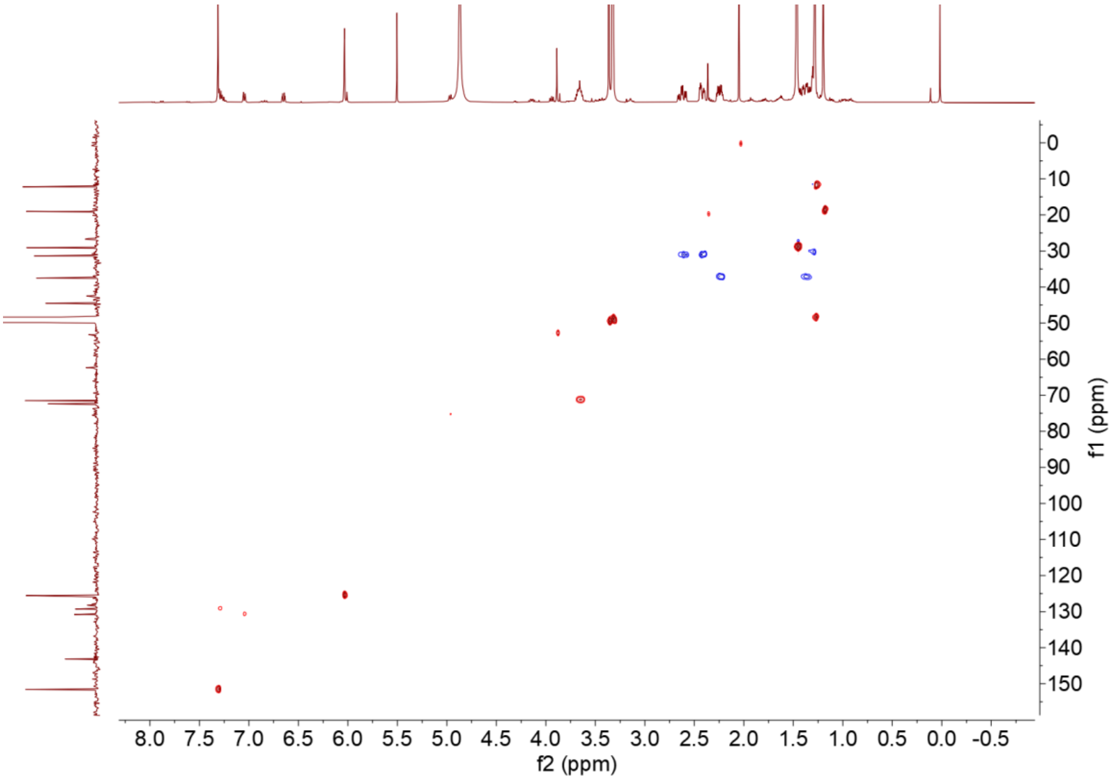

Figure S12. HSQC spectrum (CD_3OD) of 2

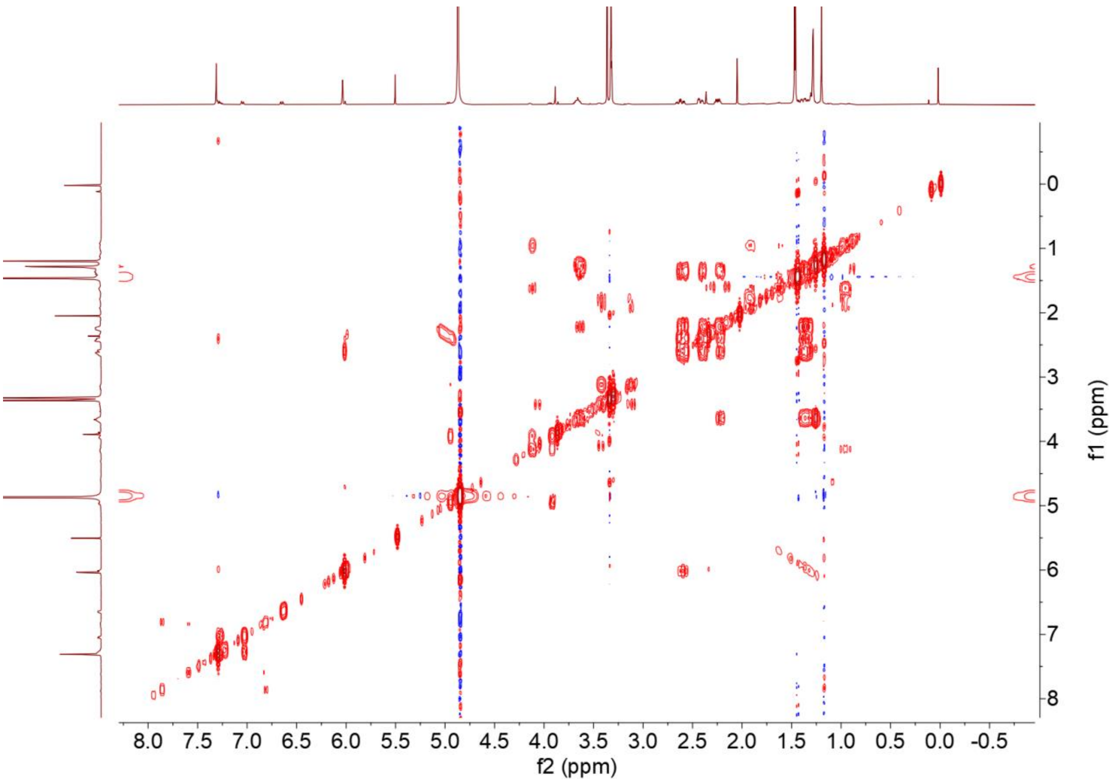

Figure S13. ^{1}H–^{1}H COSY spectrum (CD_3OD) of 2

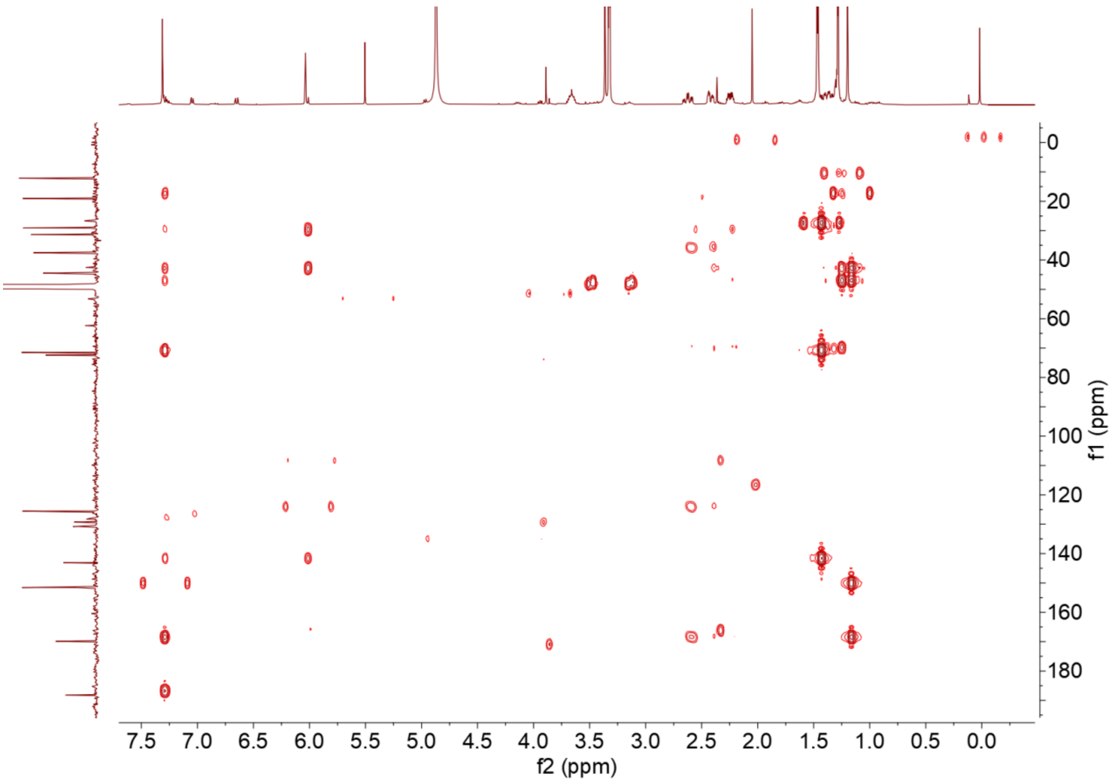

Figure S14. HMBC spectrum (CD_3OD) of 2

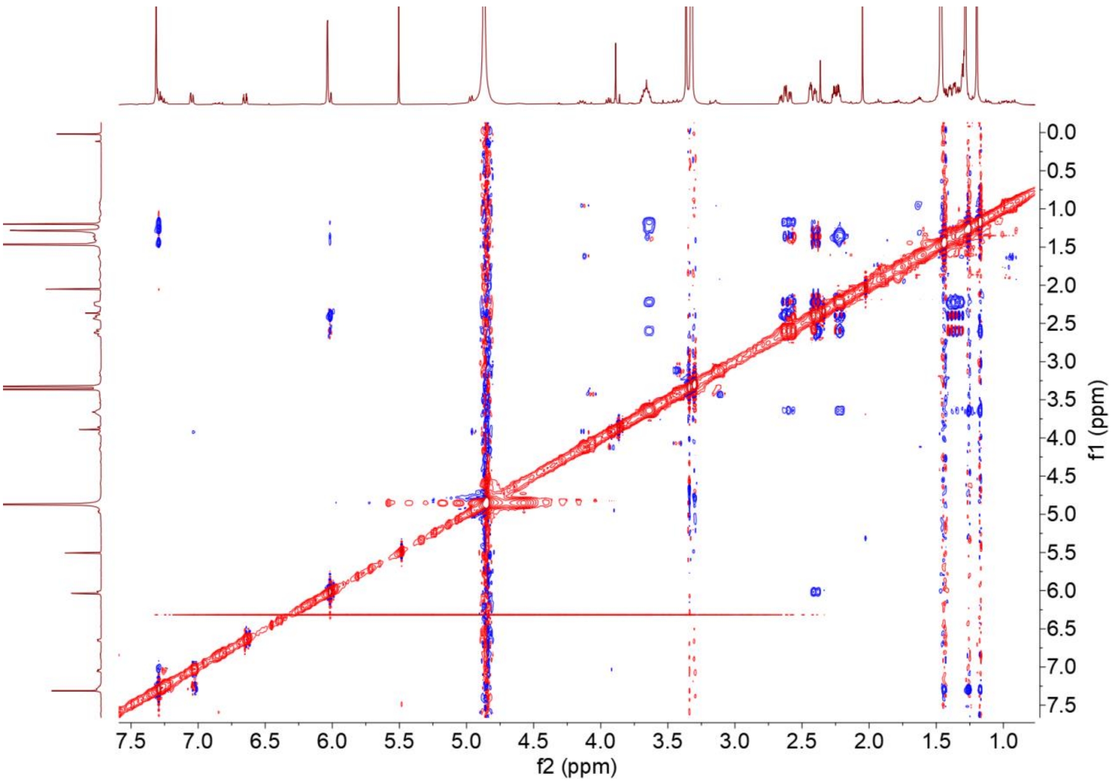

Figure S15. NOESY spectrum (CD_3OD) of 2

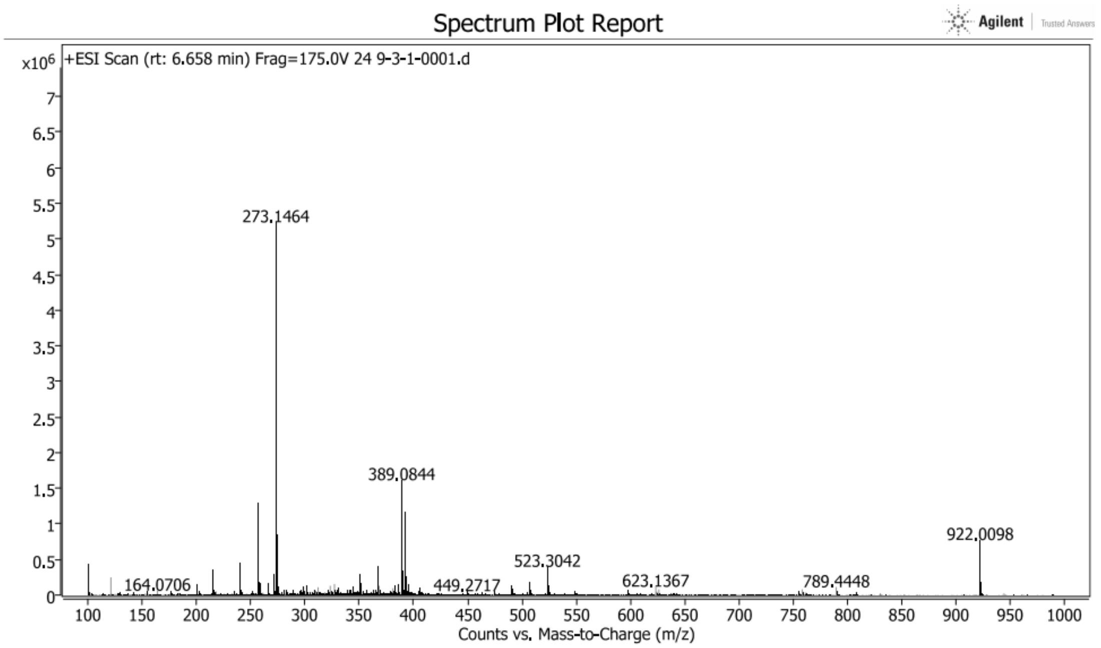

Figure S16. HRESIMS spectrum of 2

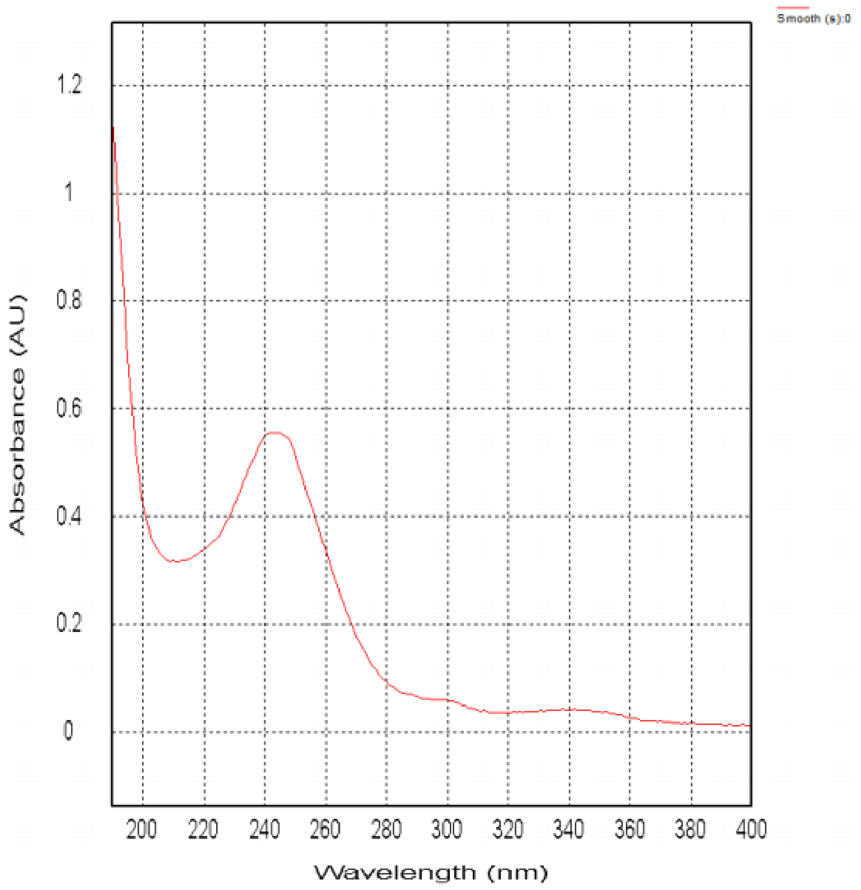

Figure S17. UV spectrum of compound 2

Table S3. Conformational analysis of the optimized isomers of 2 in methanol.

Conformations	G (hartree)	ΔG (kcal/mol)	Boltzmann distributions (%)
2-1	-810.475486	0	29.3
2-2	-810.475338	0.09287139	25.0
2-3	-810.475051	0.27296659	18.4
2-4	-810.474887	0.37587813	15.5
2-5	-810.473838	1.03413549	5.1
2-6	-810.473658	1.14708718	4.2
2-7	-810.473157	1.46146939	2.5

Table S4. The coordinates of the optimized conformers of 2.

	2-1			2-2			2-3			2-4		
C	-2.29378	1.91432	0.50799	-2.30422	1.92356	0.43786	-2.29866	1.92876	0.4256	-2.28412	1.92579	0.48518
C	-3.26158	1.19966	-0.44718	-3.2439	1.18236	-0.52442	-3.23274	1.18523	-0.53975	-3.25493	1.21304	-0.46696
C	-3.25024	-0.3122	-0.24265	-3.23577	-0.32277	-0.27749	-3.23174	-0.31683	-0.27451	-3.25388	-0.28942	-0.23693
C	-1.82966	-0.91278	-0.32475	-1.81294	-0.92341	-0.30698	-1.82004	-0.92343	-0.31063	-1.83775	-0.89881	-0.33498
C	-0.82182	-0.17323	0.64255	-0.82795	-0.15448	0.66168	-0.82861	-0.15452	0.64956	-0.8209	-0.17053	0.63001
C	-0.92786	1.30929	0.37897	-0.93311	1.32135	0.36172	-0.92914	1.32285	0.35362	-0.92022	1.31327	0.36756
C	0.56099	-0.70349	0.40359	0.56034	-0.68544	0.45837	0.558	-0.68858	0.43997	0.56008	-0.70493	0.3878
C	1.62438	0.0089	0.00327	1.63261	0.02442	0.07755	1.63414	0.02159	0.07139	1.6288	0.00547	-0.00168
C	1.45209	1.45697	-0.25642	1.47339	1.47714	-0.16281	1.48054	1.47694	-0.15614	1.46512	1.45628	-0.24966
C	0.13109	2.02959	-0.0253	0.13669	2.0407	-0.01461	0.14416	2.04184	-0.01356	0.14406	2.03118	-0.0271
O	-4.12927	-0.94865	-1.17562	-4.09082	-0.98709	-1.21331	-3.99296	-0.99921	-1.27754	-4.11611	-0.85321	-1.23185
C	3.02849	-0.58094	-0.1663	3.01961	-0.5858	-0.15124	3.0209	-0.59022	-0.15456	3.03124	-0.58974	-0.16659
C	3.9218	-0.1129	0.99512	3.17768	-1.96558	0.47665	3.17077	-1.97724	0.45925	3.91691	-0.13908	1.00749
C	3.03151	-2.10293	-0.24954	3.2973	-0.65858	-1.66271	3.30986	-0.64696	-1.66448	3.0275	-2.11076	-0.2669
O	2.38281	2.16906	-0.66069	2.4281	2.20076	-0.48167	2.43968	2.20111	-0.46046	2.40262	2.16832	-0.63836

	-1.8805	-2.4291	-0.1250	-1.8709	-2.4332	-0.0632			-0.0558	-1.8956	-2.4166	-0.1419
C	6	4	9	2	5	6	-1.8846	-2.432	6	3	3	1
	-1.1502	-0.4510	2.1364	-1.1851	-0.3970	2.1551	-1.1728	-0.3945	2.1472	-1.1431	-0.4517	2.1249
C	4	2	7	1	6	2	7	9	1	8	9	4
	3.5820	-0.1350	-1.4165		0.2344	0.4870	4.0118	0.2204	0.5003	3.5975	-0.1329	-1.4070
O	1	8	1	4.0133	4	5	9	1	7	5	6	5
	-2.2575	2.9827	0.2888	-2.2653	2.9867	0.1946	-2.2566		0.1803	-2.2397	2.9924	0.2590
H	2	9	7	4	2	5	2	2.9913	4	8	2	5
	-2.6567	1.8121	1.5364	-2.6942	1.8447	1.4585	-2.6918	1.8527	1.4453	-2.6508	1.8337	1.5135
H	4	8	6	4	7	2	6	6	8	6	1	4
	-2.9684	1.4163	-1.4817		1.3716	-1.5561	-2.9074	1.3512	-1.5719	-2.9615	1.4048	-1.5047
H	6	5	6	-2.9229	9	8	5	5	5	4	2	1
	-4.2792		-0.3151	-4.2653	1.5584	-0.4304	-4.2515	1.5757	-0.4530	-4.2689	1.5989	-0.3390
H	1	1.5747	5	7	9	9	3	2	8	6	9	9
	-3.6842	-0.5519	0.7321	-3.6932		0.6928	-3.6800	-0.5126	0.7091	-3.6680	-0.5037	0.7565
H	1	2	7	3	-0.5356	8	7	2	8	3	9	8
		-0.7134	-1.3406	-1.4224	-0.7545		-1.4422	-0.7622	-1.3276	-1.4860	-0.6962	-1.3533
H	-1.4632	5	7	3	7	-1.3195	9	2	5	3	7	8
	0.6824	-1.7584	0.6171	0.6738		0.6659	0.6682	-1.7476	0.6372	0.6792	-1.7610	0.5960
H	6	5	8	9	-1.7423	4	9	1	3	7	6	3
	0.0404	3.0922	-0.2293	0.0504	3.1015	-0.2299	0.0612	3.1040	-0.2232	0.0576		-0.2295
H	5	8	2	9	4	2	1	4	6	1	3.0945	7
	-3.8154	-0.7477	-2.0670	-3.7585		-2.1024	-4.9046	-0.6914	-1.2178	-4.3959		-0.9384
H	7	1	2	1	-0.8069	4	5	4	6	4	-1.7266	4
	3.5353	-0.4627	1.9557	4.2154	-2.2829	0.3603		-1.9596	1.5245	4.9263	-0.5365	0.8762
H	9	2	6	2	5	3	2.9317	1	6	2	8	6
	4.9319	-0.5072	0.8598	2.9457	-1.9370	1.5433		-2.7153	-0.0336		0.9494	1.0478
H	1	5	2	4	8	1	2.536	6	3	3.979	3	5
	3.9792	0.9762	1.0226	2.5417	-2.7108	-0.0038	4.2083	-2.2963	0.3465	3.5207	-0.4979	1.9607
H	2	7	6	9	5	8	4	7	1	1	1	9
H	2.3666	-2.4507	-1.0429	3.2489	0.3344	-2.1120	3.2679	0.3511	-2.1030	4.0409	-2.4455	-0.495

	4	1	5	7	7	3		5	3	6	1	
	2.7320		0.6916	2.5710	-1.3012	-2.1667	2.5855	-1.2816	-2.1811		-2.4461	-1.0691
H	9	-2.5667	4	8	8	9	1	7	8	2.3669	1	3
	4.0448	-2.4354	-0.4813	4.2984	-1.0634		4.3109	-1.0530	-1.8285	2.7190	-2.5839	0.6666
H	9	8	3	1	7	-1.8299	6	7	7	9	3	8
	-0.9165	-2.9054	-0.3073	-2.5725		-0.7666	-2.2663	-2.6565	0.9431		-2.6872	
H	7	1	8	3	-2.8807	3	2	4	1	-2.3303	1	0.8239
	-2.2071	-2.6934	0.8835	-0.9041		-0.2064	-2.5637	-2.8855	-0.7762	-2.5118	-2.8655	-0.9227
H	9	9	2	6	-2.9171	5	9	7	7	5	7	6
	-2.5992	-2.8567	-0.8235	-2.2236	-2.6667	0.9441	-0.9135	-2.9160	-0.1655	-0.9151	-2.8870	-0.2148
H	5	1	8	7	1	4	4	1	9	5	2	1
	-0.4994	0.1480	2.7759		0.2217	2.7920	-1.0137	-1.4389	2.4158	-1.0058	-1.5087	2.3546
H	7	3	3	-0.5507	3	8	5	5	6	1	3	9
	-2.1837	-0.2085	2.3814	-2.2249		2.3726	-0.5261	0.2176	2.7780	-0.4698	0.1223	
H	5	2	1	4	-0.1549	4	4	2	4	1	4	2.7638
	-0.9849	-1.5008			-1.4394	2.4267		-0.1429	2.3743	-2.1669	-0.1826	2.3818
H	6	1	2.3787	-1.0186	5	6	-2.2083	7	2	7	8	6
	3.5108	0.8333	-1.3967	3.8641	1.1307	0.1439	3.8676	1.1207	0.1658	3.5301	0.8355	-1.3778
H	7	9	7	2	2	4	7	9	8	5	7	3
	2-5			2-6			2-7					
	-2.3085	1.9132	0.4929	-2.3099		0.4525	-2.3049	1.9265	0.4408			
C	3	3	7	5	1.922	1	8	8	4			
	-3.2631	1.1854		-3.2587		-0.4976	-3.2481	1.1790	-0.5126			
C	6	3	-0.4651	8	1.1766	1	5	1	2			
	-3.2394	-0.3251	-0.2523	-3.2375	-0.3296	-0.2556	-3.2331	-0.3243	-0.2530			
C	4	5	4	1	3	5	5	5	5			
	-1.8135	-0.9141	-0.3213	-1.8115	-0.9199	-0.3057	-1.8179	-0.9200	-0.3100			
C	3	7	8	4	1	5	2	4	7			
	-0.8172	-0.1605	0.6455	-0.8203	-0.1492	0.6534	-0.8208	-0.1495	0.6415			
C	7	4	1	1	9	3	8	8	2			
C	-0.9355	1.3205	0.3755	-0.9363	1.3266	0.3567	-0.9327	1.3278	0.3491			

	2	4	9	3	2	3	1	9	
	0.5719	-0.6747	0.4137	0.5680	-0.6713		0.5659	-0.6742	0.4172
C	2	8	5	4	7	0.4352	7	9	9
	1.6352	0.0473	0.0321	1.6303	0.0435	0.0355	1.6320	0.0413	0.0297
C	3	2	6	1	3	9	3	8	6
	1.4595	1.4949		1.4668		-0.2284	1.4740	1.4929	
C	5	9	-0.2385	6	1.4923	5	1	8	-0.2213
	0.1198	2.0468	-0.0230	0.1238	2.0464		0.1308	2.0477	-0.0389
C	8	5	3	8	2	-0.0404	9	8	2
	-4.1078	-0.9743	-1.1872	-4.1013	-0.9961	-1.1828	-4.0044	-1.0080	-1.2482
O	9	7	1	4	2	2	8	2	4
	3.0141		-0.1467	2.9997	-0.5978	-0.1470	3.0009	-0.6017	-0.1507
C	3	-0.5974	1	4	3	6	9	6	3
	3.4272	-0.5663	-1.6184	3.4590	-0.4876	-1.6074		-0.4758	
C	5	8	3	1	7	5	3.4718	5	-1.6061
	4.0588	0.0856	0.7438	4.0264	0.0227	0.8098	4.0217	0.0050	0.8213
C	6	7	7	7	6	3	2	2	8
	2.3732		-0.6393	2.3933	2.2196	-0.5937		2.2217	-0.5720
O	4	2.2194	4	3	6	4	2.4051	5	1
		-2.4294	-0.1107	-1.8525	-2.4314	-0.0699	-1.8645	-2.4306	-0.0643
C	-1.8546	6	1	8	6	6	8	5	7
	-1.1493	-0.4372			-0.3979	2.1492	-1.1488	-0.3962	
C	4	4	2.1385	-1.1608	8	3	4	3	2.1412
	2.9612	-1.9942	0.1910	2.8441			2.8396	-1.9977	0.1619
O	8	3	4	9	-1.9905	0.183	4	9	2
	-2.2811	2.9812	0.2694		2.9855	0.2087		2.9895	0.1952
H	9	1	7	-2.2803	4	7	-2.2725	8	6
	-2.6778	1.8124	1.5194	-2.6865	1.8412	1.4781	-2.6847	1.8482	1.4656
H	9	1	7	5	1	6	4	2	5
	-2.9642	1.3990	-1.4987		1.3700	-1.5333	-2.9383	1.3500	-1.5488
H	6	4	9	-2.9531	6	6	6	3	7

	-4.2855	1.5512	-0.3431	-4.2816	1.5453	-0.3893		1.5617	-0.4110
H	4	7	1	7	8	7	-4.2687	7	8
	-3.6777	-0.5630	0.7210	-3.6812	-0.5485	0.7197	-3.6672	-0.5267	0.7357
H	6	1	9	8	8	5	1	9	4
	-1.4429	-0.7195	-1.3369	-1.4348	-0.7426	-1.3222	-1.4537	-0.7494	
H	6	6	1	9	3	6	1	5	-1.3306
	0.7149	-1.7328	0.5950	0.7157	-1.7215	0.6489	0.7107	-1.7267	0.6212
H	2	8	8	7	6	2	7	1	3
	0.0200		-0.2315	0.0286	3.1057	-0.2609	0.0386	3.1085	-0.2538
H	8	3.1082	3	3	4	6	1	1	1
	-3.7887		-2.0774	-3.7771	-0.8158	-2.0749		-0.7023	-1.1772
H	8	-0.7768	6	7	3	4	-4.9161	1	6
		-1.1161		4.4232	-0.9901	-1.7329	4.4349	-0.9807	-1.7303
H	2.7008	7	-2.2212	3	8	3	2	6	3
		0.4597	-1.9776	2.7314	-0.9709	-2.2631	2.7473	-0.9481	-2.2731
H	3.4802	4	6	7	6	7	6	5	3
	4.4050	-1.0389	-1.7367	3.5707	0.5534		3.5899		-1.8908
H	7	2	1	9	1	-1.9058	6	0.5683	7
	5.0184	-0.4247	0.6342	4.1616	1.0834	0.6048	4.1598		0.6308
H	7	6	5	2	4	9	4	1.068	1
		0.0327	1.7946	4.9923	-0.4790	0.6949	4.9877	-0.4967	0.7073
H	3.7565	2	7	6	5	7	2	6	8
		1.1336	0.4760	3.6948		1.8429	3.6821	-0.1323	1.8503
H	4.1765	8	3	6	-0.102	4	5	3	1
	-0.8834	-2.8983	-0.2724		-2.9011	-0.2204	-2.5451	-2.8888	-0.7807
H	4	2	7	-0.88	6	8	4	7	3
	-2.1948	-2.6877	0.8950	-2.1958	-2.6733	0.9388	-0.8877		-0.1848
H	4	7	6	9	5	7	4	-2.9002	9
	-2.5582	-2.8699	-0.8165		-2.8843	-0.7706	-2.2340	-2.6650	
H	9	3	2	-2.5535	7	8	9	1	0.9371
H	-2.1873	-0.2082	2.3787	-2.1991	-0.1611	2.3801	-2.1828	-0.1503	2.3815

	1	2	3	9	6		7	5	1
		-1.4837	2.3853	-0.9860	-1.4403	2.4160	-0.9811	-1.4405	2.4049
H	-0.9701	7	1	3	4	6	8	8	6
	-0.5095	0.1731	2.7784	-0.5222	0.2219	2.7812	-0.4983	0.2172	2.7672
H	9	6	8	6	4	4	5	7	3
	2.9036	-2.0712	1.1518	3.7045		0.0665	3.6999	-2.4183	0.0474
H	7	7	2	7	-2.4104	1	6	4	2

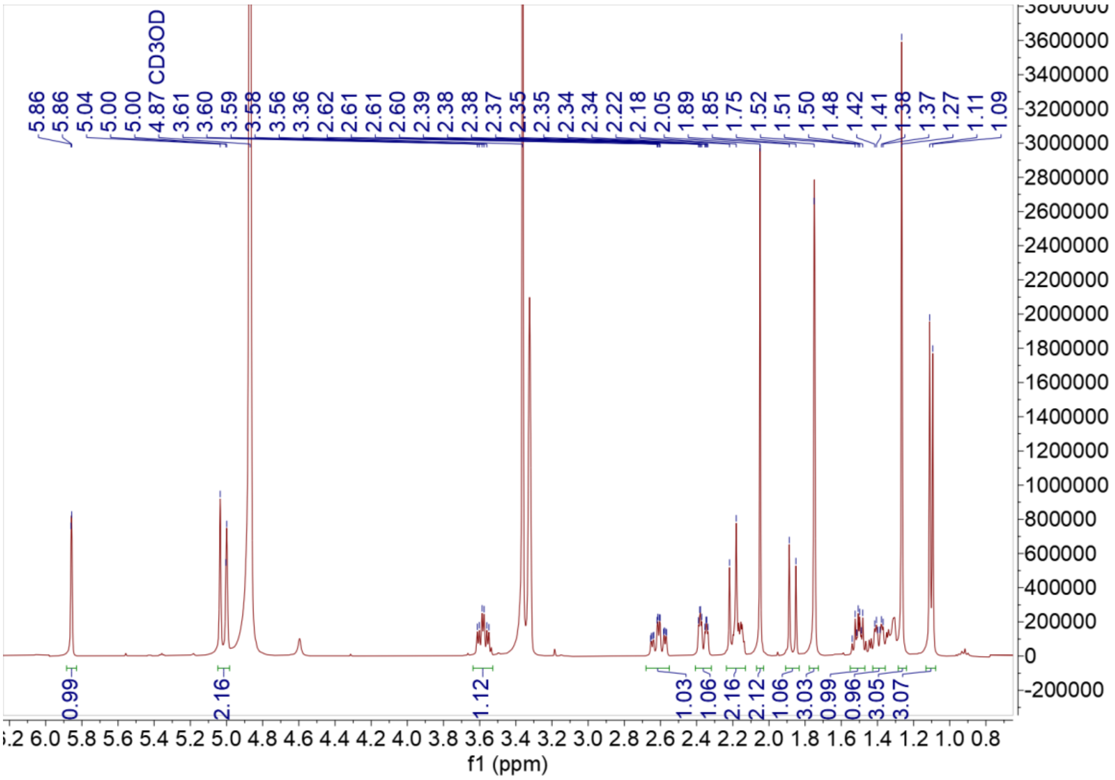

Figure S18. ¹H NMR spectrum (CD_3OD) of 3

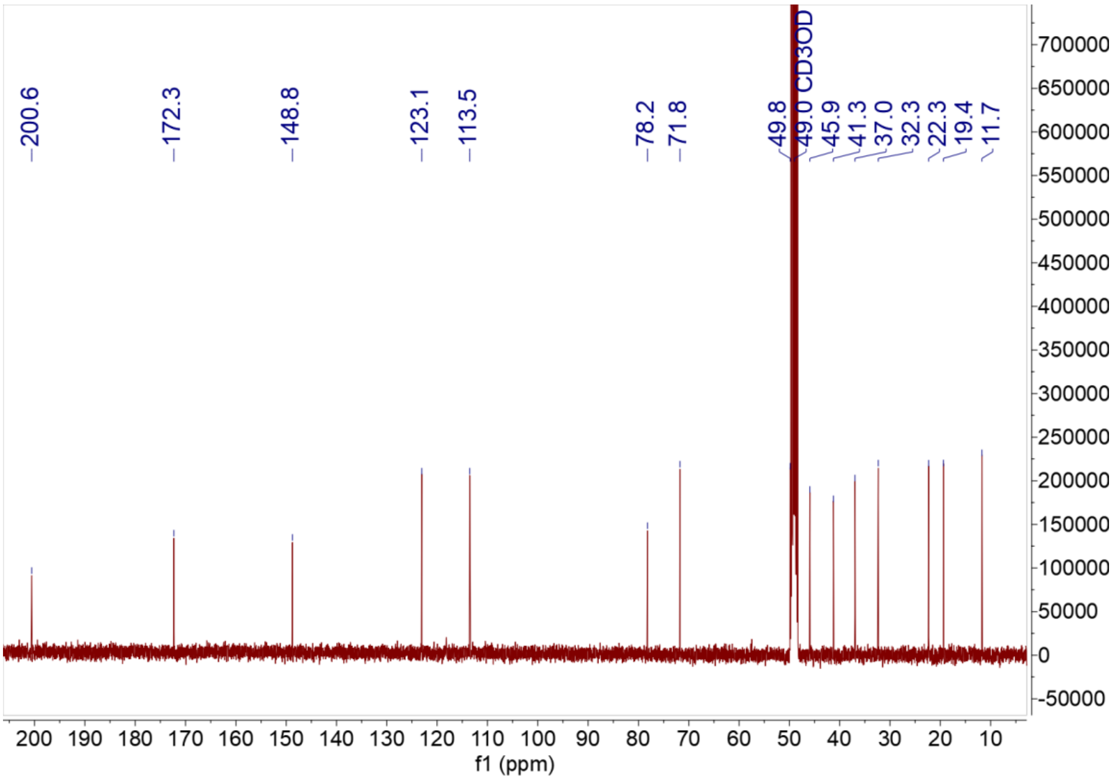

Figure S19. ^{13}C NMR spectrum (CD_3OD) of 3

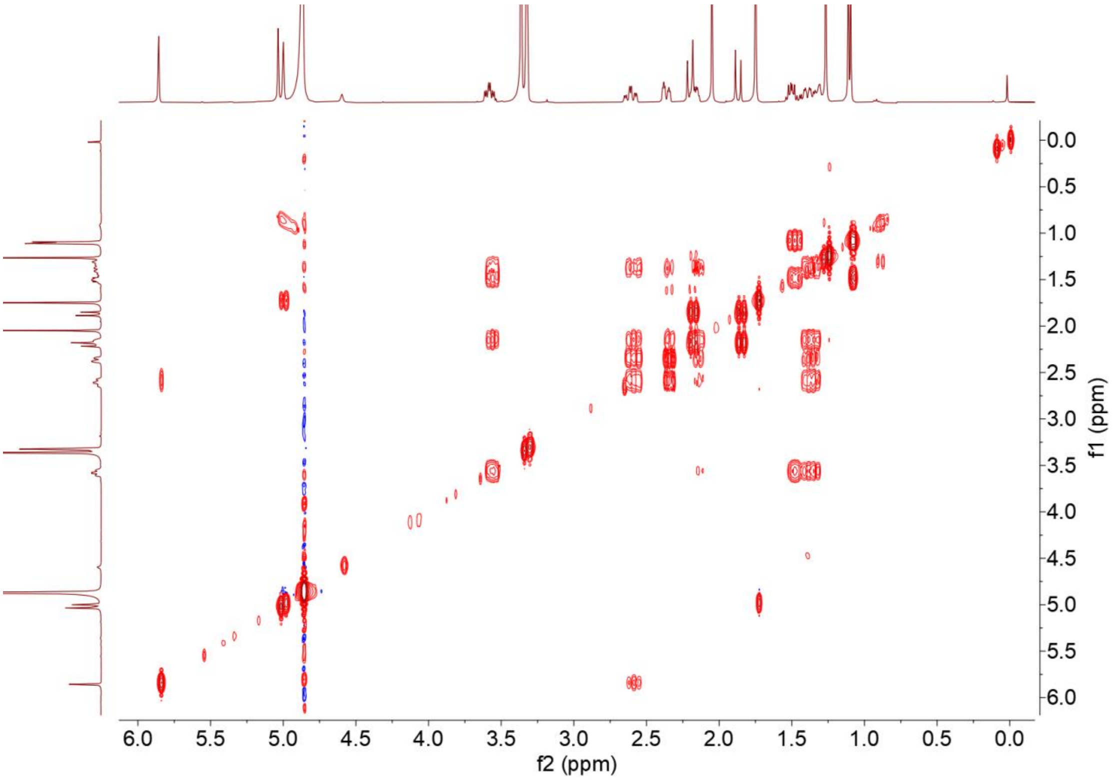

Figure S20. ^{1}H–^{1}H COSY spectrum (CD_3OD) of 3

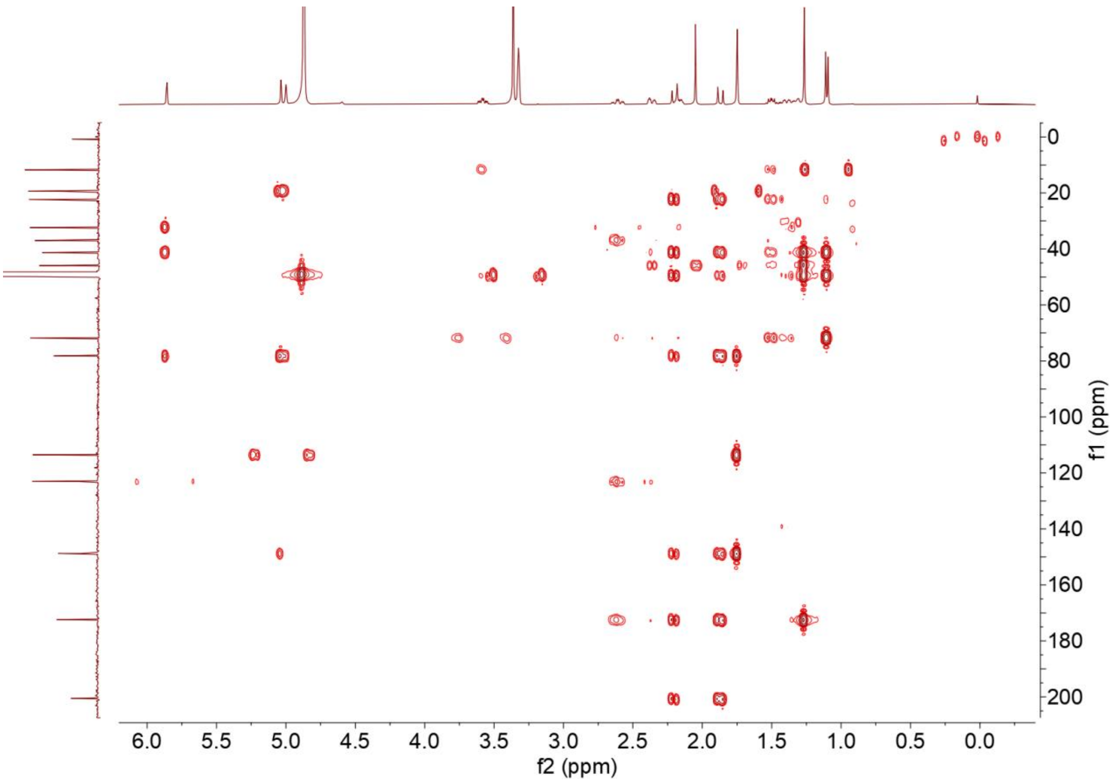

Figure S21. HMBC spectrum (CD_3OD) of 3

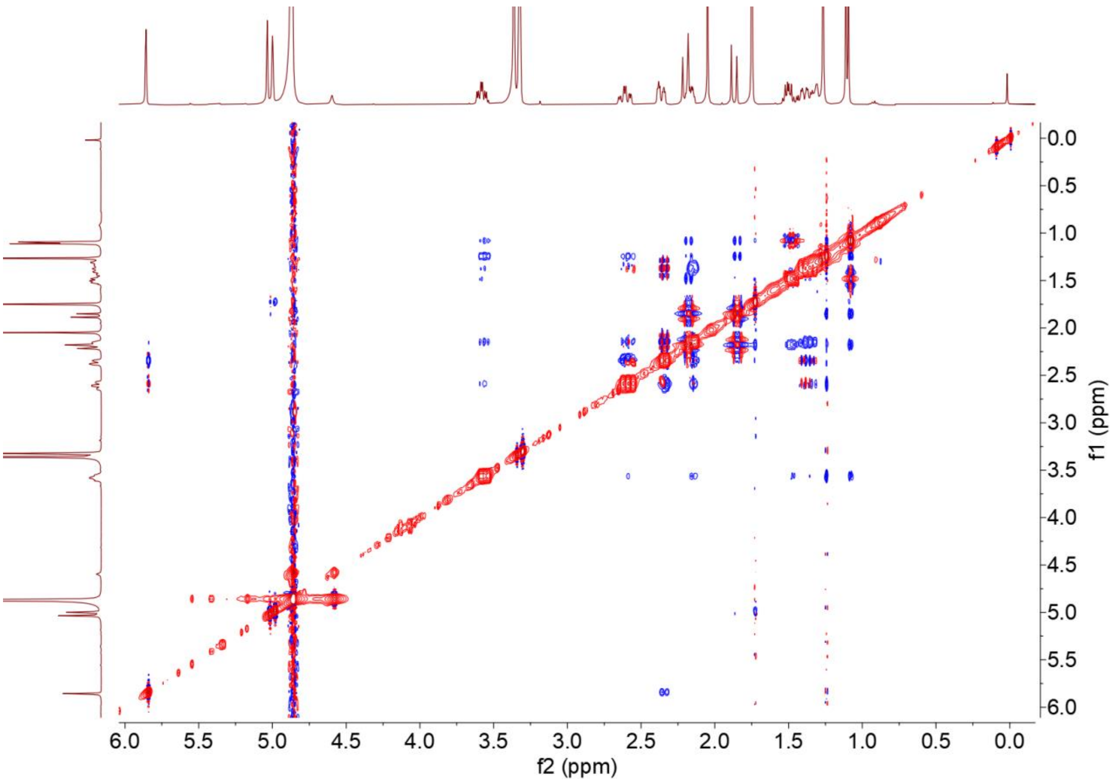

Figure S22. NOESY spectrum (CD_3OD) of 3

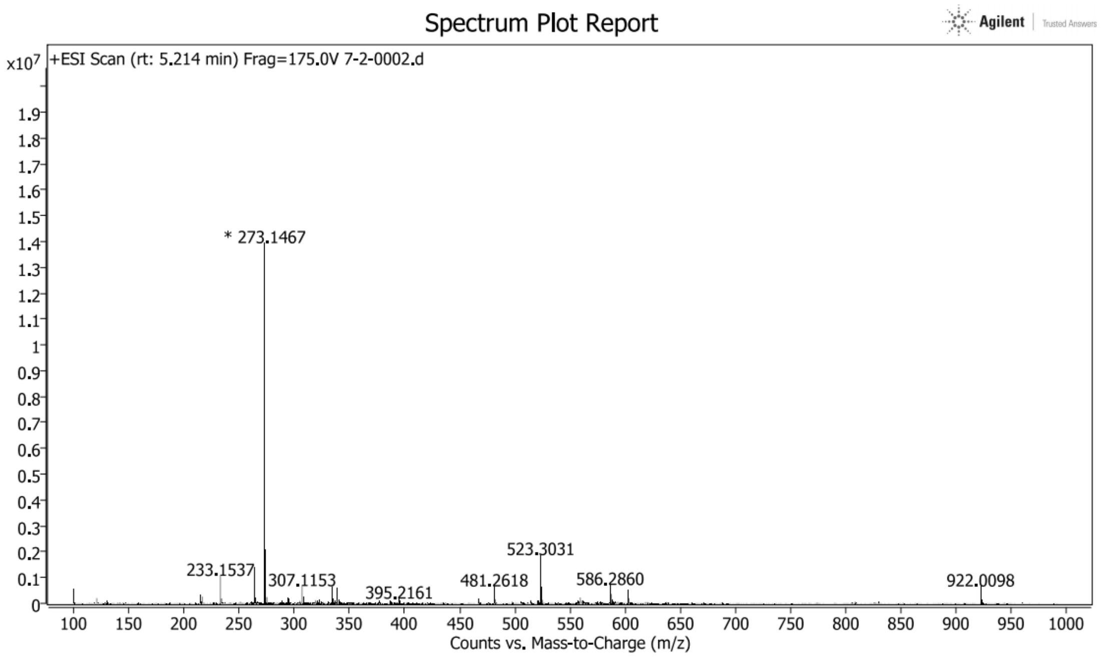

Figure S23. HRESIMS spectrum of 3

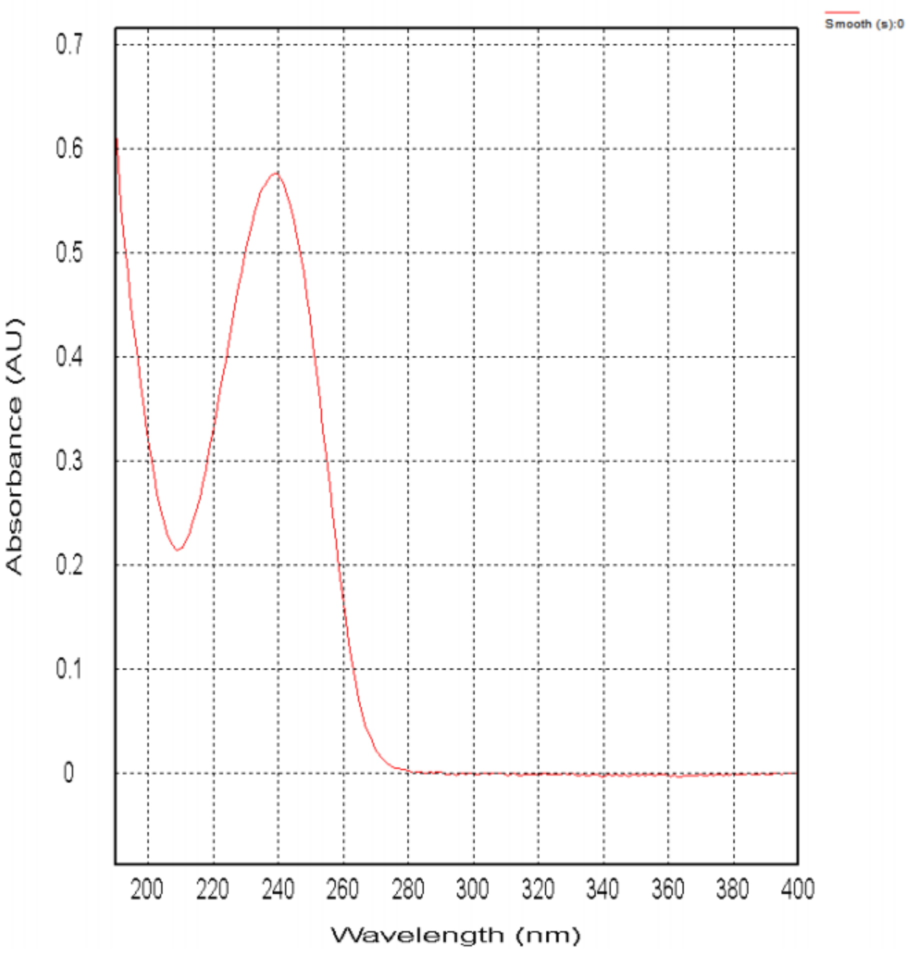

Figure S24. UV spectrum of compound 3

Table S5. Conformational analysis of the optimized isomers of 3 in methanol.

Conformations	G (hartree)	ΔG (kcal/mol)	Boltzmann distributions (%)
3-1	-810.457129	0	35.8
3-2	-810.456855	0.171937576	26.8
3-3	-810.456506	0.390938356	18.5
3-4	-810.456446	0.42858892	17.4
3-5	-810.454167	1.858682843	1.5

Table S6. The coordinates of the optimized conformers of 3.

	3-1			3-2			3-3			3-4			3-5		
C	1.70881	-2.14464	-0.38218	1.70332	-2.14766	-0.37261	-2.0074	1.9928	0.21506	1.69552	-2.15392	-0.36725	1.71235	-2.14063	-0.34924
C	2.49431	-1.08794	-1.17841	2.48263	-1.09188	-1.17576	-3.10787	1.27619	-0.57335	2.4866	-1.11108	-1.17487	2.53396	-1.08841	-1.11671
C	2.73184	0.17478	-0.35507	2.72384	0.17016	-0.35227	-3.22567	-0.1829	-0.15226	2.73068	0.14847	-0.35917	2.73117	0.19692	-0.31409
C	1.43539	0.77167	0.23602	1.43441	0.77384	0.22872	-1.88066	-0.91766	-0.29461	1.43317	0.76355	0.21129	1.40681	0.78094	0.2256
C	0.56152	-0.30434	0.97598	0.56045	-0.29707	0.97513	-0.70628	-0.21421	0.47777	0.55886	-0.29376	0.97574	0.56045	-0.29782	0.99358
C	0.42443	-1.51309	0.06335	0.42042	-1.51264	0.07184	-0.69459	1.26542	0.13363	0.41519	-1.51365	0.07853	0.4246	-1.50088	0.07514
C	-0.83014	0.23389	1.38073	-0.83048	0.24641	1.3755	0.60955	-0.88881	0.02808	-0.83077	0.25317	1.37614	-0.83042	0.21079	1.42778
C	-1.91889	0.1864	0.30467	-1.92011	0.19154	0.30098	1.90712	-0.1147	0.26121	-1.92177	0.19642	0.30343	-1.92715	0.20472	0.36066
C	-2.0123	-1.25674	-0.19989	-2.01624	-1.25543	-0.19133	1.76947	1.36919	-0.13455	-2.02181	-1.2523	-0.18232	-2.00913	-1.21058	-0.22648
C	-0.7567	-1.95892	-0.40524	-0.76187	-1.96086	-0.39172	0.43122	1.9378	-0.17387	-0.76884	-1.96073	-0.38178	-0.7537	-1.91815	-0.42755
O	3.43342	1.15304	-1.13197	3.31686	1.18996	-1.16664	-4.26217	-0.83988	-0.89091	3.38668	1.07588	-1.23378	3.43432	1.16515	-1.10212
C	-1.70848	1.16699	-0.85507	-1.70965	1.16244	-0.86654	3.04768	-0.7889	-0.49734	-1.71021	1.16192	-0.86836	-1.70968	1.2526	-0.75444
C	-1.58918	0.78526	-2.12683	-1.59956	0.77068	-2.136	3.92669	-1.55662	0.1448	-1.61236	0.76522	-2.13728	-1.78775	2.5432	-0.43525
C	-1.71293	2.62144	-0.46527	-1.70233	2.61958	-0.48686	3.10622	-0.57455	-1.98714	-1.68786	2.62014	-0.4933	-1.47601	0.80171	-2.17265
O	-3.11036	-1.73868	-0.44995	-3.11527	-1.73776	-0.4369	2.77189	2.05412	-0.29951	-3.12204	-1.73331	-0.42484	-3.10073	-1.67082	-0.53856
C	1.78789	1.98012	1.10851	1.78829	1.98591	1.09703	-2.03506	-2.39894	0.06155	1.78257	1.99275	1.05855	1.67948	2.04548	1.04144
C	1.25143	-0.75337	2.28813	1.24687	-0.73892	2.29188	-0.88541	-0.34293	2.01052	1.25156	-0.7213	2.29346	1.27872	-0.73259	2.295

O	-3.15421	0.51087	0.94642	-3.15431	0.52314	0.94181	2.14687	-0.09673	1.67691	-3.15421	0.53456	0.94426	-3.15245	0.46592	1.04489
H	1.5132	-3.02274	-0.99967	1.5062	-3.02833	-0.98591	-1.88932	3.02206	-0.12863	1.49255	-3.03524	-0.97762	1.5228	-3.00946	-0.98168
H	2.30443	-2.47508	0.47393	2.30092	-2.47423	0.48368	-2.30705	2.0499	1.26902	2.29107	-2.48252	0.48981	2.28001	-2.48844	0.51863
H	1.92686	-0.82483	-2.07942	1.91598	-0.81898	-2.07183	-2.87631	1.31717	-1.64507	1.92562	-0.84274	-2.07642	2.01093	-0.84183	-2.04889
H	3.45533	-1.49075	-1.50725	3.43999	-1.50483	-1.50889	-4.06776	1.77865	-0.43138	3.44479	-1.52484	-1.49823	3.50948	-1.49388	-1.39546
H	3.42436	-0.05621	0.45935	3.40975	-0.0687	0.47185	-3.56739	-0.2386	0.88541	3.4074	-0.09312	0.47076	3.40865	0.00184	0.52132
H	0.84483	1.11989	-0.61838	0.85278	1.11803	-0.63121	-1.61832	-0.85452	-1.36093	0.854	1.09093	-0.65617	0.8254	1.07719	-0.65362
H	-0.75174	1.24392	1.7819	-0.75134	1.25967	1.76802	0.53435	-1.0884	-1.04309	-0.75149	1.26772	1.76533	-0.75612	1.20796	1.86121
H	-1.20979	-0.38195	2.20111	-1.21044	-0.36196	2.20124	0.72325	-1.85635	0.51926	-1.21007	-0.35181	2.20475	-1.19323	-0.44358	2.22589
H	-0.80803	-2.86244	-1.0061	-0.81482	-2.86893	-0.98556	0.39035	3.00148	-0.39266	-0.82424	-2.86995	-0.97364	-0.8039	-2.80413	-1.05419
H	2.86917	1.40168	-1.87548	4.17921	0.87262	-1.45877	-4.02271	-0.81531	-1.82633	3.80709	1.75727	-0.69854	2.88464	1.38218	-1.86637
H	-1.47853	1.51835	-2.91956	-1.48823	1.4971	-2.93472	4.72286	-2.06564	-0.38857	-1.50107	1.48812	-2.93919	-1.66283	3.31954	-1.1831
H	-1.58666	-0.25308	-2.43631	-1.60451	-0.26998	-2.43759	3.8763	-1.70751	1.21621	-1.62806	-0.27633	-2.43545	-1.99304	2.86971	0.57802
H	-2.58822	2.85069	0.1453	-2.56822	2.85733	0.13383	3.36925	0.45999	-2.21937	-2.54208	2.86536	0.14055	-0.58627	0.17413	-2.26873
H	-0.8319	2.88459	0.12647	-0.81173	2.8824	0.09052	3.84935	-1.22779	-2.44645	-0.78618	2.87934	0.0683	-2.31671	0.20331	-2.53742
H	-1.72262	3.26253	-1.34783	-1.72093	3.25457	-1.37367	2.14377	-0.772	-2.46848	-1.71705	3.25257	-1.38167	-1.35814	1.65908	-2.83659
H	0.90978	2.46413	1.5372	0.91229	2.45394	1.54749	-2.8107	-2.84555	-0.55991	2.5337	1.75678	1.81728	2.22939	1.82537	1.95979
H	2.31448	2.72067	0.50686	2.28853	2.73616	0.48575	-2.33553	-2.53103	1.10376	0.91941	2.41374	1.57424	0.75974	2.56593	1.30965
H	2.44768	1.69825	1.93281	2.47014	1.71157	1.90598	-1.11375	-2.96055	-0.09845	2.184	2.78747	0.42655	2.28754	2.73004	0.4499
H	0.75134	-1.63537	2.69458	2.29971	-0.98762	2.16429	-1.8561	0.02608	2.34415	2.29991	-0.98598	2.16163	0.79807	-1.62115	2.71067
H	2.30595	-0.99425	2.15808	1.18261	0.06152	3.03082	-0.80122	-1.38692	2.31515	1.20439	0.09342	3.01833	2.33451	-0.95841	2.15274
H	1.18206	0.0393	3.03483	0.74057	-1.61354	2.7065	-0.10582	0.21444	2.52589	0.73843	-1.58182	2.72862	1.20729	0.05983	3.04166
H	-3.8249	-0.01829	0.48506	-3.82649	-0.00723	0.48411	2.97956	0.37332	1.8215	-3.82873	0.00525	0.48895	-3.83955	0.01898	0.52627

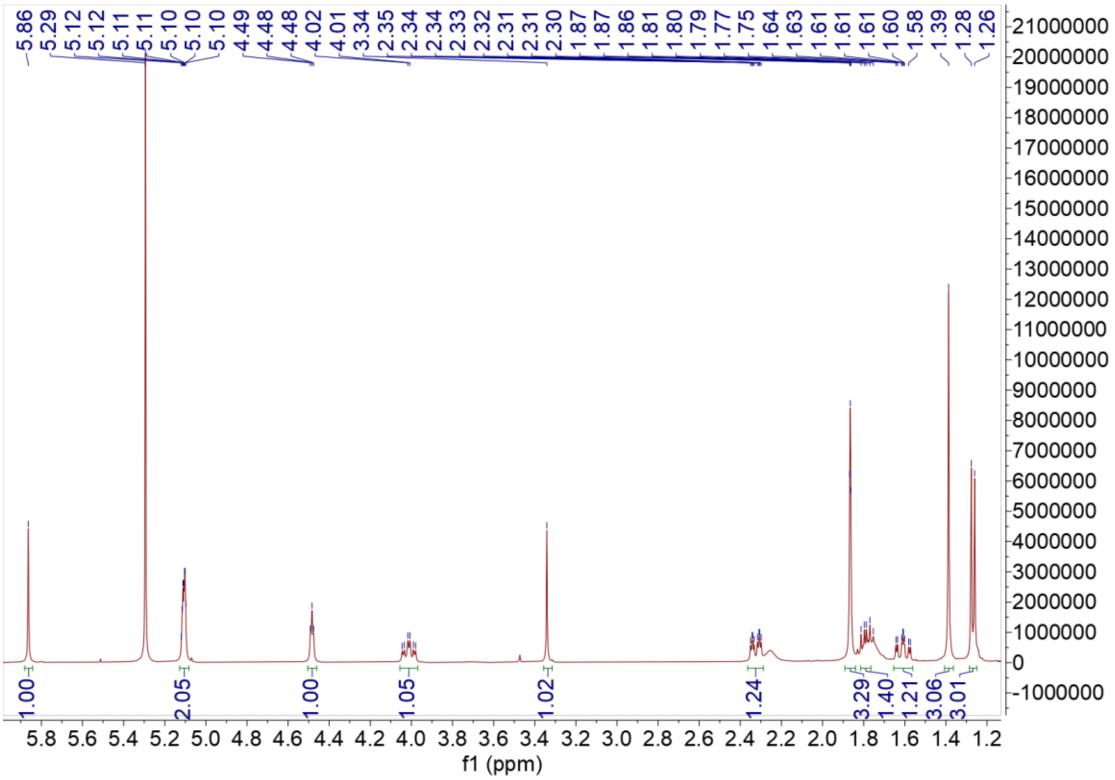

Figure S25. ^{1}H NMR spectrum ($CDCl_3$) of 4

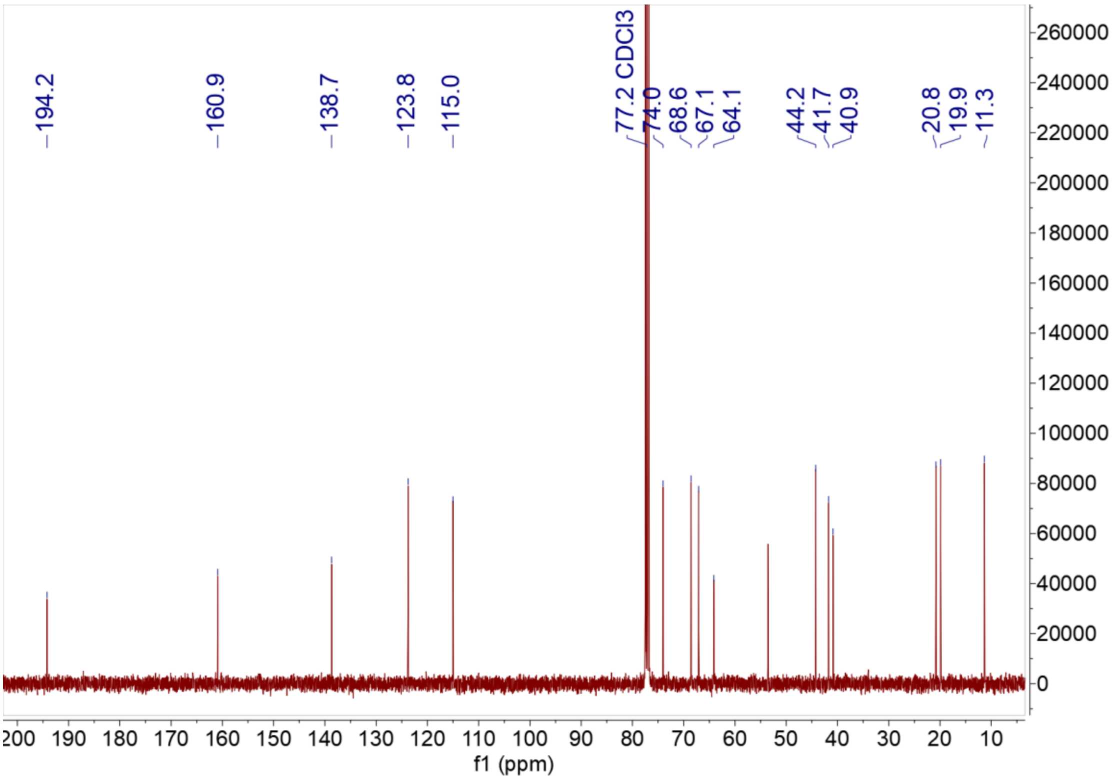

Figure S26. ^{13}C NMR spectrum ($CDCl_3$) of 4

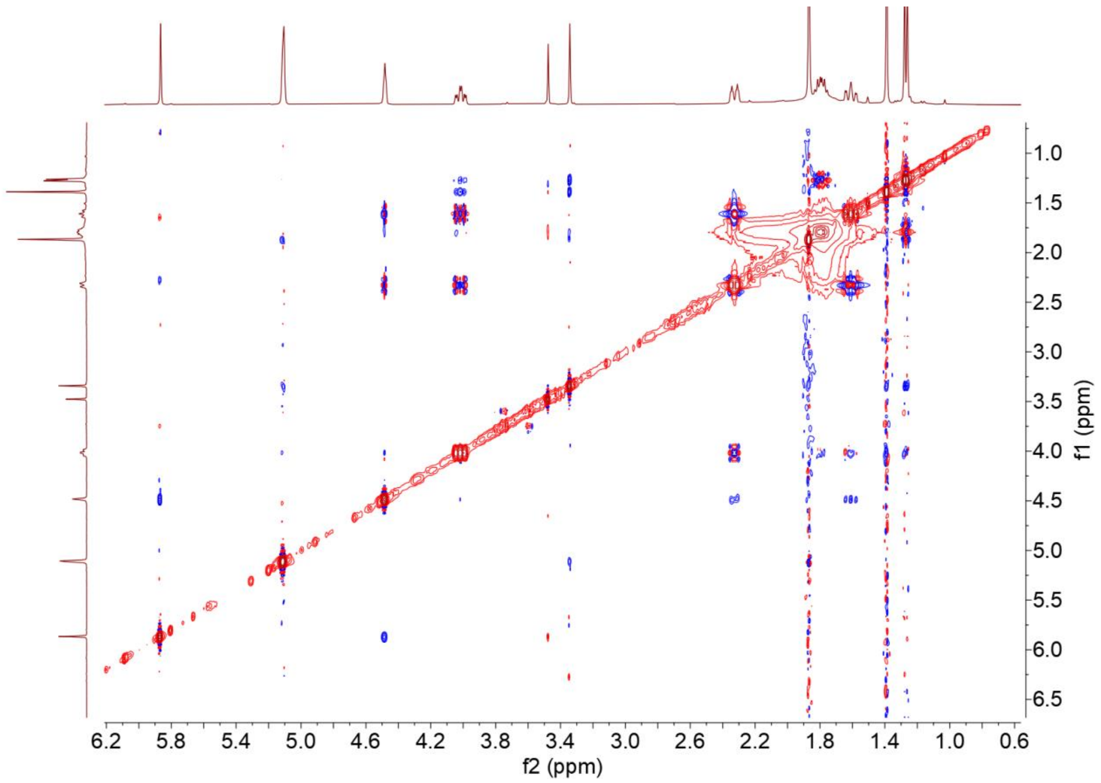

Figure S27. NOESY spectrum ($CDCl_3$) of 4

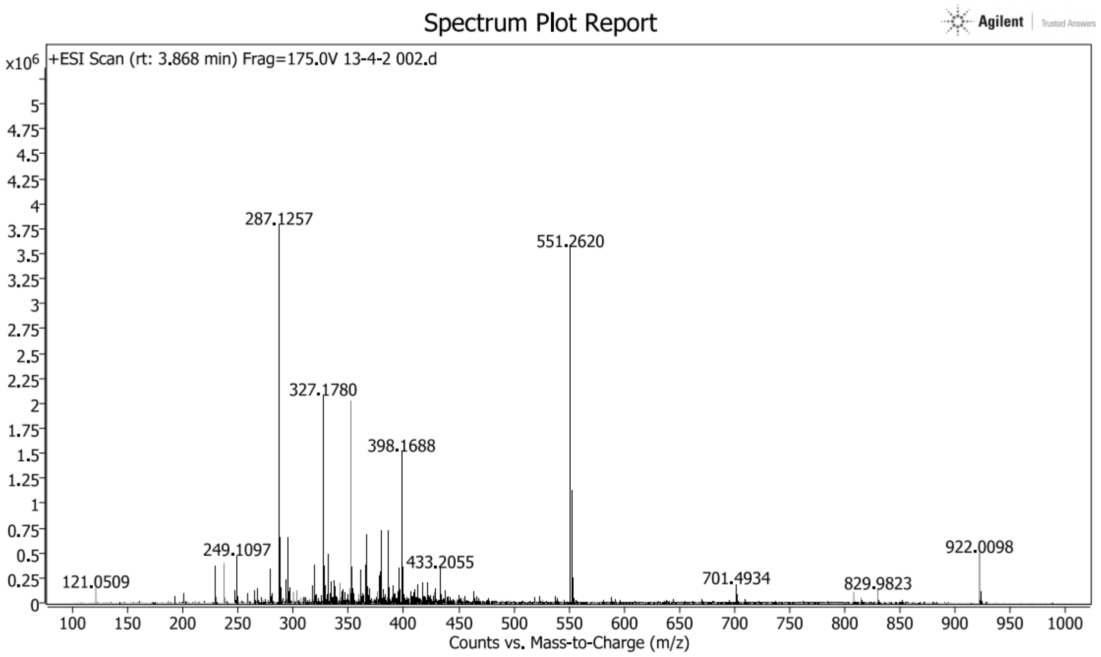

Figure S28. HRESIMS spectrum of 4

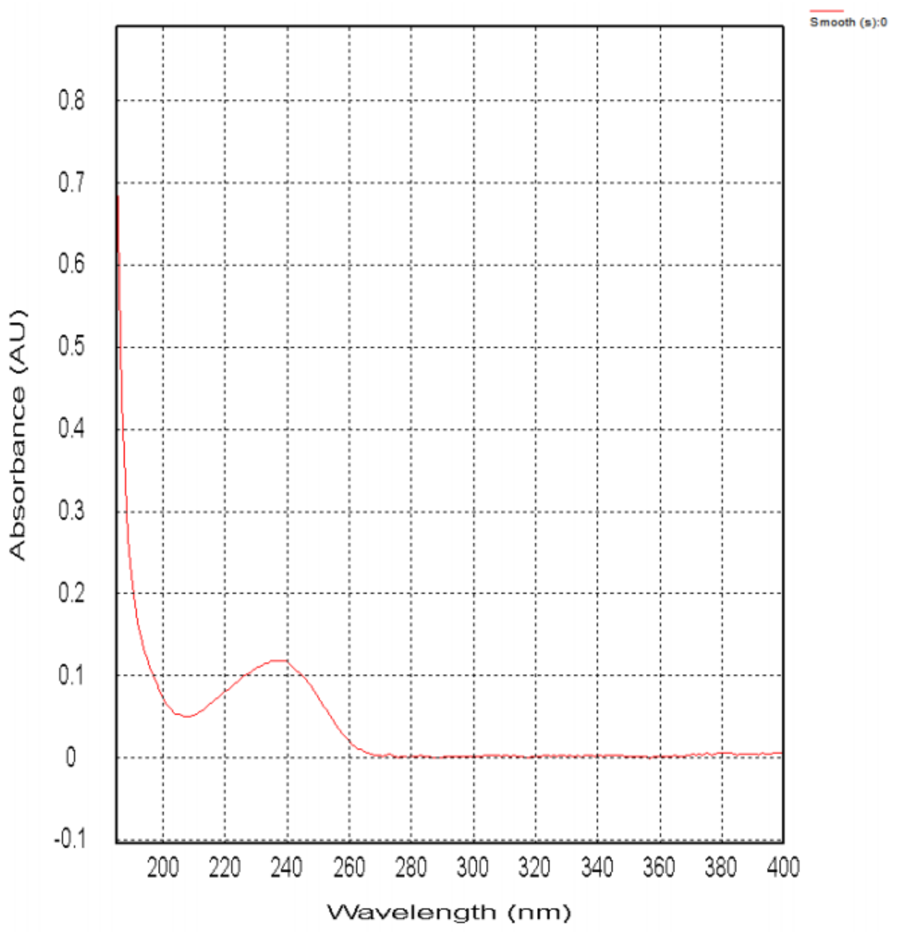

Figure S29. UV spectrum of compound 4

Table S7. Conformational analysis of the optimized isomers of 4 in methanol.

Conformations	G (hartree)	ΔG (kcal/mol)	Boltzmann distributions (%)
4-1	-884.474498	0	50.8
4-2	-884.473938	0.351405264	28.1
4-3	-884.472765	1.08747379	8.1
4-4	-884.472633	1.170305031	7.0
4-5	-884.472476	1.268824007	6.0

Table S8. The coordinates of the optimized conformers of 4.

	4-1			4-2			4-3			4-4			4-5		
C	-2.01525	1.64663	0.37497	-2.01045	1.65142	0.36899	-2.04021	1.62686	0.41898	-2.0389	1.62709	0.41615	-2.03671	1.61436	0.41732
C	-3.05	0.81299	-0.38421	-3.05089	0.82211	-0.38554	-3.04004	0.83793	-0.42878	-3.03152	0.83703	-0.43792	-3.02908	0.83111	-0.44649
C	-3.00089	-0.6601	0.0059	-3.0008	-0.6438	0.00803	-3.00801	-0.65363	-0.11563	-3.00014	-0.65339	-0.11968	-3.00232	-0.65972	-0.13031
C	-1.59647	-1.25273	-0.19649	-1.60002	-1.23846	-0.21488	-1.59652	-1.23788	-0.29131	-1.59664	-1.23851	-0.29596	-1.58796	-1.24237	-0.28798
C	-0.48966	-0.42055	0.54189	-0.4895	-0.42219	0.53357	-0.52118	-0.451	0.53709	-0.52169	-0.45222	0.53249	-0.52059	-0.44819	0.54519
C	-0.63241	1.05299	0.19566	-0.62803	1.05371	0.19485	-0.65007	1.04106	0.2743	-0.64888	1.041	0.27448	-0.65229	1.04092	0.26593
C	0.8575	-0.9672	0.10328	0.85746	-0.96851	0.09366	0.84333	-0.97294	0.12117	0.84226	-0.97312	0.11292	0.8475	-0.96999	0.14054
C	2.003	-0.1237	-0.29933	2.00646	-0.12492	-0.29894	2.00368	-0.10878	-0.16008	2.00384	-0.10855	-0.16159	2.00322	-0.1059	-0.15815
C	1.76547	1.36655	-0.36707	1.7724	1.36597	-0.35755	1.77688	1.38252	-0.11445	1.77881	1.38264	-0.10734	1.76809	1.3849	-0.14789
C	0.38831	1.8259	-0.2065	0.39553	1.82653	-0.20046	0.39104	1.83591	-0.01731	0.39331	1.83654	-0.01167	0.38133	1.83465	-0.05545
O	-3.99183	-1.40093	-0.71032	-3.979	-1.3103	-0.79644	-3.96877	-1.35275	-0.91075	-3.86475	-1.36747	-1.00808	-3.95227	-1.36183	-0.93607
C	3.40106	-0.60445	-0.08384	3.40221	-0.61075	-0.07982	3.4051	-0.60025	0.01679	3.40453	-0.6025	0.01443	3.40699	-0.58609	0.03119
C	3.96808	-1.44195	-0.94932	3.96969	-1.44782	-0.94544	3.78578	-1.125	1.18	3.7832	-1.13404	1.17522	3.78902	-1.08203	1.20657
C	4.06672	-0.11824	1.17444	4.06477	-0.1304	1.18239	4.30748	-0.44517	-1.17444	4.3087	-0.44157	-1.17464	4.30973	-0.45485	-1.16262
O	2.69263	2.14208	-0.55131	2.70197	2.1409	-0.53277	2.71603	2.16519	-0.14076	2.71907	2.16436	-0.12626	2.70391	2.17099	-0.19852
O	1.2053	-0.83557	-1.27488	1.21108	-0.82886	-1.28195	1.24754	-0.74789	-1.23003	1.2484	-0.74025	-1.2362	1.25216	-0.77209	-1.21497
C	-1.58223	-2.74195	0.15738	-1.58763	-2.73424	0.11348	-1.59918	-2.74449	-0.01983	-1.60197	-2.74504	-0.01995	-1.58997	-2.7471	-0.00707
C	-0.55474	-0.57068	2.0798	-0.55563	-0.58672	2.06992	-0.64664	-0.6901	2.05996	-0.64177	-0.69395	2.05558	-0.65457	-0.68296	2.06773
O	-2.097	3.02214	0.03236	-2.0867	3.02555	0.01921	-2.1055	3.01891	0.14629	-2.1036	3.01939	0.1436	-2.02546	2.99458	0.07714
H	-2.2573	1.62059	1.44256	-2.25169	1.63247	1.43702	-2.33149	1.54495	1.4714	-2.33349	1.54467	1.46763	-2.32889	1.5054	1.47159
H	-2.85134	0.90297	-1.46103	-2.86218	0.90085	-1.46341	-2.79398	0.9844	-1.48918	-2.78401	0.96936	-1.49776	-2.7674	0.9907	-1.49904
H	-4.04679	1.21745	-0.1975	-4.04487	1.23004	-0.1921	-4.04401	1.23442	-0.26496	-4.03446	1.24285	-0.2787	-4.03949	1.22032	-0.29332
H	-3.29684	-0.7709	1.05291	-3.2772	-0.74728	1.06513	-3.34708	-0.81906	0.91103	-3.33555	-0.81449	0.91392	-3.35353	-0.82482	0.89239
H	-1.36848	-1.1545	-1.26613	-1.38554	-1.12315	-1.28346	-1.32408	-1.08133	-1.3436	-1.33135	-1.08486	-1.3486	-1.30586	-1.09151	-1.33841
H	1.12117	-1.93475	0.52103	1.12016	-1.93813	0.5071	1.07855	-1.96992	0.48033	1.07694	-1.97219	0.46637	1.08742	-1.9582	0.52019
H	0.23162	2.88497	-0.37409	0.24018	2.88606	-0.36608	0.24481	2.90499	-0.11752	0.24747	2.90603	-0.10803	0.22246	2.8969	-0.19106
H	-3.78815	-1.34298	-1.65285	-4.20132	-2.14902	-0.3786	-3.72621	-1.2447	-1.83958	-4.76867	-1.07473	-0.84419	-3.70193	-1.2484	-1.86211
H	4.97082	-1.824	-0.78934	4.97041	-1.83389	-0.78252	4.80163	-1.4724	1.3392	4.79849	-1.48336	1.33381	4.80639	-1.42064	1.37482
H	3.44284	-1.7722	-1.83757	3.44684	-1.77352	-1.83676	3.09967	-1.21758	2.01561	3.09589	-1.23055	2.00939	3.10229	-1.15887	2.04327
H	5.03733	-0.5959	1.31495	3.43879	-0.34218	2.05589	5.29446	-0.87045	-0.98625	3.87589	-0.93168	-2.05168	4.41973	0.59588	-1.44256

H	4.21387	0.96368	1.14467	5.03115	-0.61514	1.32758	3.87441	-0.94161	-2.04781	4.42695	0.61647	-1.42197	5.29893	-0.86977	-0.96329
H	3.44694	-0.33394	2.05141	4.21966	0.95044	1.15416	4.42363	0.61147	-1.42864	5.29471	-0.86971	-0.98788	3.88027	-0.97495	-2.02389
H	-0.62406	-3.21202	-0.0686	-0.60739	-3.1869	-0.03894	-2.33841	-3.22464	-0.66013	-1.89491	-2.96648	1.00928	-2.32339	-3.23225	-0.65036
H	-2.34831	-3.25702	-0.42132	-2.27915	-3.26363	-0.54403	-1.86851	-2.96843	1.01512	-0.62877	-3.20319	-0.20197	-1.8669	-2.96514	1.02717
H	-1.80321	-2.90893	1.21429	-1.89219	-2.92758	1.14535	-0.63237	-3.20524	-0.22725	-2.32362	-3.22719	-0.67768	-0.62061	-3.20717	-0.2041
H	0.192	0.07182	2.55039	0.21178	0.02702	2.54574	-1.63711	-0.43955	2.43899	-0.45255	-1.74091	2.29643	-1.65086	-0.44384	2.43836
H	-1.53089	-0.30089	2.48198	-1.52168	-0.29168	2.47819	-0.45257	-1.73528	2.30445	0.09308	-0.08812	2.58931	-0.44898	-1.72462	2.31788
H	-0.34708	-1.59957	2.37655	-0.37925	-1.62533	2.35379	0.08257	-0.07901	2.59539	-1.62946	-0.43915	2.43882	0.06283	-0.06072	2.60643
H	-2.03791	3.10326	-0.92845	-2.02868	3.10071	-0.94212	-1.98691	3.15097	-0.80338	-1.98445	3.15061	-0.80612	-2.89367	3.35808	0.28423

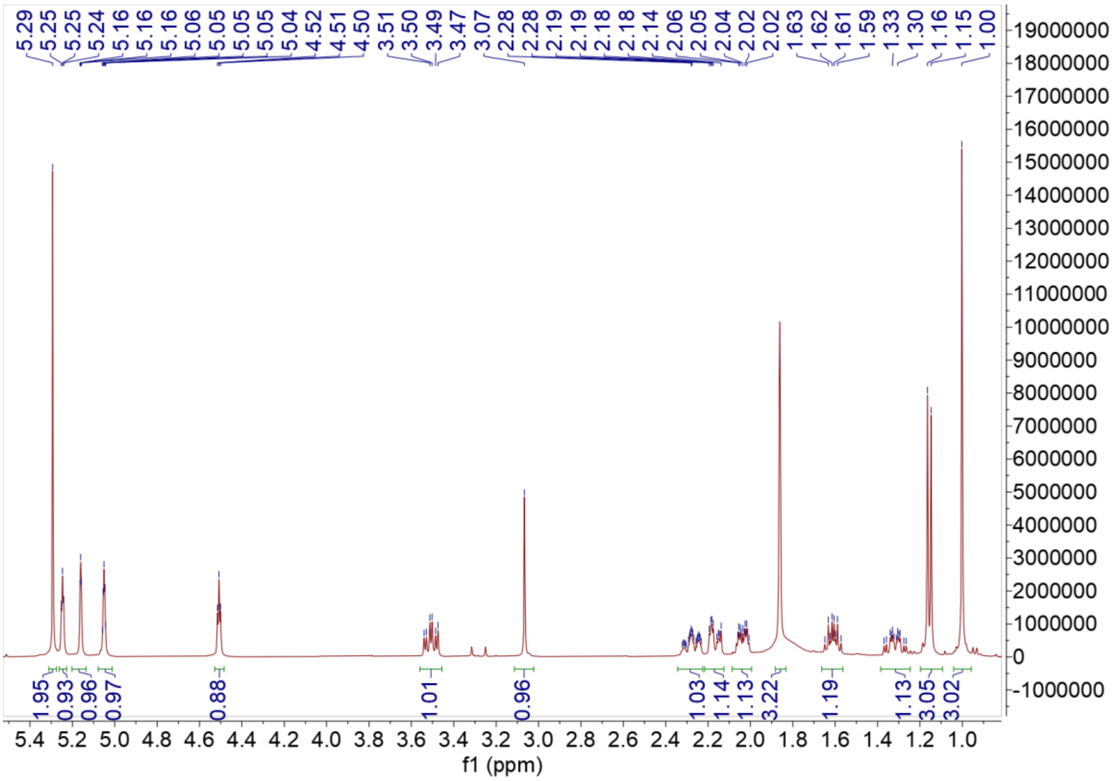

Figure S30. ^{1}H NMR spectrum ($CDCl_3$) of 5

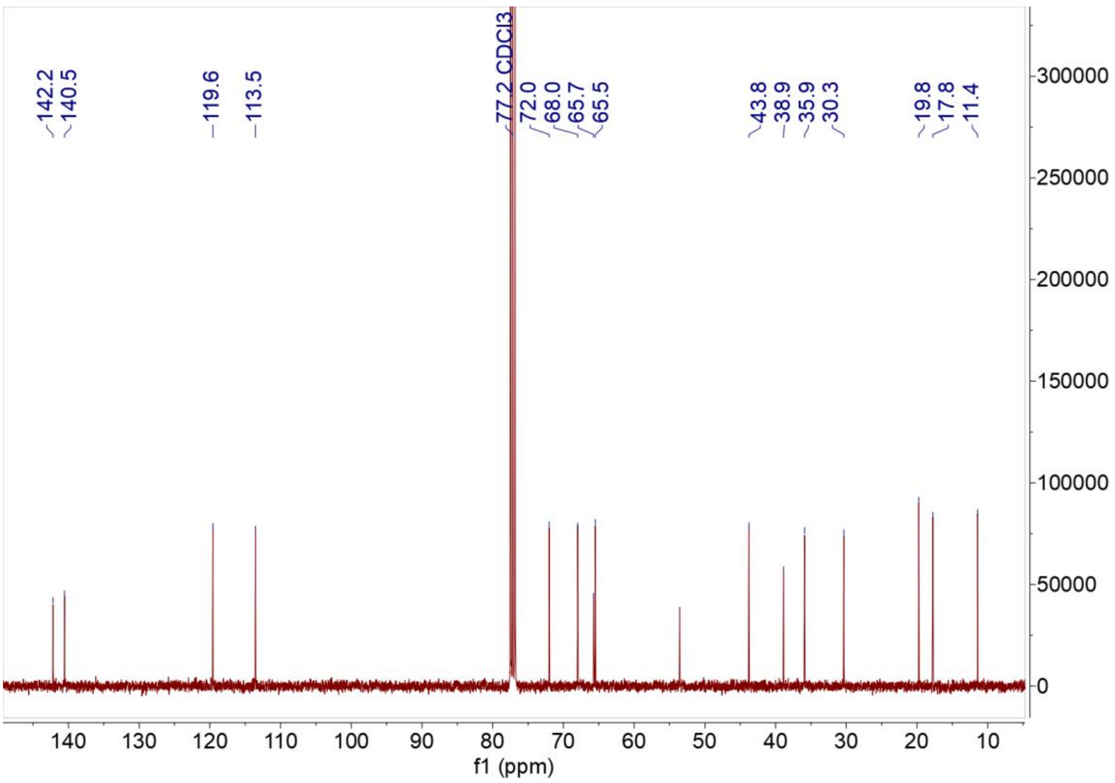

Figure S31. ^{13}C NMR spectrum ($CDCl_3$) of 5

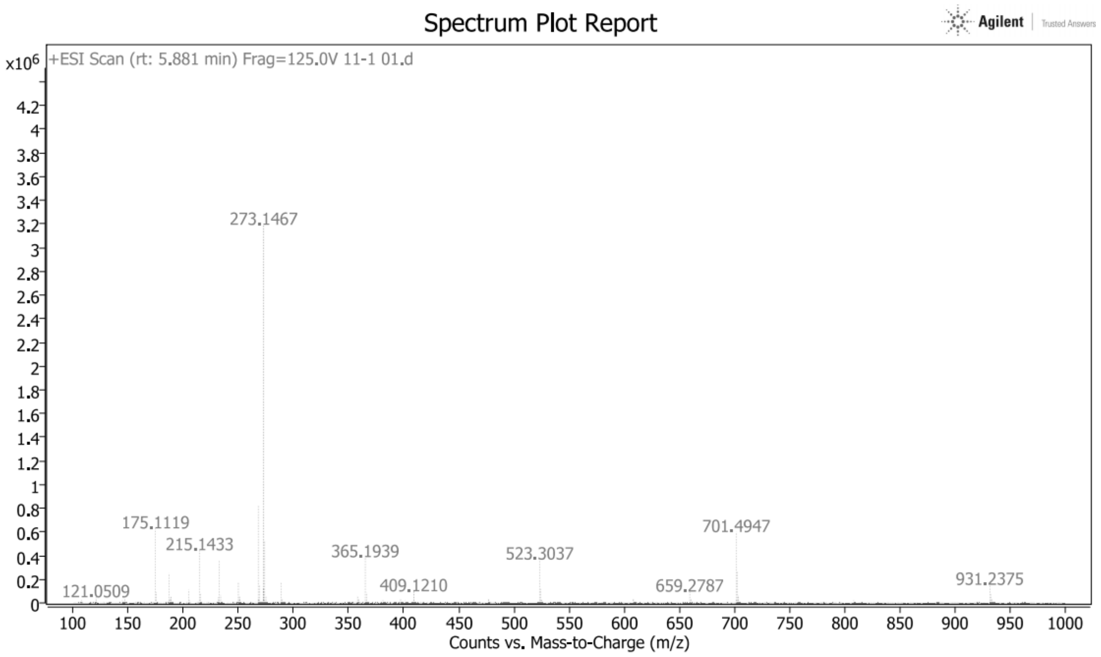

Figure S32. HRESIMS spectrum of 5

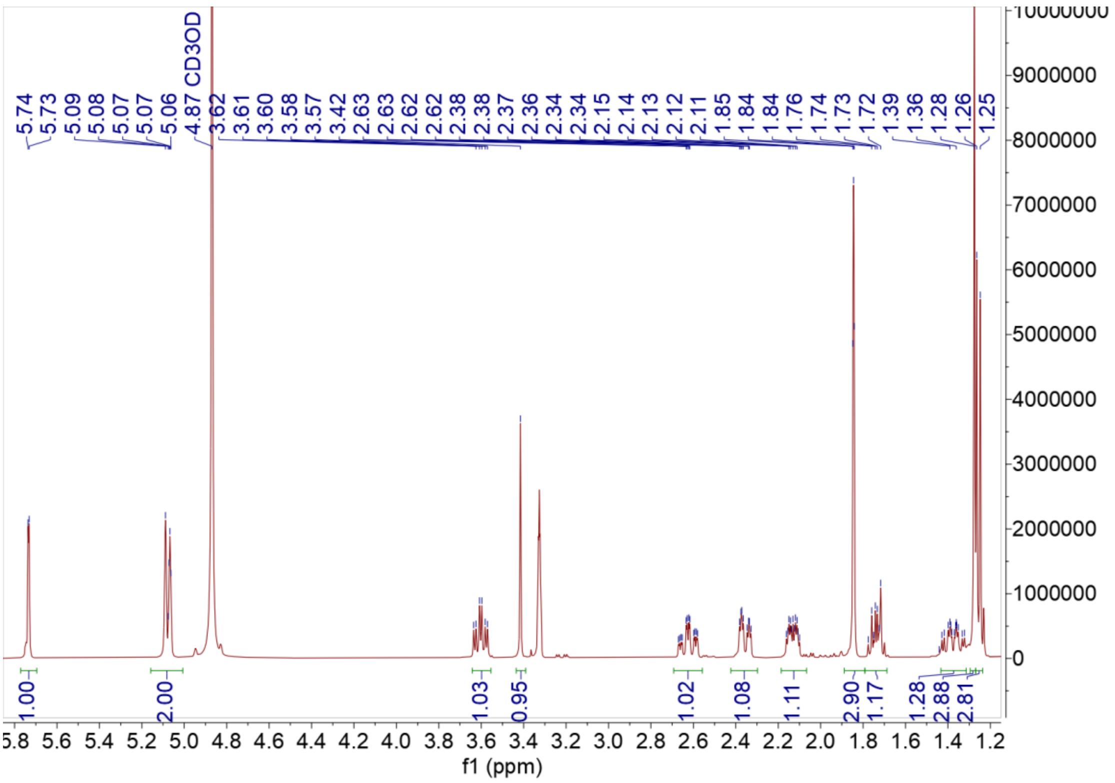

Figure S33. ^{1}H NMR spectrum (CD_3OD) of 6

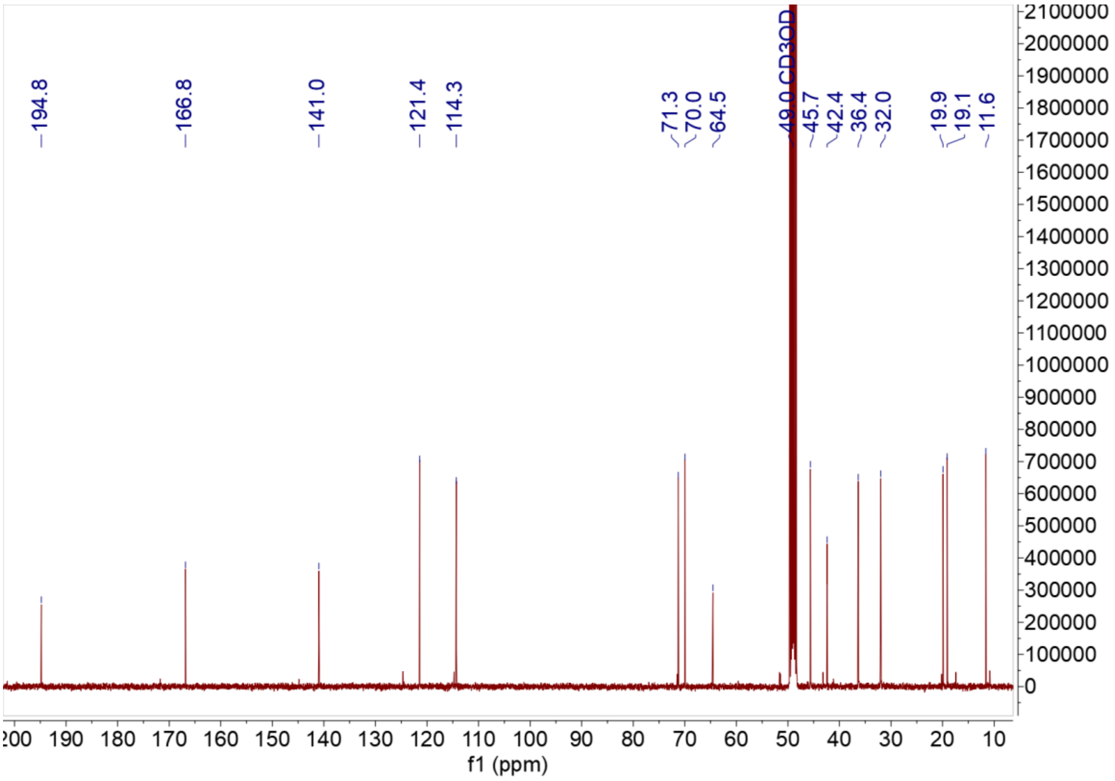

Figure S34. ^{13}C NMR spectrum (CD_3OD) of 6

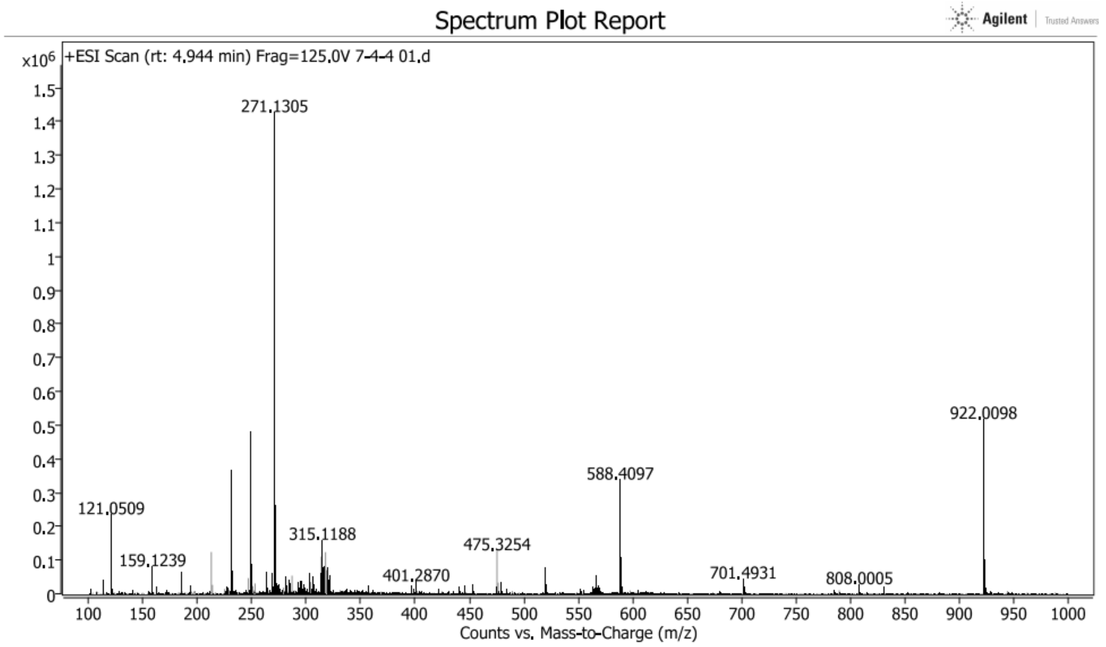

Figure S35. HRESIMS spectrum of 6

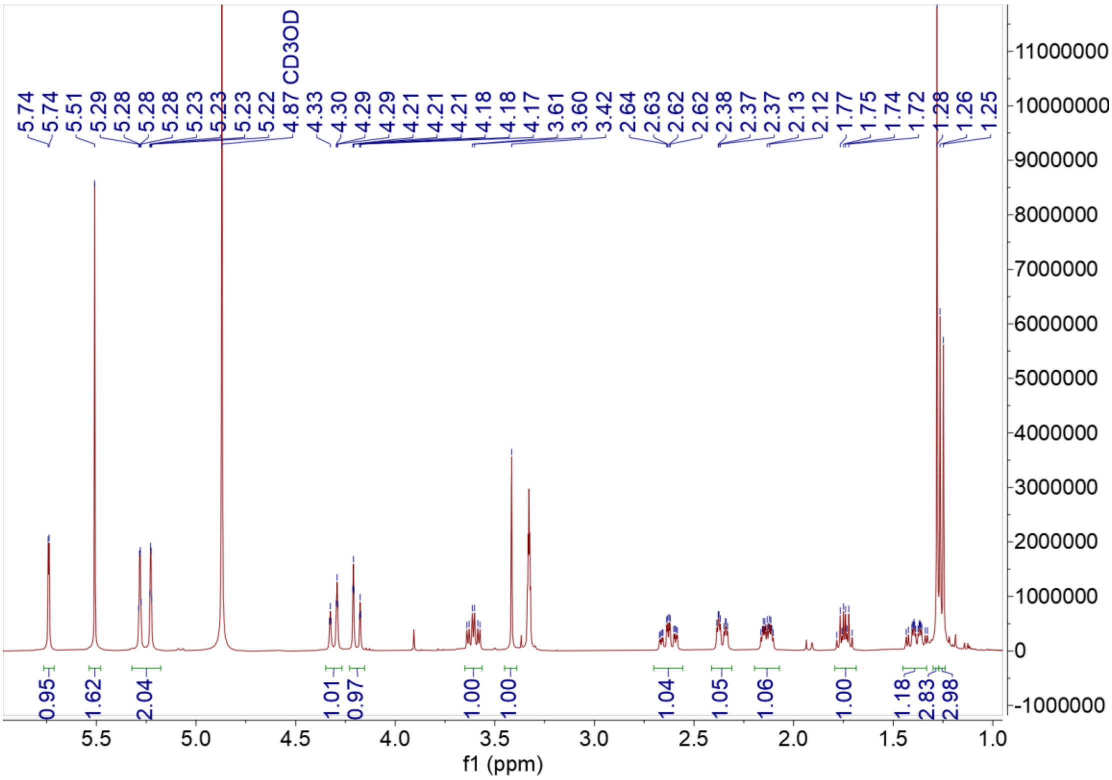

Figure S36. ^{1}H NMR spectrum (CD_3OD) of 7

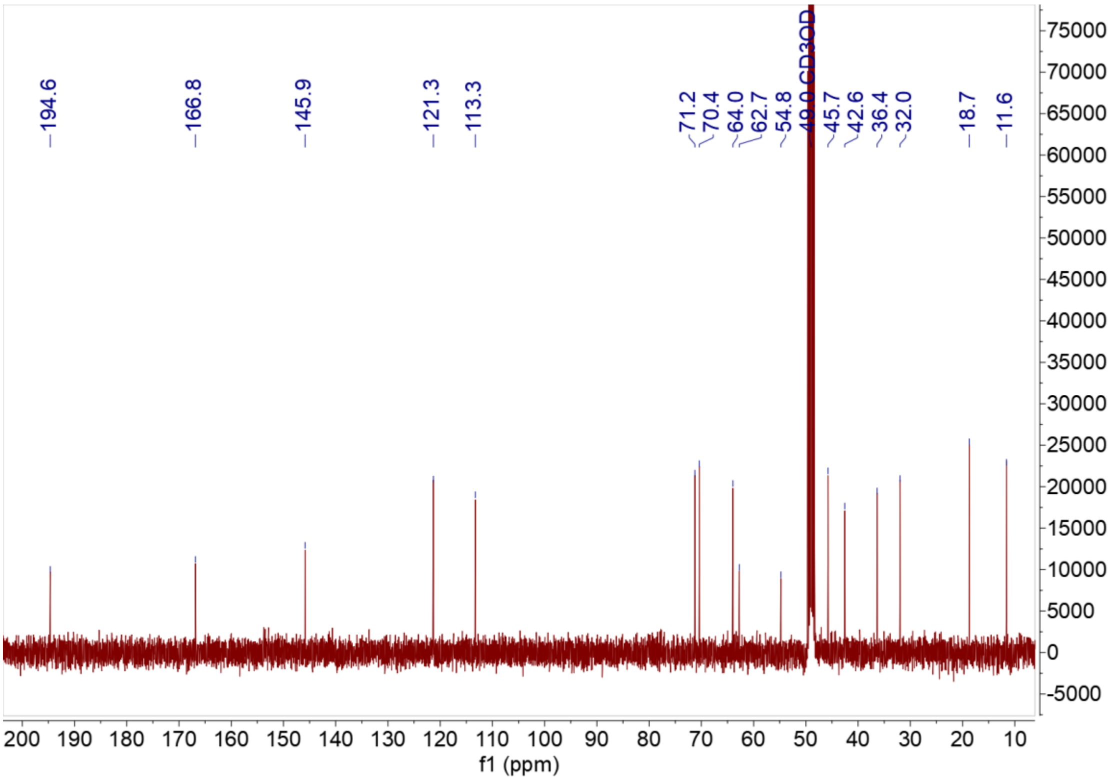

Figure S37. ^{13}C NMR spectrum (CD_3OD) of 7

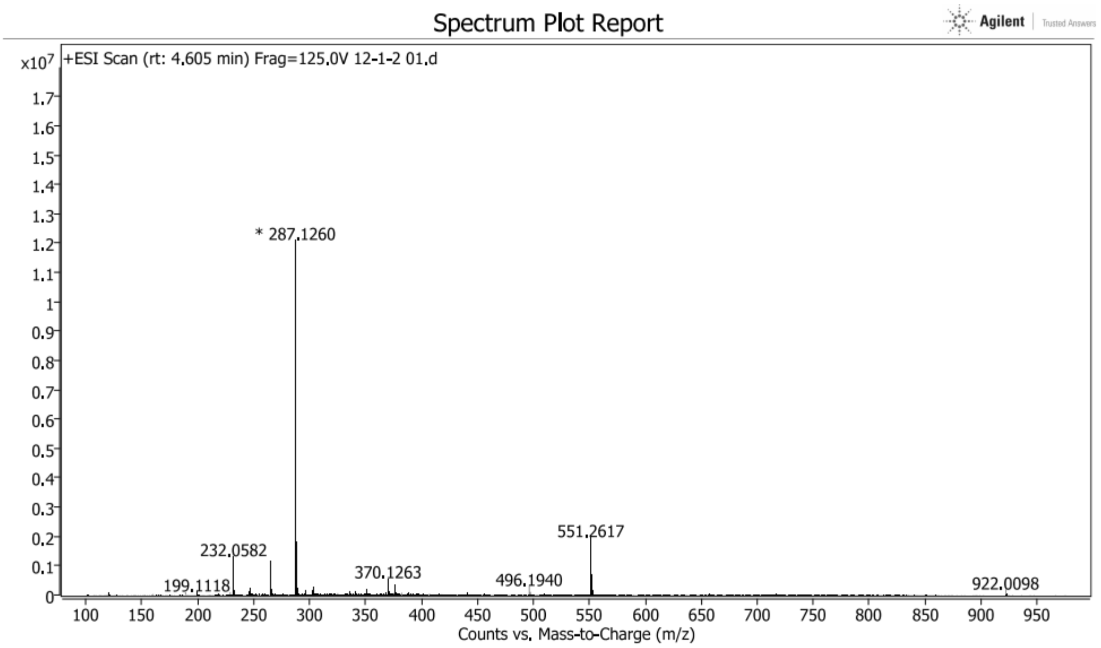

Figure S38. HRESIMS spectrum of 7

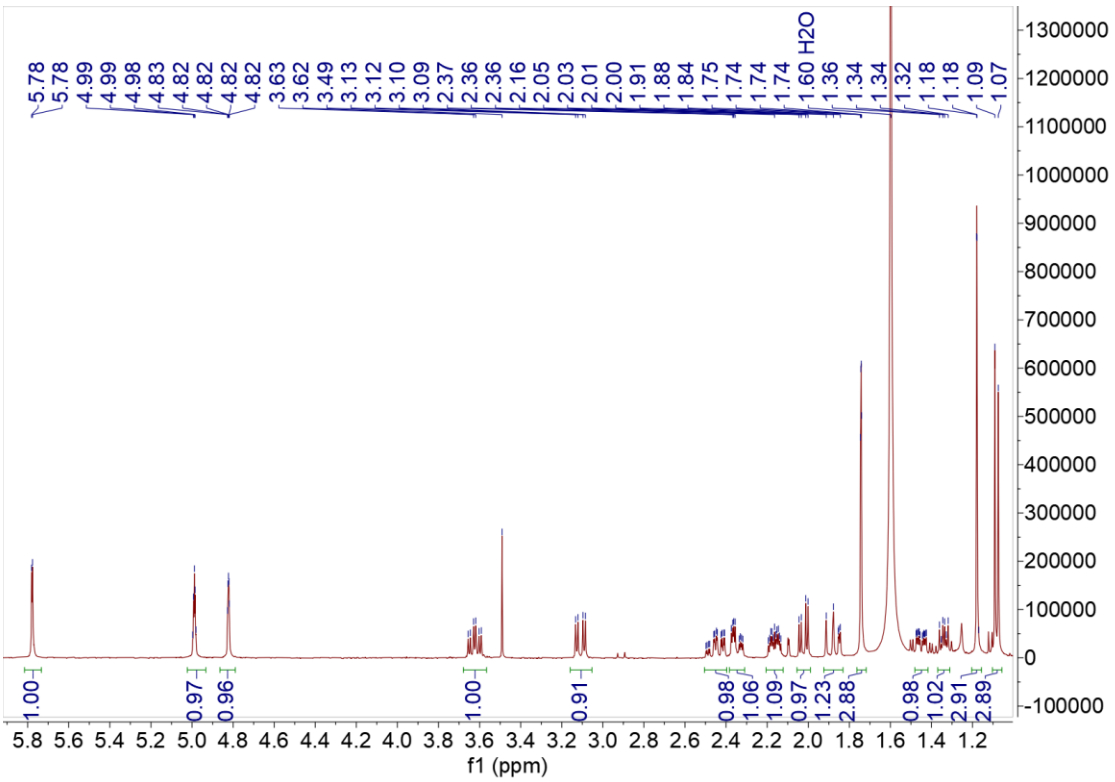

Figure S39. ^{1}H NMR spectrum ($CDCl_3$) of 8

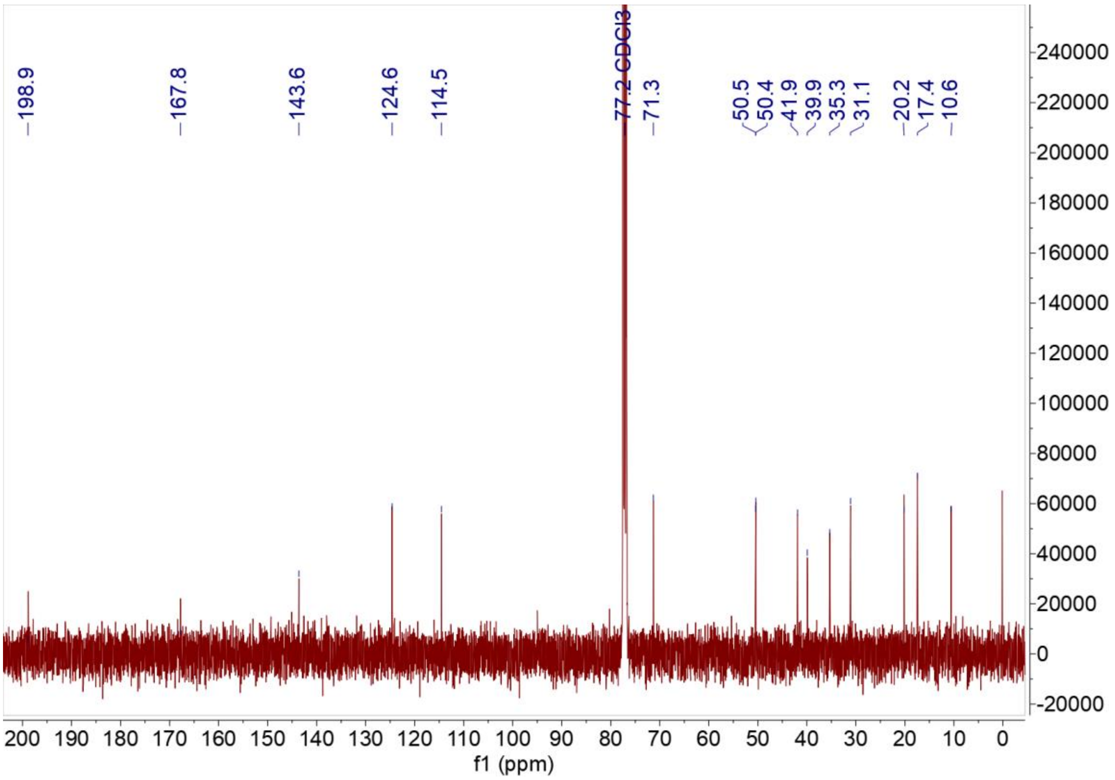

Figure S40. ^{13}C NMR spectrum ($CDCl_3$) of 8

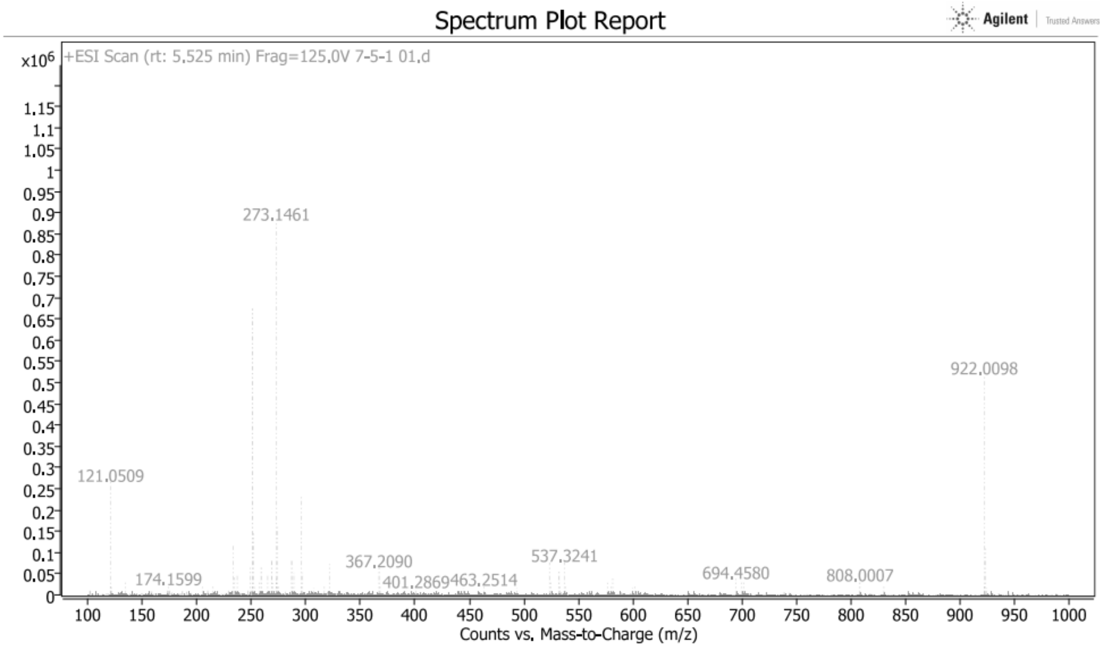

Figure S41. HRESIMS spectrum of 8

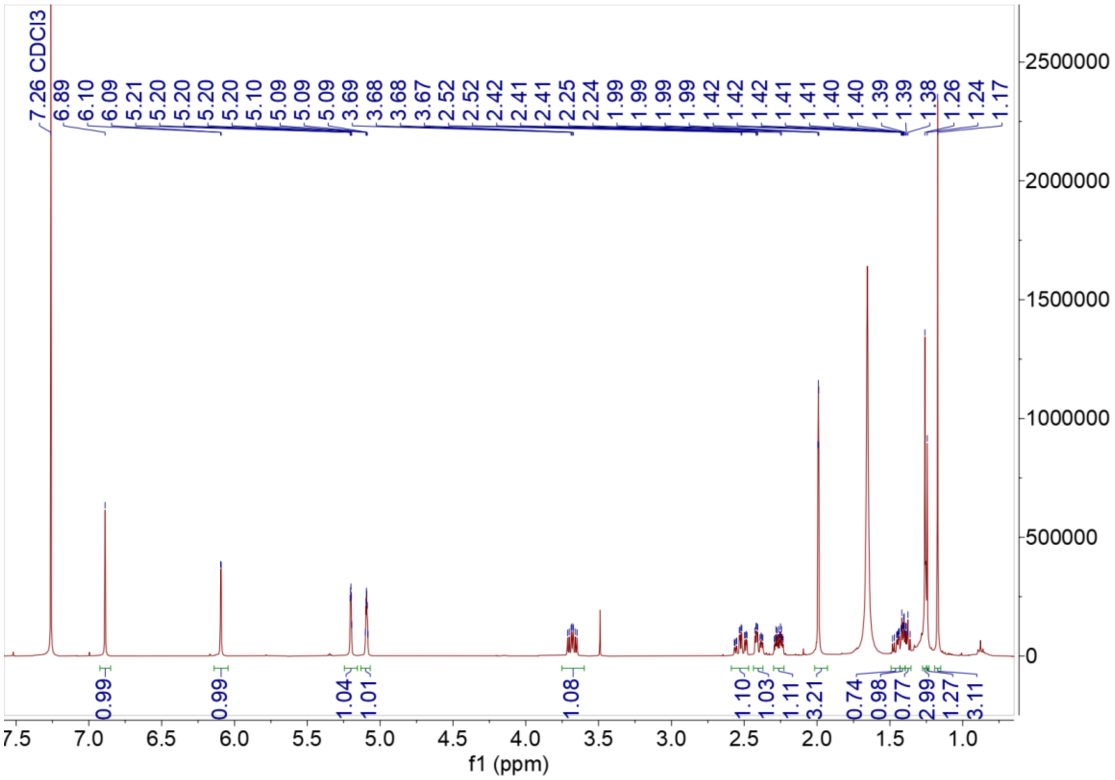

Figure S42. ^{1}H NMR spectrum ($CDCl_3$) of 9

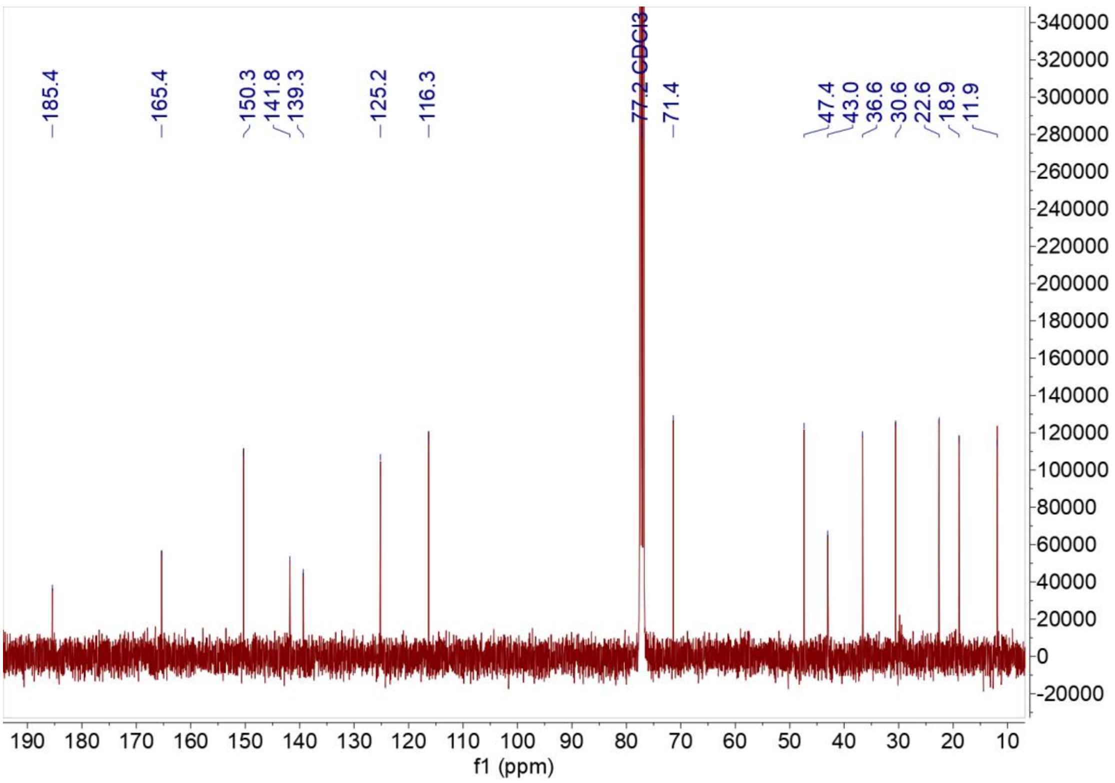

Figure S43. ^{13}C NMR spectrum ($CDCl_3$) of 9

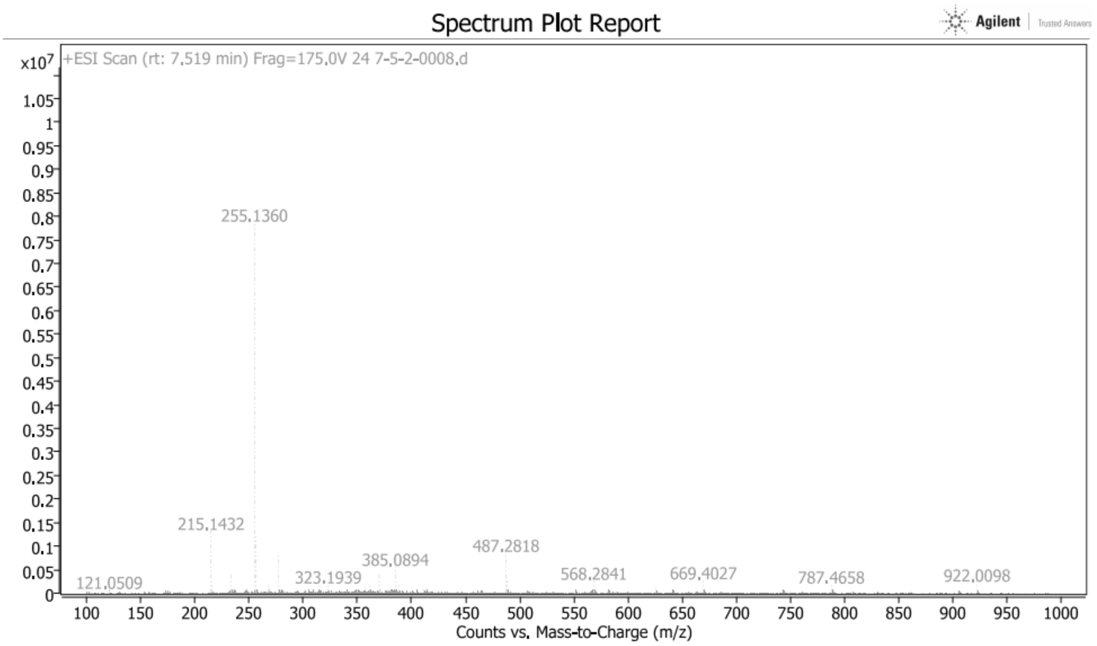

Figure S44. HRESIMS spectrum of 9
